# Supplementary material for: Safety and impact of eculizumab withdrawal in patients with atypical haemolytic uraemic syndrome: protocol for a multicentre, open-label, prospective, single-arm study
Source: BMJ Open. 2022 Sep 19;12(9):e054536. doi: 10.1136/bmjopen-2021-054536 (PMC9486193; doi:10.1136/bmjopen-2021-054536)
Supplement: Supplementary data [file bmjopen-2021-054536supp001.pdf]

[Trust Headed Paper]

SETS aHUS: **S**topping **E**culizumab **T**reatment **S**afely in **aHUS**

Participant Information Sheet (aged 16+)
Version 3.0, 08.02.19

Invitation

You are being invited to take part in a research study. Please read the following information to help you decide if you want to take part. We would like you to understand why we are doing this research and what it means for you. You do not need to make a decision straight away, so please feel free to talk to others about the study if you wish. Please ask us if there is anything that is not clear or if you want to know more.

Please remember that you do not have to take part and your normal healthcare will not be affected in any way, whatever you decide.

Part 1

What is the purpose of this study?

Atypical Haemolytic Uraemic Syndrome (aHUS) is a rare disease. When aHUS occurs the cells that line the blood vessels are damaged and are no longer able to stop blood from clotting. Blood clots form in small vessels, particularly in the kidney, leading to problems with kidney function. Most cases are due to abnormalities in a part of the immune system called the complement system. These abnormalities lead to excessive activation of the complement system, which is responsible for the cell damage and blood clots.

Current standard treatment for aHUS involves a long-term intravenous injection of a drug called Eculizumab every 2 weeks. Eculizumab blocks the body’s complement system and its ability to damage its own vulnerable cells.
Research has shown that Eculizumab is effective in the treatment of aHUS, but the recommendation that Eculizumab treatment should be lifelong is not based on strong evidence and may not be necessary for many patients.

This study hopes to provide evidence for an alternative strategy for treatment of aHUS based on monitoring and treatment re-introduction rather than continuous Eculizumab treatment.

Overall aim: To establish an alternative and safe treatment strategy for patients with aHUS that includes withdrawal of Eculizumab treatment.
Thank you for reading so far – if you are still interested, please read the rest of this leaflet which gives more detailed information about the trial and what will happen if you decide to take part.

Dunn S, *et al. BMJ Open* 2022; 12:e054536. doi: 10.1136/bmjopen-2021-054536

[Trust Headed Paper]

Why have I been chosen to take part?

You have been asked to take part in the study because you have Atypical Haemolytic Uraemic Syndrome and are currently receiving Eculizumab treatment. We are hoping to recruit 30 patients to the withdrawal study and an additional 20 patients who will remain on Eculizumab and complete a series of questionnaires only.

What would taking part involve?

Taking part would involve stopping your Eculizumab treatment and attending 34 safety monitoring visits at hospital over the course of 24 months during which we will assess that you are safe to continue to remain off medication. If you currently receive your treatment at home you would need to be willing to attend the hospital to complete the safety visits.
You will be required to test your urine at home and document the results daily (for the first month), and then three times per week for the remaining 23 months, in a patient diary provided to you by the study team. You will also complete questionnaires on 8 occasions relating to quality of life and measure use of NHS resources and your own out of pocket expenses related to health care.
If you do not want to stop your Eculizumab treatment but still want to participate you can also complete study questionnaires that relate to your quality of life, and measure use of NHS resources and your own out of pocket expenses related to health care. You would only be asked to complete these on 8 occasions over the 24-month period.

There is also a linked interview study that you can take part in whether you decide to withdraw from treatment or continue with Eculizumab treatment. If you would like to receive information about this study you can consent to have your contact details passed to the research team for them to contact you about this.

What are the possible benefits of taking part?

It might mean that you will no longer need to take the Eculizumab treatment and face any potential risks or side effects associated with treatment. Patients are about a thousand times more likely to develop a serious, potentially life threatening infection with meningococcus, a bug that causes meningitis or sepsis. Vaccination, and even antibiotics, do not give complete protection from this. Other serious problems have also been reported in patients taking Eculizumab but we cannot be certain that Eculizumab was the cause of these problems.

Being in the study will mean that you will no longer need bi-weekly infusions and will not have to continue taking additional antibiotics to prevent infection. However, it is possible that you could need to restart and continue to receive Eculizumab treatment if a relapse was to occur.

Dunn S, *et al. BMJ Open* 2022; 12:e054536. doi: 10.1136/bmjopen-2021-054536

[Trust Headed Paper]

What are the possible risks of taking part?

The withdrawal of Eculizumab treatment could lead to a relapse of atypical HUS and relapse associated complications. When a relapse is diagnosed, your Eculizumab treatment will be restarted within 24 hours of presentation. It is essential that you present to a hospital with your patient card as soon as you begin to feel unwell or the home urine test shows an increase in the level of blood. This is to ensure that the Eculizumab is re-started as soon as possible to reduce the likelihood of kidney damage and associated complications. If your treatment is re-started, you will receive your first infusion in the hospital. You will also have the antibiotics restarted to protect you from infection. If you need to be put back on to Eculizumab treatment you can decide to receive your infusions in hospital or at home. This can be arranged with your clinical team if you relapse while in the study.

If a relapse occurs this could lead to:

- A drop in kidney function
- Other problems related to the disease which can affect organs such as the pancreas or nervous system

The evidence that is available at the moment suggests that if Eculizumab is reintroduced quickly these problems can be avoided. This is not proven, and this study will test whether this is true.

If, during the trial, you decide to travel outside of the country, we ask that you only travel to countries where Eculizumab is available, so treatment can be re-started immediately as required. You **MUST** first inform your hospital to check whether Eculizumab is available in the country that you plan to travel to and inform them of the dates that you will be outside of the country. While the cost of the Eculizumab will be covered by NHS England you must ensure that you have appropriate travel insurance and inform your insurance company that you are taking part in the trial. This is to ensure that all other treatment costs and hospital stays abroad are covered by your own insurance should you relapse. We have developed a travel guide to be followed should you decide to travel outside of the country while you are participating in the withdrawal study.

To minimise the risks of taking part in the study, we will only include you in the study if you:

- Have been on Eculizumab treatment for at least 6 months;
- Are in remission;
- Have a stable kidney function;
- Are willing to attend for safety monitoring assessments;
- Are willing to travel only to countries that can supply Eculizumab (to be confirmed with co-ordinating centre prior to travel);
- Are able to perform and record self-monitoring urinalysis.

Dunn S, *et al. BMJ Open* 2022; 12:e054536. doi: 10.1136/bmjopen-2021-054536

[Trust Headed Paper]

Sexually active females of child bearing age must:

- Have a negative pregnancy test at screening and be using an effective contraception for the duration of the study (please ask your doctor/nurse).

  OR

- fulfil one of the following criteria:
  - Be post-menopausal
  - Have undergone surgical sterilisation

You will not be able to take part if you:

- have lost a previous transplant kidney to recurrent aHUS;
- Are currently or are planning pregnancy;
- Are unable to comply with safety monitoring assessments;
- Are currently participating in another clinical trial (not including participation in aHUS registries)

What will happen to me if I take part?

If you consent to withdraw from Eculizumab Treatment, you will complete the following visits:

Screening Visit

You will be asked to attend a screening visit at the hospital or clinic where you usually receive your Eculizumab treatment or if you receive your treatment at home, you will be asked to attend the hospital where your clinician is based. If you decide to take part you will complete a consent form. You will be given a unique patient ID number that will be written on your consent form and all questionnaires and samples that you provide in the study. This ID number will only be linked to you at your hospital and so nobody outside of your direct clinical care team will know that the information and samples belong to you.
If you are a female of child bearing age you will be required to have a pregnancy test at the screening visit after you have signed the consent form. You will receive your last Eculizumab infusion at the screening visit. Please be aware that you will continue to take your meningococcal prophylaxis for 4 weeks following this last Eculizumab infusion.
We will also collect information from your medical notes about your medical history after you have signed the consent form.

Baseline Visit

Your baseline visit will be 2 weeks (+/-2 days) after your screening visit on the day that you would usually receive your next dose of Eculizumab. You will not receive the infusion but will

Dunn S, *et al. BMJ Open* 2022; 12:e054536. doi: 10.1136/bmjopen-2021-054536

[Trust Headed Paper]

attend your usual hospital or clinic to have your blood pressure and temperature checked. You will also have a blood and urine test and complete questionnaires about your health.

Study Visits

You will be reviewed at the hospital weekly for the first month, then alternate weeks until month 6, then monthly thereafter until the end of the study period (month 24).
At each study visit you will have a blood and urine test and answer questions about how your health has been since your last visit.

You will also be required to complete questionnaires at your hospital visits twice in month 1, and once in the following months, 3, 6, 9, 12, 18 & 24. These will ask about your health, quality of life, medications and about use of NHS services and your out of pocket expenses relating to health care. It should take between 20 and 30 minutes to complete these questionnaires each time.

Overall you will be attending 34 visits over the 24 month period which includes the screening and baseline visit. You will not be required to attend more visits than you would usually if you were to stay on your current treatment. If you currently receive your Eculizumab infusions at home you will need to come in to the hospital for the study visits as these cannot be carried out at your home.

You will be required to carry out urine tests at home and to document this in a patient diary. You will be given a 30-minute training session during your baseline visit that will explain everything that you will need to do to carry this out at home.

You will also be given a small card to carry which will include information on the trial. If you attend **ANY** hospital or clinic for treatment outside of your scheduled follow-up visits you **MUS**T give the doctor this card so that they are able to contact the trial team.

If you continue with your Eculizumab treatment and consent to completing the questionnaires only;
You will receive the questionnaires by post on 8 occasions over the 24-month period. This should take you around 20-30 minutes to complete. You will be required to post the completed questionnaires back to the research team at Newcastle University using the pre-paid envelope supplied. The questionnaires will not contain any identifiable information relating to you as your unique patient ID will be written on the questionnaires when you receive them.

Will participating in research affect my treatment?

If you decide to take part in the withdrawal component of the trial then you will no longer receive your Eculizumab treatment. If you decide to take part in the questionnaire part of the trial only, your Eculizumab treatment will remain the same.

Dunn S, *et al. BMJ Open* 2022; 12:e054536. doi: 10.1136/bmjopen-2021-054536

[Trust Headed Paper]

What will happen if I don't want to carry on with the study?

You may withdraw from the study at any time without giving a reason. If you decide to go back on to Eculizumab treatment we would like you to continue to be followed up by the study team. You may wish to withdraw completely from the study and further follow up and so we would not collect any further information about you to be used in the study. However, we would like to use the information and blood samples previously provided. If you decide that you don't want any of the information or blood samples already provided to be used in the study please contact a member of the study team so it can be removed from analysis.

Will I be paid for taking part?

If you receive re-imbursement for travel to clinics this will continue. If you currently receive your Eculizumab treatment at home, you may qualify to be reimbursed for travel to clinics for your follow up visits. Your doctor can give you more information about this. You will not be paid for taking part in this study.

Part 2

Will my GP be told about my involvement in this study?

If you decide to take part in this study and consent to have your GP informed, then we will inform your GP. Your participation in the study will also be noted in your medical records.

Will my taking part in research be kept confidential?

All of the data we collect will be kept strictly confidential and in accordance with the General Data Protection Regulation (GDPR).

The Newcastle Upon Tyne Hospitals NHS Foundation Trust (NUTH) is the sponsor for this study based in the United Kingdom and will act as the "data controller" for this study. **They are responsible for looking after your information and using it properly.** This study is managed on behalf of the sponsor by the Newcastle Clinical Trials Unit (NCTU) who will act as the "data processor". As data processor, NCTU are responsible for processing personal data on behalf of the data controller. The Newcastle Clinical Trials Unit based at Newcastle University would like to receive a copy of your consent form for safety purposes. This will be destroyed once it has been reviewed. No other identifiable information will be received by the Newcastle Clinical Trials Unit

Your rights to access, change or move your information are limited, as we need to manage your information in specific ways in order for the research to be reliable and accurate. If you withdraw from the study, we will keep the information about you that we have already

[Trust Headed Paper]

obtained. To safeguard your rights, we will use the minimum personally-identifiable information possible.

You can find out more about how we use your information at www.newcastle-hospitals.org.uk/about-us/freedom-of-information_how-we-use-information.aspx

To find out more information about research and general use of patient information please refer to the Health Research Authority Website at www.hra.nhs.uk/information-about-patients

The local study team at [NHS site] will use your name, [NHS number/CHI number] and contacts details to contact you about the research study, and make sure that relevant information about the study is recorded for your care, and to oversee the quality of the study. Individuals from the sponsor, Newcastle Clinical Trials Unit and regulatory organisations may look at your medical and research records to check the accuracy of the research study. The local study team will pass these details to the sponsor or the Newcastle Clinical Trials Unit along with information collected from you and/or your medical records. The only people at sponsor or the Newcastle Clinical Trials Unit who will have access to information that identifies you will be people who need to audit the data collection process. The people who analyse the information will not be able to identify you and will not be able to find out your name, [NHS number/CHI number] or contact details.

The local study team at [NHS site] will keep identifiable information about you from this study for 5 years after the study has finished.

When you agree to take part in a research study, the information about your health and care may be provided to researchers running other research studies in this organisation and in other organisations. These organisations may be universities, NHS organisations or companies involved in health and care research in this country or abroad. Your information will only be used by organisations and researchers to conduct research in accordance with the UK Policy Framework for Health and Social Care Research and will not contain any personal identifiable information such as your name, [NHS number/CHI number] or contact details.

This information will not identify you and will not be combined with other information in a way that could identify you. The information will only be used for the purpose of health and care research and cannot be used to contact you or to affect your care. It will not be used to make decisions about future services available to you, such as insurance.

The blood samples that you provide will be sent to Newcastle University to be analysed and will have your unique ID number on them but no personal identifiable information attached. Once the study is finished, these samples will be stored at the Newcastle University biobank and may be used in future aHUS research. Please let us know if you do not want your samples used in future research.

Dunn S, *et al. BMJ Open* 2022; 12:e054536. doi: 10.1136/bmjopen-2021-054536

[Trust Headed Paper]

What will happen to the results of the research study?

Whenever possible we will publish the results of our studies in scientific journals. We also plan to present data at scientific conferences. You will not be named in any publication or presentation of the study results. We would also like to send you a newsletter with a summary of our results. Please let the research team know if you want to receive the newsletter. Results will also be available at the end of the study on the atypical HUS website which can be found at the following web address:
http://www.atypicalhus.co.uk/

Who is organising and funding the research?

This study is funded by the National Institute for Health Research (NIHR). The study is sponsored and indemnified by the Newcastle Upon Tyne NHS Foundation Trust and indemnified by Newcastle University. The Newcastle Clinical Trials Unit is managing the study.

Who has reviewed the study?

All research in the NHS is looked at by an independent group of people called a NHS Research Ethics Committee (REC). This is to protect your interests. This study has been reviewed and given a favourable opinion by the North East – Tyne & Wear South Research Ethics Committee and has been approved by the NHS Health Research Authority (HRA). The study has also been given approval by the Medicines and Healthcare products Regulatory Agency (MHRA).

What if relevant new information becomes available?

The study team will ensure that you are receiving the most appropriate and up to date medical care that you require.

What if something goes wrong?

If you have a concern about any aspect of the study please contact your local doctor (see contact details below). Alternatively, you can contact one of the researchers running this study and discuss your concerns.

Your local contact people for the study are:

Contact Details of local PI:	Contact details of local Research Nurse:
Name:	Name:
Address:	Address:
Phone:	Phone:
Email:	Email:

Dunn S, *et al. BMJ Open* 2022; 12:e054536. doi: 10.1136/bmjopen-2021-054536

[Trust Headed Paper]

The Newcastle Trial Team contact is:

Contact Details of Trial Manager:
Name: Sarah Dunn
Address: 1-2 Claremont Terrace,
NCTU, Newcastle University, NE2 4AE
Phone: 0191 208 2521
Email: sarah.dunn2@ncl.ac.uk

If you are still unhappy and wish to complain formally and confidentially you can do this through the NHS complaints procedure by speaking to a member of the PALS (Patient Advise and Liaison Service) on 0800 0320 202 or by visiting www.PALS.nhs.uk.

In the event that something goes wrong and you are harmed during the research due to someone's negligence, then you may have grounds for a legal action for compensation against Newcastle upon-Tyne Hospitals NHS Foundation Trust but you may have to pay your legal costs.

How have patients and the public been involved in this study?

Our patient representative group has helped to design the study and study documents.

The group will meet when the trial is near completion to have input into the interpretation of the results. Their views of withdrawal and the safety results from the study will be considered. This will inform the publication of results. The groups views on the dissemination of results will also be considered.

Thank you for taking time to read this information sheet

Dunn S, *et al. BMJ Open* 2022; 12:e054536. doi: 10.1136/bmjopen-2021-054536
