# Supplementary material for: Safety and impact of eculizumab withdrawal in patients with atypical haemolytic uraemic syndrome: protocol for a multicentre, open-label, prospective, single-arm study
Source: BMJ Open. 2022 Sep 19;12(9):e054536. doi: 10.1136/bmjopen-2021-054536 (PMC9486193; doi:10.1136/bmjopen-2021-054536)
Supplement: Supplementary data [file bmjopen-2021-054536supp002.pdf]

SETS aHUS: Stopping Eculizumab Treatment Safely in aHUS

The holder of this card has a rare disease known as atypical Haemolytic Uraemic Syndrome and is taking part in a clinical trial to assess the safe withdrawal of Eculizumab treatment. Because of this, the holder of this card may suffer a relapse.

If the holder presents unwell, however minor the illness, please evaluate immediately and obtain the following Laboratory investigations as their Eculizumab treatment and prophylactic antibiotics may need to be re-started as soon as possible and within 24hrs:

• U&E • FBC • LDH

If the results are abnormal, immediately contact the local medical team and refer to the UK National aHUS Service website for advice. Contact details on reverse side.

Dunn S, *et al. BMJ Open* 2022; 12:e054536. doi: 10.1136/bmjopen-2021-054536

SETS aHUS: Stopping Eculizumab Treatment Safely in aHUS

Patients Local Medical team: +44 (0)............................

UK National aHUS Service: +44 (0)191 2820385

(from 9am to 5pm GMT)

Outside of these hours, please contact the Newcastle Hospitals Switchboard and request to speak to the clinician on call for the aHUS Service on +44 (0)191 233 6161

www.atypicalhus.co.uk

Patient Card_Version 1.0_08.02.2018 Page 2 of 2
