# Supplementary material for: Safety and impact of eculizumab withdrawal in patients with atypical haemolytic uraemic syndrome: protocol for a multicentre, open-label, prospective, single-arm study
Source: BMJ Open. 2022 Sep 19;12(9):e054536. doi: 10.1136/bmjopen-2021-054536 (PMC9486193; doi:10.1136/bmjopen-2021-054536)
Supplement: Supplementary data [file bmjopen-2021-054536supp003.pdf]

Primary registry and trial identifying number	ISRCTN<br>ISRCTN17503205
Date of registration in primary registry	20 April 2018
Secondary identifying numbers	EudraCT: 2017-003916-37
Source(s) of monetary or material support	NIHR HTA
Primary sponsor	Newcastle Upon Tyne Hospitals NHS FT – Christopher Price<br>christoper.price6@nhs.uk
Secondary sponsor(s)	N/A
Contact for public queries	Trial Manager – Sarah Dunn sarah.dunn2@newcastle.ac.uk
Contact for scientific queries	Chief Investigator – Professor Neil Sheerin neil.sheerin@newcastle.ac.uk
Public title	Stopping Eculizumab Treatment Safely in aHUS (SETS aHUS)
Scientific title	Multicentre, open label, prospective, single arm study of safety impact of Eculizumab withdrawal
Countries of recruitment	England and Scotland
Health condition(s) or problem(s) studied	atypical Haemolytic Uraemic Syndrome
Intervention(s)	Withdrawal of Eculizumab
Key inclusion and exclusion criteria	Key inclusion criteria:<br>• Age ≥2+ years of age,<br>• On Eculizumab treatment for at least 6 months,<br>• In remission with no evidence of ongoing microangiopathic haemolytic anaemia (MAHA) activity at screening defined by:<br>- Platelet count > lower limit of normal as determined by local reference range,<br>- Lactate Dehydrogenase (LDH) <x2 upper limit of normal as determined by local lab reference ranges,<br>• Normal renal function or Chronic Kidney Disease (CKD) stages 1-3,<br>• Absence of decline of renal function confirmed by review of available assessments of renal function for the preceding 6 months by the Chief

	Investigator (CI) and clinical members of the Trial Management Group (TMG).<br>Key Exclusion Criteria:<br>• Severe non-renal disease manifestations at initial presentation with aHUS, which in the opinion of the Chief Investigator and/or the clinical members of the TMG makes the risk of treatment withdrawal unacceptable,<br>• Current or planned pregnancy within the study duration,<br>• Unable to give informed consent or assent, or unable to obtain parent/guardian consent if under 16 years of age,<br>• Current participation in another clinical trial (not including participation in aHUS registries),<br>• Severe, uncontrolled hypertension (systolic blood pressure >160 mmHg) that is likely to induce at TMA.
Study type	Allocation: non-randomized<br>Masking: open label<br>Primary purpose: Safety<br>Phase IIb
Date of first enrolment	November 2018
Target sample size	50: 30 withdrawal and 20 non-withdrawal
Recruitment status	Recruiting
Primary outcome(s)	To determine the safety of Eculizumab withdrawal in patients with aHUS
Key secondary outcomes	1. The effectiveness of a monitoring protocol to detect disease relapse following withdrawal of Eculizumab assessed by:<br>1.1. The proportion of patients who relapse and restart Eculizumab without the development of a TMA-related SAE<br>1.2. The time from the first clinical feature (symptom, positive urinalysis or laboratory result) of a relapse of TMA and the re-introduction of Eculizumab<br>This outcome is ongoing and not measured at any particular timepoint<br>2. The relapse rate after withdrawal of Eculizumab as determined by the proportion of patients who relapse after Eculizumab is withdrawn. This outcome is ongoing and not measured at any particular time point. A patient could relapse at any point in the 2 years participation period.<br>3. The proportion of patients, currently on long-term treatment with Eculizumab, who can be maintained off treatment. This outcome is measured at the end of the trial when all relapse data is collected. A patient could relapse at any point in the 2 years participation period.<br>4. The period from withdrawal to relapse in those patients who restart treatment. This outcome is measured at the end of the trial when all relapse data is collected.<br>5. The change in estimated GFR as calculated by the CKD-EPI or modified Schwartz equations over the course of the study from baseline (day 0) to

	end of the study. This outcome is calculated at the end of the trial when all GFR data is collected. GFR data is collected at all 34 visits.<br>6. Important clinical and laboratory indicators of imminent relapse by review of reported symptoms, physical signs, urinalysis and laboratory results prior to the diagnosis of a relapse. This outcome will be assessed at the end of the trial when all relapse data is collected. Those who have relapsed will have all data preceding relapse reviewed to establish a relapse profile.<br>7. The costs and health outcomes (measured in terms of adverse events and quality-adjusted life years [QALYs]) for patients on standard care (not withdrawing from Eculizumab treatment) over the two-year trial duration:<br>7.1. Healthcare Utilisation Questionnaires for non-withdrawal participants at Day 0, 14, 70,154, 252, 336, 504 and 672.<br>7.2. Adverse Event Assessment at every visit from Day 7 (32 visits) for withdrawal participants.<br>8. QALYs estimated from responses to the EQ-5D-5L, and SF-36 and determinants of QALYs/utilities over the 24-month follow-up period. at Day 0, 14, 70,154, 252, 336, 504 and 672.<br>9. Model-based estimate of the costs and health consequences, with results presented in terms of cost per QALY gained, over the estimated lifetime of patients withdrawing from treatment compared with standard care.
