# Supplementary material for: Safety and impact of eculizumab withdrawal in patients with atypical haemolytic uraemic syndrome: protocol for a multicentre, open-label, prospective, single-arm study
Source: BMJ Open. 2022 Sep 19;12(9):e054536. doi: 10.1136/bmjopen-2021-054536 (PMC9486193; doi:10.1136/bmjopen-2021-054536)
Supplement: Author's manuscript [file bmjopen-2021-054536.draft_revisions.pdf]

BMJ Open

BMJ Open is committed to open peer review. As part of this commitment we make the peer review history of every article we publish publicly available.

When an article is published we post the peer reviewers' comments and the authors' responses online. We also post the versions of the paper that were used during peer review. These are the versions that the peer review comments apply to.

The versions of the paper that follow are the versions that were submitted during the peer review process. They are not the versions of record or the final published versions. They should not be cited or distributed as the published version of this manuscript.

BMJ Open is an open access journal and the full, final, typeset and author-corrected version of record of the manuscript is available on our site with no access controls, subscription charges or pay-per-view fees (http://bmjopen.bmj.com).

If you have any questions on BMJ Open's open peer review process please email info.bmjopen@bmj.com

BMJ Open

Protocol for a multicentre, open label, prospective, single arm study of the safety and impact of eculizumab withdrawal in patients with atypical haemolytic uraemic syndrome

Journal:	*BMJ Open*
Manuscript ID	bmjopen-2021-054536
Article Type:	Protocol
Date Submitted by the Author:	15-Jun-2021

Complete List of Authors:	Dunn, Sarah; Newcastle University, Newcastle Clinical Trials Unit<br>Brocklebank, Victoria; Newcastle University, National Renal Complement Therapeutics Centre<br>Bryant, Andrew; Newcastle University, Population Health Sciences Institute<br>Carnell, Sonya; Newcastle University, Newcastle Clinical Trials Unit<br>Chadwick, Thomas; Newcastle University, Population Health Sciences Institute<br>Johnson, Sally; Newcastle Upon Tyne Hospitals NHS Foundation Trust, Great North Children's Hospital ; Newcastle University, National Renal Complement Therapeutics Centre<br>Kavanagh, David; Newcastle University, Translational and Clinical Research Institute ; Newcastle University, National Renal Complement Therapeutics Centre<br>Lecouturier, Jan; Newcastle University, Population Health Sciences Institute<br>Malina, Michal; Newcastle Upon Tyne Hospitals NHS Foundation Trust, Great North Children's Hospital; Newcastle University, National Renal Complement Therapeutics Centre<br>Moloney, Eoin; Optimax Access LTD, Health Economics<br>Oluboyede , Yemi; Newcastle University, Population Health Sciences Institute<br>Weetman, Christopher; Newcastle University, Newcastle Clinical Trials Unit<br>Wong, Edwin; Newcastle University, Translational and Clinical Research Institute ; Newcastle University, National Renal Complement Therapeutics Centre<br>Woodward, Len; aHUS alliance Global Action<br>Sheerin, Neil; Newcastle University, Translational and Clinical Research Institute ; Newcastle University, National Renal Complement Therapeutics Centre

Keywords:	NEPHROLOGY, HEALTH ECONOMICS, QUALITATIVE RESEARCH

Protocol for a multicentre, open label, prospective, single arm study of the safety and impact of eculizumab withdrawal in patients with atypical haemolytic uraemic syndrome

Version 1.0, 14.06.2021

Details of corresponding author

Full name	Postal address	Email	ORCHID ID
Ms Sarah Dunn, MSc	Newcastle Clinical Trials Unit, Newcastle University, 1-4 Claremont Terrace, Newcastle Upon Tyne, England, NE2 4AE	Sarah.dunn2@newcastle.ac.uk	0000-0002-1901-3931

Details of Co-authors

Full name	Department	Institution	City	Country	Email
Dr Victoria Brocklebank, MBBS	National Renal Complement Therapeutics Centre	Newcastle University	Newcastle Upon Tyne	England	Victoria.Brocklebank@newcastle.ac.uk
Mr Andrew Bryant, MSc	Population Health Sciences	Newcastle University	Newcastle Upon Tyne	England	andy.bryant@newcastle.ac.uk
Dr Sonya Carnell PhD	Newcastle Clinical Trials Unit	Newcastle University	Newcastle Upon Tyne	England	sonya.carnell@newcastle.ac.uk
Dr Thomas J Chadwick PhD	Population Health Sciences	Newcastle University	Newcastle Upon Tyne	England	thomas.chadwick@newcastle.ac.uk
Dr Sally Johnson MB ChB MRCPCH, PhD	Great North Children's Hospital, Translational and Clinical Research Institute and National Renal Complement Therapeutics Centre	Newcastle Upon Tyne Hospitals NHS Foundation Trust and Newcastle University	Newcastle Upon Tyne	England	sally.johnson15@nhs.net
Professor David Kavanagh, MB, ChB, MRCP, PhD, FRCP	Translational and Clinical Research Institute and National Renal Complement Therapeutics Centre	Newcastle University	Newcastle Upon Tyne	England	David.Kavanagh@newcastle.ac.uk

For peer review only - http://bmjopen.bmj.com/site/about/guidelines.xhtml

Ms Jan Lecouturier, MSc	Population Health Sciences	Newcastle University	Newcastle Upon Tyne	England	jan.lecouturier@newcastle.ac.uk
Dr Michal Malina, MD, PhD, FRCPCH	Great North Children's Hospital and National Renal Complement Therapeutics Centre	Newcastle Upon Tyne Hospitals NHS Foundation Trust And Newcastle University	Newcastle Upon Tyne	England	michal.malina@nhs.net
Mr Eoin Moloney, MSc	Health Economics	Optimax Access LTD	Newcastle Upon Tyne	England	eoin.moloney@optimaxaccess.com
Dr Yemi Oluboyede PhD	Population Health Sciences	Newcastle University	Newcastle Upon Tyne	England	yemi.oluboyede@newcastle.ac.uk
Mr Christopher Weetman, BSc	Newcastle Clinical Trials Unit	Newcastle University	Newcastle Upon Tyne	England	christopher.weetman@newcastle.ac.uk
Dr Edwin Kwan Soon Wong, MBChB, PhD MRCP	Translational and Clinical Research Institute and National Renal Complement Therapeutics Centre	Newcastle University	Newcastle Upon Tyne	England	edwin.wong@newcastle.ac.uk
Mr Len Woodward	N/A	aHUS alliance Global Action	Newcastle Upon Tyne	England	len.woodward@ntlworld.com
Professor Neil Sheerin, MBBS, PhD, MRCP	Translational and Clinical Research Institute and National Renal Complement Therapeutics Centre	Newcastle University	Newcastle Upon Tyne	England	Neil.Sheerin@newcastle.ac.uk

Word Count: 4504

For peer review only - http://bmjopen.bmj.com/site/about/guidelines.xhtml

ABSTRACT

Introduction

Atypical haemolytic uraemic syndrome (aHUS) is a rare, life threatening disease caused by excessive activation of part of the immune system called complement. Eculizumab is an effective treatment, controlling aHUS in 90% of patients. Due to the risk of relapse, lifelong treatment is currently recommended. Eculizumab treatment is not without problems, foremost being the risk of severe meningococcal infection, the burden of bi-weekly intravenous injections and the high cost.

This paper describes the design of the Stopping Eculizumab Treatment Safely in aHUS (SETS aHUS) trial that aims to establish whether a safety monitoring protocol, including the reintroduction of Eculizumab for those who relapse, could be a safe, alternative treatment strategy for patients with aHUS.

Methods and analysis

This is a multi-centre, non-randomised, open label study of Eculizumab withdrawal with continuous monitoring of serious adverse events using the Bayes factor single arm design. 30 patients will be recruited to withdraw from Eculizumab and have regular blood and urine tests for 24 months, to monitor for disease activity. If relapse occurs, treatment will be restarted within 24 hours of presentation. 20 patients will remain on treatment and complete health economic questionnaires only. An embedded qualitative study will explore the views of participants.

Ethics and dissemination

A favourable ethical opinion was obtained from the North East - Tyne & Wear South Research Ethics Committee. Outcomes will be disseminated via peer-reviewed articles and conference presentations.

Trial registration

EudraCT Number: 2017-003916-37

ISRCTN number: ISRCTN17503205

Date of Registration: 20 April 2018

Keywords

atypical Haemolytic Uraemic Syndrome, qualitative research, Complement, Eculizumab

Article summary

Strengths and limitations of this study

- This is the first UK trial to evaluate the safety of eculizumab withdrawal in patient with aHUS.
- This trial fulfils the NICE recommendation that a research programme, with robust methods, should be carried out to evaluate when stopping Eculizumab treatment or dose adjustment might occur.
- Clinical experience suggests if relapse occurs this will likely happen in the first 12 months of withdrawal however, this trial follows patients up for 24 months to capture those patients who may relapse after the 12-month point.
- The small number of aHUS patients on treatment in the UK is insufficient to conduct a standard parallel group, randomised controlled trial.
- COVID-19 has had an impact on recruitment.

Trial Status

This manuscript is based upon trial protocol version 7.0 dated 14th January 2021. Planned recruitment end date is 31st August 2021 and planned last patient visit is August 2023.

For peer review only - http://bmjopen.bmj.com/site/about/guidelines.xhtml

For peer review only

INTRODUCTION

Atypical Haemolytic Uraemic Syndrome (aHUS) is a severe, life-threatening disease characterised by thrombocytopenia, microangiopathic haemolytic anaemia and Acute Kidney Injury (AKI), and other organ involvement. Historically it is associated with a poor prognosis, with 50% of patients developing end stage kidney disease or dying in the first year after presentation [1] and a high risk of disease recurrence after kidney transplantation [2]. Prior to 2011, treatment options were limited and relied on plasma infusion or exchange, but in many cases this treatment failed to influence the course of disease [1] and was itself associated with significant morbidity and mortality [3]. In the UK, the incidence of aHUS is 0.4-0.5 cases per million per year [4].

The complement system is part of the innate immune system and in health is tightly regulated to prevent excessive activation. In 60-70% of patients with aHUS, a genetic variant or autoantibody increasing complement activation can be identified (10). In these patients, excessive activation of complement leads to endothelial injury and thrombus formation. The underlying genetic variant that predisposes to disease has an influence on the severity of disease and the likelihood of recurrent disease developing after transplantation [5].

Eculizumab is a humanized monoclonal antibody that inhibits the function of C5, an important protein involved in complement activation. Two uncontrolled, open label trials involving 36 adult and adolescent patients demonstrated the efficacy of Eculizumab treatment for aHUS over a 26 week period [6]. Additional prospective studies in children [7] and adults [8] confirmed efficacy. Follow-up of the original cohort suggests that treatment for 2 years is associated with good, longer-term clinical outcomes [9]. On the basis of the initial trial results, Eculizumab was approved for the treatment of aHUS by the European Medicines Agency (EMA) [10] and U.S. Food & Drug Administration (FDA) [11] in 2011. The National Institute for Health and Care Excellence (NICE) published its evaluation in 2015, recommending that Eculizumab should be used for the treatment of aHUS [12]. A recommendation in the NICE evaluation was that funding was on the condition that there was a ‘research programme with robust methods to evaluate when stopping treatment or dose adjustment might occur’.

Complement is part of the immune system, therefore, treatment with Eculizumab is immunosuppressive, in particular, increasing the risk of *Neisseria meningitidis* infection (1-2000 fold)[13, 14]. All patients are vaccinated against meningococcal infection before starting Eculizumab and in the UK continuous prophylactic antibiotics are recommended. Despite these recommendations, there have been 6 cases of meningococcal infection in the UK in patients on Eculizumab treatment for aHUS. We are also aware of uncommon infections occurring in this group (enteroviral pneumonitis and *Herpes simplex* meningitis) but whether these are attributable to Eculizumab treatment is unclear.

Although there have been reports of patients relapsing after the withdrawal of treatment [15], there is increasing evidence that continuous treatment is not required for all patients. From experience prior to the introduction of Eculizumab, the risk of relapse is greatest in the period immediately after first presentation. In the first year after presentation, 25% of children and 29% of adults will experience a relapse. 82% of relapses in adults, and 57% of relapses in children, occur in the first year after disease onset. Beyond the first year, only a further 20% of patients will relapse in the subsequent 5-10 years [1]. Therefore, a proportion of patients will not relapse after their initial presentation and will be on Eculizumab unnecessarily. In addition, with monitoring for relapse and early reintroduction of treatment, complications from relapse can be avoided [16-18].

In this trial we will test the safety of eculizumab withdrawal using a Bayesian trial design. The efficacy of self-monitoring will also be tested, and we will explore patients’ and parents/legal guardians’ views on how treatment and monitoring of disease can be delivered most effectively.

For peer review only - http://bmjopen.bmj.com/site/about/guidelines.xhtml

METHODS AND ANALYSIS

Objectives and outcome measures

Primary

The primary clinical objective is to determine the safety of Eculizumab withdrawal in patients with aHUS, measured by the number of patients with a Thrombotic microangiopathy (TMA) related Serious Adverse Event (SAE) defined as any of the following:

- Irreversible (>3 months) reduction in estimated glomerular filtration rate (eGFR) ~~by~~ not attributable to another cause:

In adults

- by ≥20% if the screening eGFR is <90mls/min/1.73m2,
- by >20% to a level <90mls/min/1.73m2 if the screening eGFR is >90mls/min/1.73m2.

In children

- by ≥20% if the screening eGFR is <75mls/min/1.73m2,
- by >20% to a level <75mls/min/1.73m2 if the screening eGFR is >75mls/min/1.73m2.

Secondary

Clinical

1. Measure the effectiveness of a monitoring protocol to detect disease relapse following withdrawal of Eculizumab.
2. Describe the relapse rate after withdrawal of Eculizumab.
3. Estimate the proportion of patients, currently on long-term treatment with Eculizumab, who can be maintained off treatment.
4. Describe the period from withdrawal to relapse in those patients who restart treatment.
5. Measure the change in estimated Glomerular Filtration rate (eGFR) over the course of the study.
6. Identify important clinical and laboratory indicators of imminent relapse.

Health Economic

7. To assess the within-trial costs and health outcomes (measured in terms of resource use of primary and secondary health care NHS services and quality-adjusted life years (QALYs)) for patients on standard care (not withdrawing from Eculizumab treatment) over the two-year trial duration.
8. To assess the within-trial costs and health outcomes for patients fully, or partially, withdrawing from Eculizumab treatment, and on a policy of protocolised monitoring, over the two-year trial duration.
9. To model the lifetime costs and outcomes associated with Eculizumab withdrawal, and a policy of protocolised monitoring following withdrawal (and treatment re-introduction if necessary), compared with standard care, beyond the two-year timeframe of the trial.

Trial Design

This is a multi-centre, non-randomised, open label study of Eculizumab withdrawal with continuous monitoring of serious adverse events using the Bayes factor single arm design of Johnson and Cook [19]. The patients will self-select whether they wish to withdraw from Eculizumab and carry out the monitoring protocol or remain on treatment and be part of the Health Economic analysis-only. An economic analysis, informed by the results of this trial, will determine whether Eculizumab withdrawal, substituting treatment with a protocolised surveillance and treatment reintroduction strategy, is cost-effective. The patient visit schedule for the withdrawal cohort is shown in Figure 1, and the Health Economic cohort in Figure 2.

For peer review only - http://bmjopen.bmj.com/site/about/guidelines.xhtml

Trial setting

This multi-centre trial will be carried out in up to 20 adult and paediatric Renal units (secondary and tertiary care) in the UK who are using Eculizumab to treat patients with aHUS.

Eligibility

All patients must fulfil the following inclusion criteria in order to be eligible for the trial:

- Age ≥2+ years of age,
- On Eculizumab treatment for at least 6 months,
- In remission with no evidence of ongoing microangiopathic haemolytic anaemia (MAHA) activity at screening defined by:
  - Platelet count > lower limit of normal as determined by local reference range,
  - Lactate Dehydrogenase (LDH) <x2 upper limit of normal as determined by local lab reference ranges,
- Normal renal function or Chronic Kidney Disease (CKD) stages 1-3,
- Absence of decline of renal function confirmed by review of available assessments of renal function for the preceding 6 months by the Chief Investigator (CI) and clinical members of the Trial Management Group (TMG).

The following inclusion criteria must be met only by those wishing to participate in the withdrawal component of the trial:

- Willing to attend for safety monitoring assessments,
- Willing to travel only to countries that can supply Eculizumab (to be confirmed with co-ordinating centre prior to travel),
- Able to perform or parent/guardian to perform and record self-monitoring urinalysis,
- Sexually active female patients must have a negative pregnancy test at screening and be using an effective contraception for the duration of the study.

OR

- fulfil one of the following criteria:
  - Be post-menopausal,
  - Have undergone surgical sterilisation.

The following exclusion criteria is applicable to all patients wishing to participate in the trial:

- Severe non-renal disease manifestations at initial presentation with aHUS, which in the opinion of the Chief Investigator and/or the clinical members of the TMG makes the risk of treatment withdrawal unacceptable,
- Current or planned pregnancy within the study duration,
- Unable to give informed consent or assent, or unable to obtain parent/guardian consent if under 16 years of age,
- Current participation in another clinical trial (not including participation in aHUS registries),
- Severe, uncontrolled hypertension (systolic blood pressure >160 mmHg) that is likely to induce at TMA.

The following exclusion criteria is applicable only to those wishing to participate in the withdrawal component of the trial:

- Loss of a previous transplant kidney to recurrent aHUS,
- Transplant recipient with a pathogenic mutation in *C3*, *CFH* or *CFB,*
- Haematuria rating of 3+.

Screening and Recruitment

30 patients will be recruited to withdraw from Eculizumab treatment, and 20 patients will be recruited who will remain on treatment and complete the Health Economic questionnaires only.

Patients with a diagnosis of aHUS receiving Eculizumab to treat disease in native or transplanted kidneys will be identified by the National aHUS Service, which maintains a list of patients who fulfil these criteria as part of the NHS

For peer review only

England commissioned service. Those patients who meet the genetic eligibility criteria will be highlighted to site teams who will carry out formal screening assessments. A physical examination and vital signs will be performed, and routine safety laboratory tests will be reviewed to ensure that a patient fulfils all eligibility criteria for entry into the study. Female participants withdrawing from treatment, who are of childbearing age and sexually active, will be required to have a negative pregnancy test prior to treatment withdrawal. Participants will also consent to have samples taken for exploratory analysis and storage at Newcastle University biobank for use in future research.

Consent will be sought from the parents/legal guardian on behalf of patients under the age of 16. Assent will be taken from those patients under 16 years old, as appropriate. No trial related procedures will be carried out prior to consent.

Intervention

Patients who consent to withdraw from Eculizumab will receive their last dose of Eculizumab at this visit (day -14).

Visit Details and Assessments

Baseline Assessments & Data collection for withdrawal cohort (Visit 2, Day 0 +/- 2 days)

Study day 0 will be the day that the participants would usually receive their next dose of Eculizumab, based on standard dosing schedules (+/-2 days). The Eculizumab will not be administered however, meningococcal prophylaxis will be continued for a further 2 weeks after day 0.

At day 0 of the study (Visit 2), participants will undergo the following assessments:
Vital signs (temperature, pulse and blood pressure), Height & weight, renal function (creatinine and eGFR), Urinalysis and urine protein/creatinine ratio, haemolysis markers including platelet count, haemoglobin, LDH, Electrolyte Profile, Liver Function (Bilirubin, ALT/AST, ALP, LDH, serum calcium, phosphate, albumin & total protein), haptoglobin (if available) and blood film, concomitant medication review, health-related quality of life questionnaires (EQ-5D-5L and SF-36) and health care utilisation questionnaire. The biomarkers and complement activation sample is also taken and stored at site before transfer to Newcastle University.

Study Visit Assessments & Data collection for withdrawal cohort (Visits 3-34)

Participants will be assessed regularly for evidence of disease relapse for the 2-year duration of the study. The participants will attend a total of 32 safety monitoring visits over the 2-year withdrawal follow-up period.

Trial participants will be reviewed at the trial site weekly (+/- 2 days) for the first month, then alternate weeks (+/-2 days) until month 6, then monthly (+/- 7 days) thereafter until the end of the trial period (month 24). At each study visit, the participants will undergo the monitoring assessments as detailed in Figure 1.
Paediatric participants must have their weight recorded at every visit for calculation of eGFR. At the end of the trial, the level of safety monitoring for those patients who remain off treatment and disease free will be decided by their local clinical care team in discussion with the National aHUS Service.

Due to the COVID-19 pandemic, participants may be unable to attend their scheduled follow up visits or may be attending a local hospital to have safety bloods taken. If the participants are unable to attend site due to self-isolation or underlying health issues; where possible, a remote, follow-up call will be carried out by a member of the local research team. Participants will be asked to report changes to their concomitant medications, any adverse events experienced since their previous follow-up and the results of their home urinalysis tests.

Health Economic Assessments

Participants, or their parent/legal guardian, in both withdrawal and Health Economics cohorts will complete the EQ-5D-5L (proxy version if patient < 12 years), SF36 (parent/legal guardian completes if patient <14 years) and a health care utilisation questionnaire at 8 timepoints. A time and travel questionnaire is completed at 1 timepoint, as detailed in Figures 1 and 2.

For peer review only - http://bmjopen.bmj.com/site/about/guidelines.xhtml

	Central Pre-Screen	Site Screen and Consent	Final Infusion	Withdrawal Phase																																	
**Month**	0	0	0	1					2		3		4		5		6		7	8	9	10	11	12	13	14	15	16	17	18	19	20	21	22	23	24	Unscheduled Visit
**Visit Number**	N/A	N/A	1	2	3	4	5	6	7	8	9	10	11	12	13	14	15	16	17	18	19	20	21	22	23	24	25	26	27	28	29	30	31	32	33	34	
Genetic Eligibility	X																																				
Medical History Review		X																																			
Informed consent		X																																			
Eligibility Checklist Completion		X																																			
Physical Examination		X																						X												X	X
Height & Weight		X																																			
Pregnancy test		X																																			
Eculizumab Infusion			X																																		
meningococcal prophylaxis			X	X																																	
urine analysis training				X																																	
Vital Signs		X		X	X	X	X	X	X	X	X	X	X	X	X	X	X	X	X	X	X	X	X	X	X	X	X	X	X	X	X	X	X	X	X	X	X
Concomitant medication Review		X		X	X	X	X	X	X	X	X	X	X	X	X	X	X	X	X	X	X	X	X	X	X	X	X	X	X	X	X	X	X	X	X	X	X
Renal Function (Creatinine & GFR)		X		X	X	X	X	X	X	X	X	X	X	X	X	X	X	X	X	X	X	X	X	X	X	X	X	X	X	X	X	X	X	X	X	X	X
Liver Function Tests		X		X	X	X	X	X	X	X	X	X	X	X	X	X	X	X	X	X	X	X	X	X	X	X	X	X	X	X	X	X	X	X	X	X	X
Haemolysis markers (full blood count & LDH)		X		X	X	X	X	X	X	X	X	X	X	X	X	X	X	X	X	X	X	X	X	X	X	X	X	X	X	X	X	X	X	X	X	X	X
Electrolyte profile (U&Es)		X		X	X	X	X	X	X	X	X	X	X	X	X	X	X	X	X	X	X	X	X	X	X	X	X	X	X	X	X	X	X	X	X	X	X
Haptoglobin & Blood film		X		X				X				X						X						X						X						X	X
Urine PCR		X		X				X				X						X						X						X						X	X
Biomarkers and complement activation sample		X		X	X	X		X		X		X						X			X			X						X						X	X
Home Urinalysis		X		Daily					3 times per week																												

For peer review only - http://bmjopen.bmj.com/site/about/guidelines.xhtml

Home Urinalysis diary review					x	x	x	x	x	x	x	x	x	x	x	x	x	x	x	x	x	x	x	x	x	x	x	x	x	x	x	x	x	x	x	x	
Adverse Events					x	x	x	x	x	x	x	x	x	x	x	x	x	x	x	x	x	x	x	x	x	x	x	x	x	x	x	x	x	x	x	x	x
EQ5D & SF36				x		x					x						x				x			x						x						x	
Health care Utilisation questionnaire				x		x					x						x				x			x						x						x	
Time & Travel Questionnaire																														x							

Figure 1. Data Collection Time Points for Withdrawal cohort

Follow - up								
Month	1		3	6	9	12	18	24
visit number	1	2	3	4	5	6	7	8
Medical History Review	X							
Informed consent	X							
Eligibility Checklist	X							
EQ5D & SF36	X	X	X	X	X	X	X	X
Health care Utilisation questionnaire	X	X	X	X	X	X	X	X
Time & Travel Questionnaire							X	

Figure 2. Data Collection Time Points for Health Economics Cohort

For peer review only - http://bmjopen.bmj.com/site/about/guidelines.xhtml

Self-monitored Urinalysis

Withdrawal participants, or their parent/legal guardian, will be trained to perform and understand the results of home urinalysis. Urinalysis will then be performed daily by the participant or parent/legal guardian for the first month and then three times per week for the remainder of the study period. The results will be recorded in a participant diary and will be reviewed at each study visit. Participants or their parent/legal guardian will report any significant change in urinalysis, not related to menstruation, using their own baseline result to guide them in relation to the thresholds as detailed in *Table 1*.

Table 1. Home Urinalysis result thresholds

Baseline	urinalysis result threshold (not related to menstruation)
Neg/Trace	++ on any occasion OR + on any two occasions 24 hours apart
+	+++ on any occasion OR ++ on any two occasions 24 hours apart
++	+++ on any occasion

If the threshold criteria are met, participants or their parent/legal guardian will contact their treatment site immediately to arrange an unscheduled visit to assess disease activity as outlined in *Figure 1*.

Trial Withdrawal

Participants will have the right to withdraw from the study at any time for any reason, and without giving a reason. The investigator will also have the right to withdraw participants from the study if she/he judges this to be in their best interest. Those participants who have been withdrawn from treatment can request to restart treatment, even if they have not relapsed. Data and blood samples provided by the participant up until the point of withdrawal will be included in analysis, unless they specifically request to have this removed. Participants who withdraw from the trial will not be replaced.

Change in Health Status

Participants will be advised to report any significant change in health status to the responsible site or local health care provider. Participants will be provided with a participant identification card to present to attending medical staff with details of the study, tests required and study centre and National aHUS Service contact details. Sites will notify the participants' General Practitioner of their involvement in the study and inform them of the required action to be taken in the case of suspected relapse. Criteria for a diagnosis of aHUS relapse are shown in *Figure 3*.

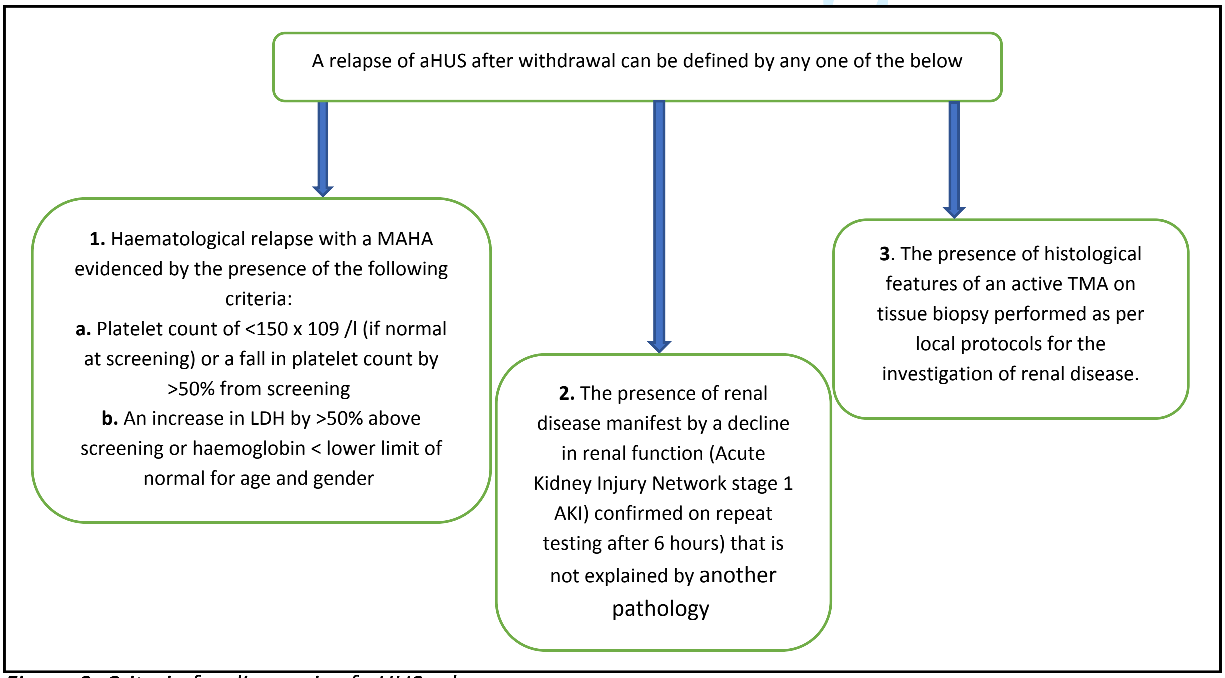

Figure 3. Criteria for diagnosis of aHUS relapse

For peer review only - http://bmjopen.bmj.com/site/about/guidelines.xhtml

If there is a clinical suspicion of disease activity, formal assessment will occur as outlined in *Figure 1*, Unscheduled visit column.

Any other adverse events that could represent a relapse will be discussed with the Investigators and/or the aHUS National Service. A decision to restart will be made according to current Service procedures.

Relapse Management

When a relapse is diagnosed, participants will restart Eculizumab treatment within 24 hours of presentation provided there is no evidence of an active infection that would be a contra-indication to treatment at the recommended dose of 900mg weekly for the first 4 weeks then 1200mg every two weeks thereafter (or age adjusted dose and regime). TMA activity will be monitored (platelet count, LDH) as recommended by attending clinician until haematological remission is achieved as defined in *Figure 4*.

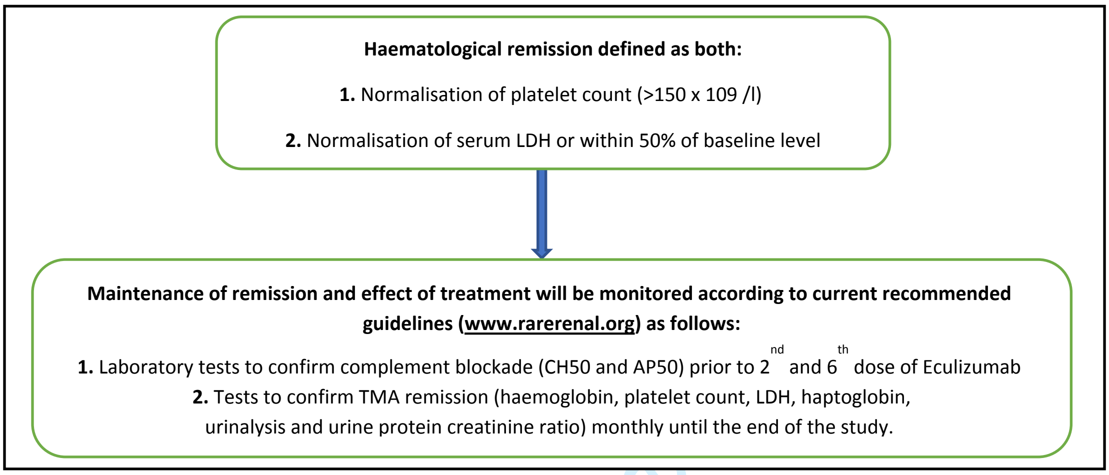

Figure 4. Haematological remission

Participants who relapse and require re-introduction of Eculizumab treatment will remain on treatment in study under follow up for the full 2 years of the study. Home urinalysis will not be required after re-introduction of Eculizumab treatment.

Participants will consent to travel to only those countries where Eculizumab is available. If a participant relapses while they are travelling outside of the country, the National aHUS Service will make arrangements with the destination country to access and fund Eculizumab if required, with arrangements from the commissioning authority.

Embedded qualitative study

In-depth one-to-one telephone interviews will be conducted following a topic guide developed with the input of the research team, including PPI. The intention is to keep interviews very broad to ensure we capture the full experience of interviewees.

Up to 30 patients who withdraw from Eculizumab and up to 20 patients who decline to withdraw will be approached to participate. Up to 20 patients who withdraw will be re-interviewed at the end of the withdrawal period (24 months later) to explore their views of the monitoring protocol. This group will be asked at the first interview if they agree to be contacted again towards the end of the study for a follow up interview. Where possible, any patients who relapse and go back onto treatment will also be interviewed. Consent will be recorded at the time of the interview. Interviews will be digitally recorded with the permission of the interviewee, transcribed verbatim and anonymised.

For peer review only - http://bmjopen.bmj.com/site/about/guidelines.xhtml

Safety reporting

All Adverse Events (AEs) occurring from the point of withdrawal (day 0) to end of study participation will be recorded. SAEs occurring from the point of withdrawal (day 0) must be reported to The Newcastle Clinical Trials Unit (NCTU) within 24 hours of the site becoming aware of the event. Serious Adverse Reactions (SARs) and Suspected Unexpected Serious Adverse Reactions (SUSARs) are reportable only for those participants who have Eculizumab treatment re-initiated during their participation in the trial. The assessment of expectedness will be performed by the PI at site against the approved Reference Safety Information (RSI) for the trial (Section 4.8 of the Soliris SmPC).

Statistical Analysis

Sample size

A maximum of 30 patients will be recruited to the withdrawal component; this is judged to be reasonable given the rare nature of the disease. The specifics of this sample size are intrinsically linked to the Bayes factor single arm binary design employed to analyse the primary outcome measure. There is no allowance for loss to follow-up as this patient group is already subject to a high degree of clinical follow-up and death is defined as one of the serious events under consideration. 20 patients will be recruited to the non-withdrawal arm as a comparator group for health economic analysis only.

Analysis

This is a single arm, open label trial with the primary endpoint being a binary response (the presence/absence of a primary outcome event within the follow-up period). We will compare the rate of serious events following the withdrawal of medication to that expected under standard care.

The Bayes factor single arm binary model [19] will be used to monitor the trial. Based on historical data, the event rate for the standard of care is 0.06, and we expect that withdrawal of the treatment would give a rate of 0.12. This choice of rate has been informed in discussion with patients. Using this Bayesian hypothesis test-based design, we assume the rate is 0.06 under the null, and 0.12 under the alternative hypothesis.

We assume that the sample distribution of number of responses follows a binomial distribution and use an inverse moment prior for response under the alternative hypothesis.

Stopping Rules

A minimum of 5 patients will be enrolled before applying the stopping rules, and the cohort size for monitoring is 5 patients. The Data Monitoring Committee (DMC) can request earlier review if adverse events are reported before this point.

We implement two stopping rules:

(1) We will stop the trial for superiority (there being fewer serious events on the intervention than would be expected under standard of care) if the posterior probability of the alternative hypothesis is less than 0.05, i.e. $Pr(H1|Data) < 0.05$;

(2) We will stop the trial for inferiority if the posterior probability of the alternative hypothesis is greater than 0.80, i.e., $Pr(H1|Data) > 0.80$.

Operating Characteristics and Stopping Boundaries

The operating characteristics (Table 2) and stopping boundaries (Table 3) were produced using the M. D. Anderson Cancer Center Department of Biostatistics software BayesFactorBinary, version 1.0

https://biostatistics.mdanderson.org/SoftwareDownload/SoftwareFiles/BayesFactorBinary/UsersGuide_BayesFactorBinary.pdf

For peer review only

For peer review only - http://bmjopen.bmj.com/site/about/guidelines.xhtml

Table 2. Operating Characteristics

Scenario	True rate of serious events	Probability of Stopping for Inferiority	Probability of Stopping for Superiority	Average number of patients treated (Percentiles: 10%, 25%, 50%, 75%, 90%)
1	0.06	0.096	0	28.44 (30, 30, 30, 30, 30)
2	0.12	0.443	0	23.48 (5, 15, 30, 30, 30)
3	0.18	0.753	0	17.76 (5, 10, 15, 30, 30)
4	0.24	0.928	0	13.72 (5, 5, 15, 15, 30)
5	0.30	0.982	0	10.52 (5, 5, 10, 15, 20)

If the true rate is 0.06 (Scenario 1, null hypothesis), the trial will stop with probabilities of 0.096 and 0 in favour of the alternative and null hypotheses, respectively. The average number of patients (10%, 90% percentiles) is 28.44 (30, 30). If the true rate is 0.12 (Scenario 2, alternative hypothesis), the trial will stop with probabilities of 0.443 and 0 in favour of the alternative and null hypotheses, respectively. The average number of patients (10%, 90%) is 23.48 (5, 30).

Table 3 – SETS aHUS Trial Stopping Boundaries

Number of patients (in complete cohorts of 5)	**Stop** the trial for Superiority if there are this many **Events** (inclusive)	**Continue** the trial if there are this many **Events** (inclusive)	**Stop** the trial for Inferiority if there are this many **Events** (inclusive)
5	Never stop for superiority with this many patients	0-1	2-5
10 or 15	Never stop for superiority with this many patients	0-2	3-15
20	Never stop for superiority with this many patients	0-3	4-20
25 or 30	Never stop for superiority with this many patients	0-4 (The trial always stops at 30 patients, which is the maximum)	5-30

The study will stop for inferiority with 2 serious events in the first cohort of 5 participants. Subsequently, the study would stop if 3 or more serious events are observed in the first 15 participants, 4 or more in the first 20 participants, and 5 or more in the whole study population. We are well placed to respond to any negative safety signal.

1000 repetitions were used in the software simulation. Calculations with different numbers of repetitions resulted in unchanged stopping boundaries with only marginal changes to the operating characteristics.

There may be differing risk of relapse according to disease aetiology. However, the available numbers do not allow for risk strata to be monitored separately. The DMC will consider this within their remit.

In addition to this ongoing analysis, at the end of the study, data will also be reported descriptively, together with the number of patients recruited. Descriptive statistics reported will be selected as appropriate to the specific outcome measure. For proportion outcomes, the number of patients recording the event will also be reported.

Due to the sample size, no comparative statistical methods will be applied. There will be no imputation of missing data and a complete case analysis will be undertaken.

Subgroup Analyses

Except for the analysis of the primary outcome on an ongoing basis, the analyses described above may be reported separately for different genetic groups or risk strata.

For peer review only - http://bmjopen.bmj.com/site/about/guidelines.xhtml

Health Economic analysis

Within-trial assessments of costs and outcomes

Costs and health outcomes (measured in terms of resource use of primary and secondary health care NHS services and QALYs) associated with Eculizumab withdrawal (30 participants), compared with standard care (20 participants), will be assessed over the 24-month follow-up period. Information on costs and health outcomes will be recorded for each individual involved in both treatment groups. Data derived from the within-trial analysis will be assessed to understand the key determinants of differences in costs and outcomes between the two patient groups. Data will then be used to parameterise the lifetime economic model (combined with data from the literature).

Assessment of cost-effectiveness

An economic decision model will be developed to assess the cost-effectiveness of the alternative treatment options under evaluation. Costs and health consequences, measured in terms of QALYs, associated with Eculizumab withdrawal, and a policy of monitoring following withdrawal, and standard care, beyond the two-year timeframe of the trial will be captured. We propose to conduct a cost-utility analysis, with results presented in terms of incremental cost per QALY gained.

Qualitative analysis

We will take an inductive approach to data collection and analysis. This means there is no a priori theory; themes, concepts and theories will be elicited from the interview data when it is analysed and drawing upon relevant literature, PPI, and experts in the study team. Data will be analysed thematically using a constant comparative method. This entails a process of familiarisation with the data and then the development of a thematic framework. A small number of transcripts will be coded, and the framework amended accordingly. A second level analysis will be conducted using a constant comparative method. This involves a process of comparing and contrasting themes elicited from the data, within and across interviews [21]. NVivo will be used as a data management tool.

Trial Management and monitoring

This trial is sponsored by the Newcastle Upon Tyne Hospitals NHS Foundation Trust. The trial will be co-ordinated by a TMG that will include those individuals responsible for the day-to-day management of the trial. A Trial Steering Committee (TSC) made up of independent clinical and lay members will provide overall supervision of the trial. A Data Monitoring Committee (DMC) composed of independent clinicians and statistician will undertake independent review and monitor efficacy and safety endpoints. The Newcastle Clinical Trials Unit (NCTU) will be responsible for communicating protocol amendments to participating sites and carrying out central, remote, and on-site monitoring.

Confidentiality and data handling

Personal data will be regarded as strictly confidential. To preserve anonymity, a unique participant ID will be assigned to each participant at consent. Only the clinical team at the participating sites will have access to key data which links study identifiers to individual datasets. All study records and Investigator Site Files will be kept at site in a locked filing cabinet with restricted access.

Written consent will be sought from participants or legal guardians, if patient is under the age of 16 years, to allow access to their hospital records.

Data is recorded by authorised staff and stored in a secure web-based electronic data capture system (MACRO) designed and maintained by NCTU hosted on secure servers at Rackspace within the UK. Analysis of the data will be undertaken by the Newcastle University trial statisticians. Anonymised data from this trial may be available to the scientific community subject to regulatory and ethics approval. Requests for data should be directed to the corresponding author. All study data will be archived for 5 years.

For peer review only - http://bmjopen.bmj.com/site/about/guidelines.xhtml

Patient and public involvement (PPI)

A PPI representative sits on the Trial Management Group, was involved in protocol and study document development, and is involved in ongoing trial management discussions. We also have an aHUS patient as an independent member of the Trial Steering Committee.

ETHICS AND DISSEMINATION

A favourable ethical opinion was obtained from the North East - Tyne & Wear South Research Ethics Committee in April 2018. Written informed consent will be obtained from all participants prior to their involvement in the trial. The results of the study will be submitted to peer-reviewed journals, presented at conferences and on the trial website.

DISCUSSION

This study will determine whether it is safe to withdraw Eculizumab using a trial methodology designed to detect an excess of adverse outcomes following withdrawal (primary endpoint). The study will also estimate the proportion of patients with aHUS that can be maintained off Eculizumab and test a system for surveillance to identify relapse early (secondary endpoints). This will allow a cost-utility analysis to be conducted, exploring the impact of treatment withdrawal [20]. This carefully monitored patient group will allow us to determine how early sub-clinical relapse can be detected using standard biochemical and haematological measurements and novel biomarkers of complement activation or tissue injury. An embedded qualitative study of patients, both those who withdraw and decide not to withdraw, will explore attitudes towards treatment and its withdrawal.

REFERENCES

1. Fremeaux-Bacchi V, Fakhouri F, Garnier A, Bienaime F, Dragon-Durey MA, Ngo S, Moulin B, Servais A, Provot F, Rostaing L *et al*: Genetics and outcome of atypical hemolytic uremic syndrome: a nationwide French series comparing children and adults. *Clin J Am Soc Nephrol* 2013, 8(4):554-562.
2. Noris M, Remuzzi G: Thrombotic microangiopathy after kidney transplantation. *Am J Transplant* 2010, 10(7):1517-1523.
3. Johnson S, Stojanovic J, Ariceta G, Bitzan M, Besbas N, Frieling M, Karpman D, Landau D, Langman C, Licht C *et al*: An audit analysis of a guideline for the investigation and initial therapy of diarrhea negative (atypical) hemolytic uremic syndrome. *Pediatric nephrology (Berlin, Germany)* 2014, 29(10):1967-1978.
4. Sheerin NS, Kavanagh D, Goodship TH, Johnson S: A national specialized service in England for atypical haemolytic uraemic syndrome-the first year's experience. *QJM* 2016, 109(1):27-33.
5. Le Quintrec M, Zuber J, Moulin B, Kamar N, Jablonski M, Lionet A, Chatelet V, Mousson C, Mourad G, Bridoux F *et al*: Complement genes strongly predict recurrence and graft outcome in adult renal transplant recipients with atypical hemolytic and uremic syndrome. *Am J Transplant* 2013, 13(3):663-675.
6. Legendre CM, Licht C, Muus P, Greenbaum LA, Babu S, Bedrosian C, Bingham C, Cohen DJ, Delmas Y, Douglas K *et al*: Terminal complement inhibitor eculizumab in atypical hemolytic-uremic syndrome. *The New England journal of medicine* 2013, 368(23):2169-2181.
7. Greenbaum LA, Fila M, Ardissino G, Al-Akash SI, Evans J, Henning P, Lieberman KV, Maringhini S, Pape L, Rees L *et al*: Eculizumab is a safe and effective treatment in pediatric patients with atypical hemolytic uremic syndrome. *Kidney international* 2016, 89(3):701-711.
8. Fakhouri F, Hourmant M, Campistol JM, Cataland SR, Espinosa M, Gaber AO, Menne J, Minetti EE, Provot F, Rondeau E *et al*: Terminal Complement Inhibitor Eculizumab in Adult Patients With Atypical Hemolytic Uremic Syndrome: A Single-Arm, Open-Label Trial. *Am J Kidney Dis* 2016, 68(1):84-93.

For peer review only

9. Licht C, Greenbaum LA, Muus P, Babu S, Bedrosian CL, Cohen DJ, Delmas Y, Douglas K, Furman RR, Gaber OA *et al*: Efficacy and safety of eculizumab in atypical hemolytic uremic syndrome from 2-year extensions of phase 2 studies. *Kidney international* 2015, 87(5):1061-1073.
10. European Medicines Agency: http://www.ema.europa.eu/docs/en_GB/document_library/EPAR_-_Assessment_Report_-_Variation/human/000791/WC500119185.pdf. 2011.
11. FDA: http://www.fda.gov/AboutFDA/CentersOffices/OfficeofMedicalProductsandTobacco/CDER/ucm273089.htm.
12. https://www.nice.org.uk/guidance/hst1/resources/eculizumab-for-treating-atypical-haemolytic-uraemic-syndrome-1394895848389
13. Struijk GH, Bouts AH, Rijkers GT, Kuin EA, ten Berge IJ, Bemelman FJ: Meningococcal sepsis complicating eculizumab treatment despite prior vaccination. *Am J Transplant* 2013, 13(3):819-820.
14. McNamara LA, Topaz N, Wang X, Hariri S, Fox L, MacNeil JR: High Risk for Invasive Meningococcal Disease Among Patients Receiving Eculizumab (Soliris) Despite Receipt of Meningococcal Vaccine. *MMWR Morb Mortal Wkly Rep* 2017, 66(27):734-737.
15. Carr R, Cataland SR: Relapse of aHUS after discontinuation of therapy with eculizumab in a patient with aHUS and factor H mutation. *Annals of hematology* 2013, 92(6):845-846.
16. Ardissino G, Testa S, Possenti I, Tel F, Paglialonga F, Salardi S, Tedeschi S, Belingheri M, Cugno M: Discontinuation of eculizumab maintenance treatment for atypical hemolytic uremic syndrome: a report of 10 cases. *Am J Kidney Dis* 2014, 64(4):633-637.
17. Wijnsma KL, Duineveld C, Volokhina EB, van den Heuvel LP, van de Kar N, Wetzels JFM: Safety and effectiveness of restrictive eculizumab treatment in atypical haemolytic uremic syndrome. *Nephrol Dial Transplant* 2018, 33(4):635-645.
18. Fakhouri F, Fila M, Hummel A, Ribes D, Sellier-Leclerc AL, Ville S, Pouteil-Noble C, Coindre JP, Le Quintrec M, Rondeau E *et al*: Eculizumab discontinuation in children and adults with atypical haemolytic uremic syndrome: a prospective multicentric study. *Blood* 2020.
19. Johnson VE, Cook JD: Bayesian design of single-arm phase II clinical trials with continuous monitoring. *Clin Trials* 2009, 6(3):217-226.
20. Drummond MF, O'Brien B, Stoddart GL, Torrance GW: Basic Types of Economic Evaluation. In: *Methods for economic evaluation of health care programmes.* edn.: Oxford University Press; 2005.
21. Glaser BG. The Constant Comparative Method of Qualitative Analysis. *Social Problems* 1965, 12(4):436-45.

For peer review only

For peer review only - http://bmjopen.bmj.com/site/about/guidelines.xhtml

Acknowledgements

The authors would like to thank Dr Colin Muirhead who was responsible for the statistical proposal in the initial grant application.

The authors would also like to thank all the Principal Investigators for their contribution and support, the research nurses, the members of the TSC and DMC and all the patients involved in the SETS aHUS Trial.

Author statement

Professor Neil Sheerin is the Chief Investigator and senior author of the SETS aHUS design and has led on grant acquisition and protocol development. Professor David Kavanagh, Dr Sally Johnson, Mr Len Woodward, Ms Jan Lecouturier, Dr Thomas Chadwick, and Dr Yemi Oluboyede are co-applicants of the grant and contributed to protocol development. Dr Sonya Carnell and Ms Sarah Dunn are part of the trial management team and contributed to protocol development. Mr Eoin Moloney is a Health Economist and contributed to protocol development. Mr Andy Bryant is a research statistician and contributed to protocol development. Mr Christopher Weetman is a data manager and contributed to protocol development.

This paper was drafted from the current approved version of the protocol; all authors commented and amended drafts of the paper. All authors read and approved the final manuscript.

Funding

The SETS aHUS Trial is funded by the National Institute for Health Research (NIHR) Health Technology Assessment (HTA) Programme (project number 15/130/94). The views expressed are those of the authors and not necessarily those of the NIHR or the Department of Health and Social Care.

Conflict of interests

SB has received honoraria for sitting on advisory boards for Alexion and Novartis. DK is a director of and scientific advisor to Gyroscope Therapeutics. DK received advisory board payments from Idorsia, Novartis, ChemoCentryx, Alexion, Apellis, Biomarin and Sarepta. DK's spouse works for GSK. MM has received honoraria for educational talks and honorarium for national lead of aHUS registry, both from Alexion and travel expenses from Alexion. EKSW has received honoraria for lectures and/or advisory boards for Alexion Pharmaceutical, Biocryst and Novartis. LW has received expenses, honoraria and fees for advisory board participation and talks from Alexion and Roche. NS has given lectures or sat on advisory boards for Alexion Pharmaceutical, Roche, Astra Zeneca and Novartis, no personal honoraria, all payments made to the department.

Patient and public involvement

Patients and/or the public were involved in the design, or conduct, or reporting, or dissemination plans of this research. Refer to the Methods section for further details.

Patient consent for publication

Not required.

Provenance and peer review

Not commissioned; externally peer reviewed during funding application process.

For peer review only

World Health Organisation Trial Registration Set

Primary registry and trial identifying number	ISRCTN<br>ISRCTN17503205
Date of registration in primary registry	20 April 2018
Secondary identifying numbers	EudraCT: 2017-003916-37
Source(s) of monetary or material support	NIHR HTA
Primary sponsor	Newcastle Upon Tyne Hospitals NHS FT – Christopher Price christoper.price6@nhs.uk
Secondary sponsor(s)	N/A
Contact for public queries	Trial Manager – Sarah Dunn sarah.dunn2@newcastle.ac.uk
Contact for scientific queries	Chief Investigator – Professor Neil Sheerin neil.sheerin@newcastle.ac.uk
Public title	Stopping Eculizumab Treatment Safely in aHUS (SETS aHUS)
Scientific title	Multicentre, open label, prospective, single arm study of safety impact of Eculizumab withdrawal
Countries of recruitment	England and Scotland
Health condition(s) or problem(s) studied	atypical Haemolytic Uraemic Syndrome
Intervention(s)	Withdrawal of Eculizumab
Key inclusion and exclusion criteria	Key inclusion criteria:<br>• Age ≥2+ years of age,<br>• On Eculizumab treatment for at least 6 months,<br>• In remission with no evidence of ongoing microangiopathic haemolytic anaemia (MAHA) activity at screening defined by:<br>- Platelet count > lower limit of normal as determined by local reference range,<br>- Lactate Dehydrogenase (LDH) <x2 upper limit of normal as determined by local lab reference ranges,<br>• Normal renal function or Chronic Kidney Disease (CKD) stages 1-3,<br>• Absence of decline of renal function confirmed by review of available assessments of renal function for the preceding 6 months by the Chief Investigator (CI) and clinical members of the Trial Management Group (TMG).<br>Key Exclusion Criteria:<br>• Severe non-renal disease manifestations at initial presentation with aHUS, which in the opinion of the Chief Investigator and/or the clinical members of the TMG makes the risk of treatment withdrawal unacceptable,<br>• Current or planned pregnancy within the study duration,<br>• Unable to give informed consent or assent, or unable to obtain parent/guardian consent if under 16 years of age,

	• Current participation in another clinical trial (not including participation in aHUS registries),<br>• Severe, uncontrolled hypertension (systolic blood pressure >160 mmHg) that is likely to induce at TMA.
Study type	Allocation: non-randomized<br>Masking: open label<br>Primary purpose: Safety<br>Phase IIb
Date of first enrolment	November 2018
Target sample size	50: 30 withdrawal and 20 non-withdrawal
Recruitment status	Recruiting
Primary outcome(s)	To determine the safety of Eculizumab withdrawal in patients with aHUS
Key secondary outcomes	1. Measure the effectiveness of a monitoring protocol to detect disease relapse following withdrawal of Eculizumab.<br>2. Describe the relapse rate after withdrawal of Eculizumab.<br>3. Estimate the proportion of patients, currently on long-term treatment with Eculizumab, who can be maintained off treatment.<br>4. Describe the period from withdrawal to relapse in those patients who restart treatment.<br>5. Measure the change in estimated Glomerular Filtration rate (GFR) over the course of the study.<br>6. Identify important clinical and laboratory indicators of imminent relapse.<br>7. To assess the costs and health outcomes (measured in terms of adverse events and quality-adjusted life years (QALYs)) for patients on standard care (not withdrawing from Eculizumab treatment) over the two-year trial duration.<br>8. To assess the costs and health outcomes for patients fully, or partially, withdrawing from Eculizumab treatment, and on a policy of protocolised monitoring, over the two-year trial duration.<br>*9.* To model the costs and health consequences (measured in terms of QALYs) associated with Eculizumab withdrawal, and a policy of protocolised monitoring following withdrawal (and treatment re-introduction if necessary), compared with standard care, beyond the two-year timeframe of the trial.

For peer review only - http://bmjopen.bmj.com/site/about/guidelines.xhtml

	Central Pre-Screen	Site Screen and Consent	Final Infusion	Withdrawal Phase																																
Month	0	0	0	1					2		3		4		5		6		7	8	9	10	11	12	13	14	15	16	17	18	19	20	21	22	23	24	Unscheduled Visit
Visit Number	N/A	N/A	1	2	3	4	5	6	7	8	9	10	11	12	13	14	15	16	17	18	19	20	21	22	23	24	25	26	27	28	29	30	31	32	33	34	
Genetic Eligibility	X																																				
Medical History Review		X																																			
Informed consent		X																																			
Eligibility Checklist Completion		X																																			
Physical Examination		X																								X											X	X
Height & Weight		X																																			
Pregnancy test		X																																			
Eculizumab Infusion			X																																		
meningococcal prophylaxis			X	X																																	
urine analysis training				X																																	
Vital Signs		X		X	X	X	X	X	X	X	X	X	X	X	X	X	X	X	X	X	X	X	X	X	X	X	X	X	X	X	X	X	X	X	X	X	X
Concomitant medication Review		X		X	X	X	X	X	X	X	X	X	X	X	X	X	X	X	X	X	X	X	X	X	X	X	X	X	X	X	X	X	X	X	X	X	X
Renal Function (Creatinine & GFR)		X		X	X	X	X	X	X	X	X	X	X	X	X	X	X	X	X	X	X	X	X	X	X	X	X	X	X	X	X	X	X	X	X	X	X
Liver Function Tests		X		X	X	X	X	X	X	X	X	X	X	X	X	X	X	X	X	X	X	X	X	X	X	X	X	X	X	X	X	X	X	X	X	X	X
Haemolysis markers (full blood count & LDH)		X		X	X	X	X	X	X	X	X	X	X	X	X	X	X	X	X	X	X	X	X	X	X	X	X	X	X	X	X	X	X	X	X	X	X
Electrolyte profile (U&Es)		X		X	X	X	X	X	X	X	X	X	X	X	X	X	X	X	X	X	X	X	X	X	X	X	X	X	X	X	X	X	X	X	X	X	X
Haptoglobin & Blood film		X		X				X				X						X						X						X						X	X
Urine PCR		X		X				X				X						X						X						X						X	X
Biomarkers and complement activation sample		X		X	X	X		X		X		X						X			X			X						X						X	X

SETS aHUS Protocol paper – figure 1 Data Collection Time Points for Withdrawal cohort – V1.0 29.04.2021

For peer review only - http://bmjopen.bmj.com/site/about/guidelines.xhtml

Home Urinalysis		X		Daily					3 times per week																												
Home Urinalysis diary review					x	x	x	x	x	x	x	x	x	x	x	x	x	x	x	x	x	x	x	x	x	x	x	x	x	x	x	x	x	x	x	x	
Adverse Events					x	x	x	x	x	x	x	x	x	x	x	x	x	x	x	x	x	x	x	x	x	x	x	x	x	x	x	x	x	x	x	x	x
EQ5D & SF36				x		x					x						x				x			x						x						x	
Health care Utilisation questionnaire				x		x					x						x				x			x						x						x	
Time & Travel Questionnaire																														x							

SETS aHUS Protocol paper – figure 1 Data Collection Time Points for Withdrawal cohort – V1.0 29.04.2021

For peer review only

	Follow - up							
month	1		3	6	9	12	18	24
visit number	1	2	3	4	5	6	7	8
Medical History Review	X							
Informed consent	X							
Eligibility Checklist	X							
EQ5D & SF36	X	X	X	X	X	X	X	X
Health care Utilisation questionnaire	X	X	X	X	X	X	X	X
Time & Travel Questionnaire							X	

For peer review only

SETS aHUS Protocol paper – Figure 2. Data Collection Time Points for Health Economics Cohort V1.0 29.04.2021

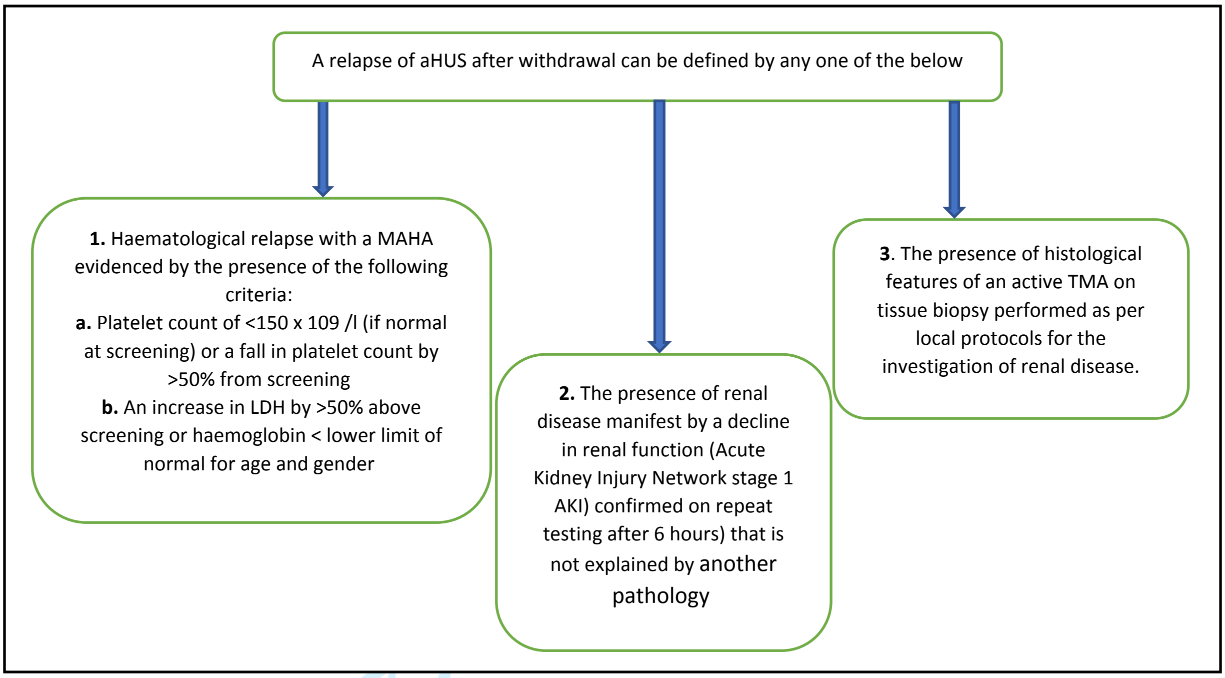

For peer review only

SETS aHUS Protocol paper – Figure 3. Criteria for diagnosis of aHUS relapse – V1.0 29.04.2021

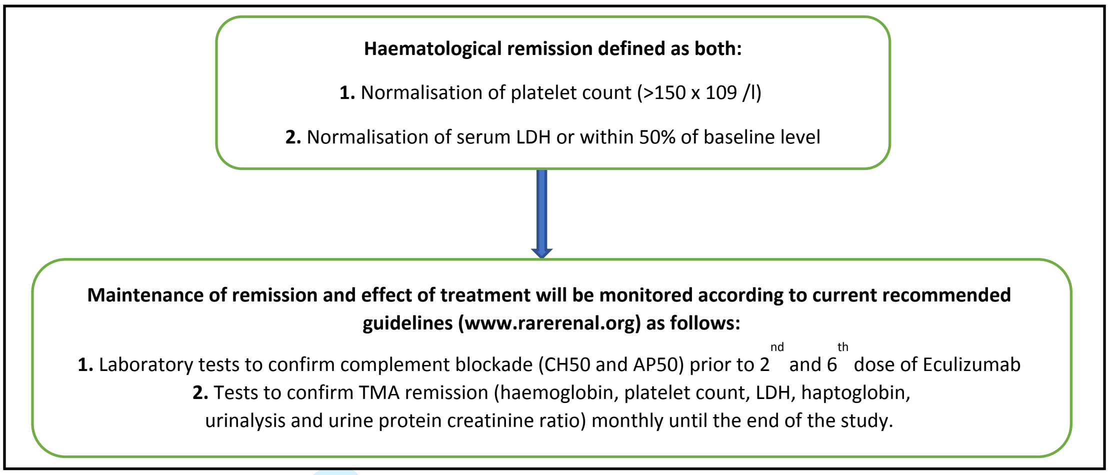

For peer review only

SETS aHUS Protocol paper – Figure 4. Haematological remission – V1.0 29.04.2021

Reporting checklist for protocol of a clinical trial.

Based on the SPIRIT guidelines.

Instructions to authors

Complete this checklist by entering the page numbers from your manuscript where readers will find each of the items listed below.

Your article may not currently address all the items on the checklist. Please modify your text to include the missing information. If you are certain that an item does not apply, please write "n/a" and provide a short explanation.

Upload your completed checklist as an extra file when you submit to a journal.

In your methods section, say that you used the SPIRITreporting guidelines, and cite them as:

Chan A-W, Tetzlaff JM, Gøtzsche PC, Altman DG, Mann H, Berlin J, Dickersin K, Hróbjartsson A, Schulz KF, Parulekar WR, Krleža-Jerić K, Laupacis A, Moher D. SPIRIT 2013 Explanation and Elaboration: Guidance for protocols of clinical trials. BMJ. 2013;346:e7586

		Reporting Item	Page Number
**Administrative information**			
Title	#1	Descriptive title identifying the study design, population, interventions, and, if applicable, trial acronym	1

For peer review only

Trial registration	#2a	Trial identifier and registry name. If not yet registered, name of intended registry	3
Trial registration: data set	#2b	All items from the World Health Organization Trial Registration Data Set	19
Protocol version	#3	Date and version identifier	3
Funding	#4	Sources and types of financial, material, and other support	16
Roles and responsibilities: contributorship	#5a	Names, affiliations, and roles of protocol contributors	1
Roles and responsibilities: sponsor contact information	#5b	Name and contact information for the trial sponsor	13 and 17
Roles and responsibilities: sponsor and funder	#5c	Role of study sponsor and funders, if any, in study design; collection, management, analysis, and interpretation of data; writing of the report; and the decision to submit the report for publication, including whether they will have	NA – sponsor and funder do not have involvement in these activities.

For peer review only

		ultimate authority over any of these activities	
Roles and responsibilities: committees	#5d	Composition, roles, and responsibilities of the coordinating centre, steering committee, endpoint adjudication committee, data management team, and other individuals or groups overseeing the trial, if applicable (see Item 21a for data monitoring committee)	11, 12 and 13
**Introduction**			
Background and rationale	#6a	Description of research question and justification for undertaking the trial, including summary of relevant studies (published and unpublished) examining benefits and harms for each intervention	4
Background and rationale: choice of comparators	#6b	Explanation for choice of comparators	n/a – the trial withdraws patients from their current medication, no comparator required
Objectives	#7	Specific objectives or hypotheses	4 and 5
Trial design	#8	Description of trial design including type of trial (eg, parallel	4

For peer review only

		group, crossover, factorial, single group), allocation ratio, and framework (eg, superiority, equivalence, non-inferiority, exploratory)	
**Methods: Participants, interventions, and outcomes**			
Study setting	#9	Description of study settings (eg, community clinic, academic hospital) and list of countries where data will be collected. Reference to where list of study sites can be obtained	6
Eligibility criteria	#10	Inclusion and exclusion criteria for participants. If applicable, eligibility criteria for study centres and individuals who will perform the interventions (eg, surgeons, psychotherapists)	6
Interventions: description	#11a	Interventions for each group with sufficient detail to allow replication, including how and when they will be administered	7

For peer review only

Interventions: modifications	#11b	Criteria for discontinuing or modifying allocated interventions for a given trial participant (eg, drug dose change in response to harms, participant request, or improving / worsening disease)	9
Interventions: adherence	#11c	Strategies to improve adherence to intervention protocols, and any procedures for monitoring adherence (eg, drug tablet return; laboratory tests)	9 – monitoring of home urinalysis
Interventions: concomitant care	#11d	Relevant concomitant care and interventions that are permitted or prohibited during the trial	7 – relating to meningococcal prophylaxis. No other concomitant medication requirements imposed by the trial
Outcomes	#12	Primary, secondary, and other outcomes, including the specific measurement variable (eg, systolic blood pressure), analysis metric (eg, change from baseline, final value, time to event), method of aggregation (eg, median, proportion), and time point for each outcome. Explanation of the clinical relevance of chosen	5

For peer review only

		efficacy and harm outcomes is strongly recommended	
Participant timeline	#13	Time schedule of enrolment, interventions (including any run-ins and washouts), assessments, and visits for participants. A schematic diagram is highly recommended (see Figure)	7
Sample size	#14	Estimated number of participants needed to achieve study objectives and how it was determined, including clinical and statistical assumptions supporting any sample size calculations	6 and 10
Recruitment	#15	Strategies for achieving adequate participant enrolment to reach target sample size	6
**Methods: Assignment of interventions (for controlled trials)**			
Allocation: sequence generation	#16a	Method of generating the allocation sequence (eg, computer-generated random	NA- non-randomised trial

For peer review only

		numbers), and list of any factors for stratification. To reduce predictability of a random sequence, details of any planned restriction (eg, blocking) should be provided in a separate document that is unavailable to those who enrol participants or assign interventions	
Allocation concealment mechanism	#16b	Mechanism of implementing the allocation sequence (eg, central telephone; sequentially numbered, opaque, sealed envelopes), describing any steps to conceal the sequence until interventions are assigned	NA- non-blinded
Allocation: implementation	#16c	Who will generate the allocation sequence, who will enrol participants, and who will assign participants to interventions	NA- non-randomised trial
Blinding (masking)	#17a	Who will be blinded after assignment to interventions (eg, trial participants, care providers, outcome assessors, data analysts), and how	N/A – non-blinded

For peer review only

Blinding (masking): emergency unblinding	#17b	If blinded, circumstances under which unblinding is permissible, and procedure for revealing a participant's allocated intervention during the trial	NA – non-blinded
**Methods: Data collection, management, and analysis**			
Data collection plan	#18a	Plans for assessment and collection of outcome, baseline, and other trial data, including any related processes to promote data quality (eg, duplicate measurements, training of assessors) and a description of study instruments (eg, questionnaires, laboratory tests) along with their reliability and validity, if known. Reference to where data collection forms can be found, if not in the protocol	7
Data collection plan: retention	#18b	Plans to promote participant retention and complete follow-up, including list of any outcome data	9, 12 and 13

For peer review only

		to be collected for participants who discontinue or deviate from intervention protocols	
Data management	#19	Plans for data entry, coding, security, and storage, including any related processes to promote data quality (eg, double data entry; range checks for data values). Reference to where details of data management procedures can be found, if not in the protocol	13
Statistics: outcomes	#20a	Statistical methods for analysing primary and secondary outcomes. Reference to where other details of the statistical analysis plan can be found, if not in the protocol	10, 12 and 13
Statistics: additional analyses	#20b	Methods for any additional analyses (eg, subgroup and adjusted analyses)	12 and 13
Statistics: analysis population and missing data	#20c	Definition of analysis population relating to protocol non-adherence (eg, as randomised analysis), and any statistical	12

For peer review only

		methods to handle missing data (eg, multiple imputation)	
**Methods: Monitoring**			
Data monitoring: formal committee	#21a	Composition of data monitoring committee (DMC); summary of its role and reporting structure; statement of whether it is independent from the sponsor and competing interests; and reference to where further details about its charter can be found, if not in the protocol. Alternatively, an explanation of why a DMC is not needed	11,12 and 13
Data monitoring: interim analysis	#21b	Description of any interim analyses and stopping guidelines, including who will have access to these interim results and make the final decision to terminate the trial	11 and 12
Harms	#22	Plans for collecting, assessing, reporting, and managing solicited and spontaneously reported adverse events and other	9 and 10

For peer review only

		unintended effects of trial interventions or trial conduct	
Auditing	#23	Frequency and procedures for auditing trial conduct, if any, and whether the process will be independent from investigators and the sponsor	13
**Ethics and dissemination**			
Research ethics approval	#24	Plans for seeking research ethics committee / institutional review board (REC / IRB) approval	14
Protocol amendments	#25	Plans for communicating important protocol modifications (eg, changes to eligibility criteria, outcomes, analyses) to relevant parties (eg, investigators, REC / IRBs, trial participants, trial registries, journals, regulators)	13
Consent or assent	#26a	Who will obtain informed consent or assent from potential trial participants or authorised surrogates, and how (see Item 32)	7

For peer review only

Consent or assent: ancillary studies	#26b	Additional consent provisions for collection and use of participant data and biological specimens in ancillary studies, if applicable	7
Confidentiality	#27	How personal information about potential and enrolled participants will be collected, shared, and maintained in order to protect confidentiality before, during, and after the trial	13
Declaration of interests	#28	Financial and other competing interests for principal investigators for the overall trial and each study site	16
Data access	#29	Statement of who will have access to the final trial dataset, and disclosure of contractual agreements that limit such access for investigators	13
Ancillary and post trial care	#30	Provisions, if any, for ancillary and post-trial care, and for compensation to those who suffer harm from trial participation	7 – post trial safety monitoring

For peer review only

Dissemination policy: trial results	#31a	Plans for investigators and sponsor to communicate trial results to participants, healthcare professionals, the public, and other relevant groups (eg, via publication, reporting in results databases, or other data sharing arrangements), including any publication restrictions	14
Dissemination policy: authorship	#31b	Authorship eligibility guidelines and any intended use of professional writers	16
Dissemination policy: reproducible research	#31c	Plans, if any, for granting public access to the full protocol, participant-level dataset, and statistical code	13
**Appendices**			
Informed consent materials	#32	Model consent form and other related documentation given to participants and authorised surrogates	N/A – not included but can be requested from the Trial Manager Sarah Dunn sarah.dunn2@newcastle.ac.uk
Biological specimens	#33	Plans for collection, laboratory evaluation, and storage of biological specimens for genetic	N/A – not included, laboratory manual can be requested from the

For peer review only

or molecular analysis in the current trial and for future use in ancillary studies, if applicable	Trial Manager Sarah Dunn sarah.dunn2@newcastle.ac.uk

None The SPIRIT Explanation and Elaboration paper is distributed under the terms of the Creative Commons Attribution License CC-BY-NC. This checklist can be completed online using https://www.goodreports.org/, a tool made by the EQUATOR Network in collaboration with Penelope.ai

For peer review only

BMJ Open

Protocol for a multicentre, open label, prospective, single arm study of the safety and impact of eculizumab withdrawal in patients with atypical haemolytic uraemic syndrome

Journal:	*BMJ Open*
Manuscript ID	bmjopen-2021-054536.R1
Article Type:	Protocol
Date Submitted by the Author:	02-Jun-2022

Complete List of Authors:	Dunn, Sarah; Newcastle University, Newcastle Clinical Trials Unit<br>Brocklebank, Victoria; Newcastle University, National Renal Complement Therapeutics Centre<br>Bryant, Andrew; Newcastle University, Population Health Sciences Institute<br>Carnell, Sonya; Newcastle University, Newcastle Clinical Trials Unit<br>Chadwick, Thomas; Newcastle University, Population Health Sciences Institute<br>Johnson, Sally; Newcastle Upon Tyne Hospitals NHS Foundation Trust, Great North Children's Hospital ; Newcastle University, National Renal Complement Therapeutics Centre<br>Kavanagh, David; Newcastle University, Translational and Clinical Research Institute ; Newcastle University, National Renal Complement Therapeutics Centre<br>Lecouturier, Jan; Newcastle University, Population Health Sciences Institute<br>Malina, Michal; Newcastle Upon Tyne Hospitals NHS Foundation Trust, Great North Children's Hospital; Newcastle University, National Renal Complement Therapeutics Centre<br>Moloney, Eoin; Optimax Access LTD, Health Economics<br>Oluboyede , Yemi; Newcastle University, Population Health Sciences Institute<br>Weetman, Christopher; Newcastle University, Newcastle Clinical Trials Unit<br>Wong, Edwin; Newcastle University, Translational and Clinical Research Institute ; Newcastle University, National Renal Complement Therapeutics Centre<br>Woodward, Len; aHUS alliance Global Action<br>Sheerin, Neil; Newcastle University, Translational and Clinical Research Institute ; Newcastle University, National Renal Complement Therapeutics Centre

**Primary Subject Heading**:	Renal medicine
Secondary Subject Heading:	Qualitative research, Health economics
Keywords:	NEPHROLOGY, HEALTH ECONOMICS, QUALITATIVE RESEARCH

SCHOLARONE™
Manuscripts

Protocol for a multicentre, open label, prospective, single arm study of the safety and impact of eculizumab withdrawal in patients with atypical haemolytic uraemic syndrome

Details of corresponding author

Full name	Postal address	Email	ORCHID ID
Ms Sarah Dunn, MSc	Newcastle Clinical Trials Unit, Newcastle University, 1-4 Claremont Terrace, Newcastle Upon Tyne, England, NE2 4AE	Sarah.dunn2@ncl.ac.uk	0000-0002-1901-3931

Details of Co-authors

Full name	Department	Institution	City	Country	Email
Dr Victoria Brocklebank, MBBS	National Renal Complement Therapeutics Centre	Newcastle University	Newcastle Upon Tyne	England	Victoria.Brocklebank@newcastle.ac.uk
Mr Andrew Bryant, MSc	Population Health Sciences	Newcastle University	Newcastle Upon Tyne	England	andy.bryant@newcastle.ac.uk
Dr Sonya Carnell PhD	Newcastle Clinical Trials Unit	Newcastle University	Newcastle Upon Tyne	England	sonya_carnell@yahoo.com
Dr Thomas J Chadwick PhD	Population Health Sciences	Newcastle University	Newcastle Upon Tyne	England	thomas.chadwick@newcastle.ac.uk
Dr Sally Johnson MB ChB MRCPCH, PhD	Great North Children's Hospital, Translational and Clinical Research Institute and National Renal Complement Therapeutics Centre	Newcastle Upon Tyne Hospitals NHS Foundation Trust and Newcastle University	Newcastle Upon Tyne	England	sally.johnson15@nhs.net
Professor David Kavanagh, MB, ChB, MRCP, PhD, FRCP	Translational and Clinical Research Institute and National Renal Complement Therapeutics Centre	Newcastle University	Newcastle Upon Tyne	England	David.Kavanagh@newcastle.ac.uk
Ms Jan Lecouturier, MSc	Population Health Sciences	Newcastle University	Newcastle Upon Tyne	England	jan.lecouturier@newcastle.ac.uk

For peer review only

Dr Michal Malina, MD, PhD, FRCPCH	Great North Children's Hospital and National Renal Complement Therapeutics Centre	Newcastle Upon Tyne Hospitals NHS Foundation Trust And Newcastle University	Newcastle Upon Tyne	England	michal.malina@nhs.net
Mr Eoin Moloney, MSc	Health Economics	Optimax Access LTD	Newcastle Upon Tyne	England	eoin.moloney@optimaxaccess.com
Dr Yemi Oluboyede PhD	Population Health Sciences	Newcastle University	Newcastle Upon Tyne	England	yemi.oluboyede@newcastle.ac.uk
Mr Christopher Weetman, BSc	Newcastle Clinical Trials Unit	Newcastle University	Newcastle Upon Tyne	England	christopher.weetman@newcastle.ac.uk
Dr Edwin Kwan Soon Wong, MBChB, PhD MRCP	Translational and Clinical Research Institute and National Renal Complement Therapeutics Centre	Newcastle University	Newcastle Upon Tyne	England	edwin.wong@newcastle.ac.uk
Mr Len Woodward	N/A	aHUS alliance Global Action	Newcastle Upon Tyne	England	len.woodward@ntlworld.com
Professor Neil Sheerin, MBBS, PhD, MRCP	Translational and Clinical Research Institute and National Renal Complement Therapeutics Centre	Newcastle University	Newcastle Upon Tyne	England	Neil.Sheerin@newcastle.ac.uk

Word Count: 4653

For peer review only

ABSTRACT

Introduction

Atypical haemolytic uraemic syndrome (aHUS) is a rare, life threatening disease caused by excessive activation of part of the immune system called complement. Eculizumab is an effective treatment, controlling aHUS in 90% of patients. Due to the risk of relapse, lifelong treatment is currently recommended. Eculizumab treatment is not without problems, foremost being the risk of severe meningococcal infection, the burden of bi-weekly intravenous injections and the high cost.

This paper describes the design of the Stopping Eculizumab Treatment Safely in aHUS (SETS aHUS) trial that aims to establish whether a safety monitoring protocol, including the reintroduction of Eculizumab for those who relapse, could be a safe, alternative treatment strategy for patients with aHUS.

Methods and analysis

This is a multi-centre, non-randomised, open label study of Eculizumab withdrawal with continuous monitoring of thrombotic microangiopathy (TMA) related serious adverse events using the Bayes factor single arm design. 30 patients will be recruited to withdraw from Eculizumab and have regular blood and urine tests for 24 months, to monitor for disease activity. If relapse occurs, treatment will be restarted within 24 hours of presentation. 20 patients will remain on treatment and complete health economic questionnaires only. An embedded qualitative study will explore the views of participants.

Ethics and dissemination

A favourable ethical opinion and approval was obtained from the North East - Tyne & Wear South Research Ethics Committee. Outcomes will be disseminated via peer-reviewed articles and conference presentations.

Trial registration

EudraCT Number: 2017-003916-37

ISRCTN number: ISRCTN17503205

Date of Registration: 20 April 2018

Keywords

atypical Haemolytic Uraemic Syndrome, qualitative research, Complement, Eculizumab

Article summary

Strengths and limitations of this study

- This is the first UK trial to evaluate the safety of eculizumab withdrawal in patient with aHUS.

For peer review only

For peer review only - http://bmjopen.bmj.com/site/about/guidelines.xhtml

- This trial fulfils the NICE recommendation that a research programme, with robust methods, should be carried out to evaluate when stopping Eculizumab treatment or dose adjustment might occur.
- Clinical experience suggests if relapse occurs this will likely happen in the first 12 months of withdrawal however, this trial follows patients up for 24 months to capture those patients who may relapse after the 12-month point.
- The small number of aHUS patients on treatment in the UK is insufficient to conduct a standard parallel group, randomised controlled trial.
- COVID-19 has had an impact on recruitment.

Trial Status

This manuscript is based upon trial protocol version 7.0 dated 14th January 2021. The first patient was recruited in November 2018, recruitment ended on 31st January 2022 and planned last patient visit is November 2023.

For peer review only

INTRODUCTION

Atypical Haemolytic Uraemic Syndrome (aHUS) is a severe, life-threatening disease characterised by thrombocytopenia, microangiopathic haemolytic anaemia and Acute Kidney Injury (AKI), and other organ involvement. Historically it is associated with a poor prognosis, with 50% of patients developing end stage kidney disease or dying in the first year after presentation [1] and a high risk of disease recurrence after kidney transplantation [2]. Prior to 2011, treatment options were limited and relied on plasma infusion or exchange, but in many cases this treatment failed to influence the course of disease [1] and was itself associated with significant morbidity and mortality [3]. In the UK, the incidence of aHUS is 0.4-0.5 cases per million per year [4].

The complement system is part of the innate immune system and in healthy individuals is tightly regulated to prevent excessive activation. In 60-70% of patients with aHUS, a genetic variant or autoantibody increasing complement activation can be identified [10]. In these patients, excessive activation of complement leads to endothelial injury and thrombus formation. The underlying genetic variant that predisposes to disease has an influence on the severity of disease and the likelihood of recurrent disease developing after transplantation [5].

Eculizumab is a humanized monoclonal antibody that inhibits the function of C5, an important protein involved in complement activation. Two uncontrolled, open label trials involving 36 adult and adolescent patients demonstrated the efficacy of Eculizumab treatment for aHUS over a 26 week period [6]. Additional prospective studies in children [7] and adults [8] confirmed efficacy. Follow-up of the original cohort suggests that treatment for 2 years is associated with good, longer-term clinical outcomes [9]. On the basis of the initial trial results, Eculizumab was approved for the treatment of aHUS by the European Medicines Agency (EMA) [10] and U.S. Food & Drug Administration (FDA) [11] in 2011. The National Institute for Health and Care Excellence (NICE) published its evaluation in 2015, recommending that Eculizumab should be used for the treatment of aHUS [12]. A recommendation in the NICE evaluation was that funding was on the condition that there was a 'research programme with robust methods to evaluate when stopping treatment or dose adjustment might occur'.

Complement is part of the immune system, therefore, treatment with Eculizumab is immunosuppressive, in particular, increasing the risk of *Neisseria meningitidis* infection (1-2000 fold)[13, 14]. All patients are vaccinated against meningococcal infection before starting Eculizumab and in the UK continuous prophylactic antibiotics are recommended. Despite these recommendations, there have been 6 cases of meningococcal infection in the UK in patients on Eculizumab treatment for aHUS. We are also aware of uncommon infections occurring in this group (enteroviral pneumonitis and *Herpes simplex* meningitis) but whether these are attributable to Eculizumab treatment is unclear.

Although there have been reports of patients relapsing after the withdrawal of treatment [15], there is increasing evidence that continuous treatment is not required for all patients. From experience prior to the introduction of Eculizumab, the risk of relapse is greatest in the period immediately after first presentation with 82% of relapses in adults, and 57% of relapses in children occurring within the first year after disease onset.

For peer review only - http://bmjopen.bmj.com/site/about/guidelines.xhtml

Beyond the first year, only a further 20% of patients will relapse in the subsequent 5-10 years [1]. Therefore, a proportion of patients will not relapse after their initial presentation and will be on Eculizumab unnecessarily. In addition, with monitoring for relapse and early reintroduction of treatment, complications from relapse can be avoided [16-18].

In this trial we will test the safety of eculizumab withdrawal using a Bayesian trial design. The efficacy of self-monitoring will also be tested, and we will explore patients' and parents/legal guardians' views on how treatment and monitoring of disease can be delivered most effectively.

METHODS AND ANALYSIS

Objectives and outcome measures

Primary

The primary clinical objective is to determine the safety of Eculizumab withdrawal in patients with aHUS, measured by the number of patients with a Thrombotic microangiopathy (TMA) related Serious Adverse Event (SAE) defined as any of the following:

- Irreversible (>3 months) reduction in estimated glomerular filtration rate (eGFR) -not attributable to another cause:

In adults

- by ≥20% if the screening eGFR is <90mls/min/1.73m2,
- by >20% to a level <90mls/min/1.73m2 if the screening eGFR is >90mls/min/1.73m2.

In children

- by ≥20% if the screening eGFR is <75mls/min/1.73m2,
- by >20% to a level <75mls/min/1.73m2 if the screening eGFR is >75mls/min/1.73m2.

- An episode of AKI attributed to a TMA that requires renal replacement therapy.
- A non-renal manifestation of a TMA that require hospitalisation, cause irreversible organ damage or death.

Secondary

Clinical

1. Measure the effectiveness of a monitoring protocol to detect disease relapse following withdrawal of Eculizumab as assessed by:

- The proportion of patients who relapse and restart Eculizumab without the development of a TMA-related SAE.
- The time from the first clinical feature (symptom, positive urinalysis or laboratory result) of a relapse of TMA and the re-introduction of Eculizumab.

For peer review only - http://bmjopen.bmj.com/site/about/guidelines.xhtml

2. The relapse rate after withdrawal of Eculizumab as determined by the proportion of patients who relapse after Eculizumab is withdrawn.

3. The proportion of patients, currently on long-term treatment with Eculizumab, who can be maintained off treatment.

4. The period from withdrawal to relapse in those patients who restart treatment.

5. The change in estimated GFR as calculated by the CKD-EPI or modified Schwartz equations over the course of the study from baseline (day 0) to end of the study.

6. Important clinical and laboratory indicators of imminent relapse by review of reported symptoms, physical signs, urinalysis and laboratory results prior to the diagnosis of a relapse.

Health Economic

7. The costs and health outcomes (measured in terms of adverse events and quality-adjusted life years [QALYs]) for patients on standard care (not withdrawing from Eculizumab treatment) over the two-year trial duration:

- Healthcare Utilisation Questionnaires for non-withdrawal participants at Day 0, 14, 70,154, 252, 336, 504 and 672.
- Adverse Event Assessment at every visit from Day 7 (32 visits) for withdrawal participants.

8. QALYs estimated from responses to the EQ-5D-5L, and SF-36 and determinants of QALYs/utilities over the 24-month follow-up period. at Day 0, 14, 70,154, 252, 336, 504 and 672.

9. Model-based estimate of the costs and health consequences, with results presented in terms of cost per QALY gained, over the estimated lifetime of patients withdrawing from treatment compared with standard care.

Trial Design

This is a multi-centre, non-randomised, open label study of Eculizumab withdrawal with continuous monitoring of TMA related serious adverse events using the Bayes factor single arm design of Johnson and Cook [19]. The patients will self-select whether they wish to withdraw from Eculizumab and carry out the monitoring protocol or remain on treatment and be part of the Health Economic analysis-only. An economic analysis, informed by the results of this trial, will determine whether Eculizumab withdrawal, substituting treatment with a protocolised surveillance and treatment reintroduction strategy, is cost-effective. The patient visit schedule for the withdrawal cohort is shown in Figure 1, and the Health Economic cohort in Figure 2.

Trial setting

This multi-centre trial will be carried out in up to 20 adult and paediatric Renal units (secondary and tertiary care) in the UK who are using Eculizumab to treat patients with aHUS.

Eligibility

All patients must fulfil the following inclusion criteria in order to be eligible for the trial:

For peer review only - http://bmjopen.bmj.com/site/about/guidelines.xhtml

- Age ≥2+ years of age,
- On Eculizumab treatment for at least 6 months,
- In remission with no evidence of ongoing microangiopathic haemolytic anaemia (MAHA) activity at screening defined by:
  - Platelet count > lower limit of normal as determined by local reference range,
  - Lactate Dehydrogenase (LDH) <x2 upper limit of normal as determined by local lab reference ranges,
- Normal renal function or Chronic Kidney Disease (CKD) stages 1-3 (eGFR >30mls/min/1.73m^2),
- Absence of decline of renal function confirmed by review of available assessments of renal function for the preceding 6 months by the Chief Investigator (CI) and clinical members of the Trial Management Group (TMG).

The following inclusion criteria must be met only by those wishing to participate in the withdrawal component of the trial:

- Willing to attend for safety monitoring assessments,
- Willing to travel only to countries that can supply Eculizumab (to be confirmed with co-ordinating centre prior to travel),
- Able to perform or parent/guardian to perform and record self-monitoring urinalysis,
- Sexually active female patients must have a negative pregnancy test at screening and be using an effective contraception for the duration of the study.

OR

- fulfil one of the following criteria:
  - Be post-menopausal,
  - Have undergone surgical sterilisation.

The following exclusion criteria is applicable to all patients wishing to participate in the trial:

- Severe non-renal disease manifestations at initial presentation with aHUS, which in the opinion of the Chief Investigator and/or the clinical members of the TMG makes the risk of treatment withdrawal unacceptable,
- Current or planned pregnancy within the study duration,
- Unable to give informed consent or assent, or unable to obtain parent/guardian consent if under 16 years of age,
- Current participation in another clinical trial (not including participation in aHUS registries),
- Severe, uncontrolled hypertension (systolic blood pressure >160 mmHg) that is likely to induce at TMA.

The following exclusion criteria is applicable only to those wishing to participate in the withdrawal component of the trial:

- Loss of a previous transplant kidney to recurrent aHUS,
- Transplant recipient with a pathogenic mutation in *C3*, *CFH* or *CFB,*

For peer review only

For peer review only - http://bmjopen.bmj.com/site/about/guidelines.xhtml

- Haematuria rating of 3+.

Screening and Recruitment

30 patients will be recruited to withdraw from Eculizumab treatment, and 20 patients will be recruited who will remain on treatment and complete the Health Economic questionnaires only.

Patients with a diagnosis of aHUS receiving Eculizumab [4] to treat disease in native or transplanted kidneys will be identified by the National aHUS Service, which maintains a list of patients who fulfil these criteria as part of the NHS England commissioned service. Those patients who meet the genetic eligibility criteria will be highlighted to site teams who will carry out formal screening assessments. A physical examination and vital signs will be performed, and routine safety laboratory tests will be reviewed to ensure that a patient fulfils all eligibility criteria for entry into the study. Female participants withdrawing from treatment, who are of childbearing age and sexually active, will be required to have a negative pregnancy test prior to treatment withdrawal. Participants will also consent to have samples taken for exploratory analysis and storage at Newcastle University biobank for use in future research.

Consent will be sought from the parents/legal guardian on behalf of patients under the age of 16. Assent will be taken from those patients under 16 years old, as appropriate (supplement 1). No trial related procedures will be carried out prior to consent.

Intervention

Patients who consent to withdraw from Eculizumab will receive their last dose of Eculizumab during visit 1 (classed as day -14 prior to withdrawal).

Visit Details and Assessments

Baseline Assessments & Data collection for withdrawal cohort (Visit 2, Day 0 +/- 2 days)

Study day 0 will be the day that the participants would usually receive their next dose of Eculizumab, based on standard dosing schedules (+/-2 days). The Eculizumab will not be administered however, meningococcal prophylaxis will be continued for a further 2 weeks after day 0.

At day 0 of the study (Visit 2), participants will undergo the following assessments:
Vital signs (temperature, pulse and blood pressure), Height & weight, renal function (creatinine and eGFR), Urinalysis and urine protein/creatinine ratio, haemolysis markers including platelet count, haemoglobin, LDH, Electrolyte Profile, Liver Function (Bilirubin, ALT/AST, ALP, LDH, serum calcium, phosphate, albumin & total protein), haptoglobin (if available) and blood film, concomitant medication review, health-related quality of life questionnaires (EQ-5D-5L and SF-36) and health care utilisation questionnaire. A biomarker and complement activation sample to identify predictors of relapse (for example soluble C5b-9) is also taken and stored at site before transfer to Newcastle University.

For peer review only - http://bmjopen.bmj.com/site/about/guidelines.xhtml

Study Visit Assessments & Data collection for withdrawal cohort (Visits 3-34)

Participants will be assessed regularly for evidence of disease relapse for the 2-year duration of the study. The participants will attend a total of 32 safety monitoring visits over the 2-year withdrawal follow-up period.

Trial participants will be reviewed at the trial site weekly (+/- 2 days) for the first month, then alternate weeks (+/-2 days) until month 6, then monthly (+/- 7 days) thereafter until the end of the trial period (month 24). At each study visit, the participants will undergo the monitoring assessments as detailed in Figure 1.
Paediatric participants must have their weight recorded at every visit for calculation of eGFR. At the end of the trial, the level of safety monitoring for those patients who remain off treatment and disease free will be decided by their local clinical care team in discussion with the National aHUS Service.

Due to the COVID-19 pandemic, participants may be unable to attend their scheduled follow up visits or may be attending a local hospital to have safety bloods taken. If the participants are unable to attend site due to self-isolation or underlying health issues; where possible, a remote, follow-up call will be carried out by a member of the local research team. Participants will be asked to report changes to their concomitant medications, any adverse events experienced since their previous follow-up and the results of their home urinalysis tests.

Health Economic Assessments

Participants, or their parent/legal guardian, in both withdrawal and Health Economics cohorts will complete the EQ-5D-5L (proxy version if patient < 12 years), SF36 (parent/legal guardian completes if patient <14 years) and a health care utilisation questionnaire at 8 timepoints. A time and travel questionnaire is completed at 1 timepoint, as detailed in Figures 1 and 2.

Self-monitored Urinalysis

Withdrawal participants, or their parent/legal guardian, will be trained to perform and understand the results of home urinalysis. Urinalysis, for the presence of haematuria or haemoglobinuria as an indicator of intravascular haemolysis and therefore disease activity, will then be performed daily by the participant or parent/legal guardian for the first month and then three times per week for the remainder of the study period. The results will be recorded in a participant diary and will be reviewed at each study visit. Participants or their parent/legal guardian will report any significant change in urinalysis, not related to menstruation, using their own baseline result to guide them in relation to the thresholds as detailed in *Table 1*.

Table 1. Home Urinalysis result thresholds

**Baseline**	**urinalysis result threshold (not related to menstruation)**
Neg/Trace	++ on any occasion OR + on any two occasions 24 hours apart
+	+++ on any occasion OR ++ on any two occasions 24 hours apart
++	+++ on any occasion

For peer review only - http://bmjopen.bmj.com/site/about/guidelines.xhtml

If the threshold criteria are met, participants or their parent/legal guardian will contact their treatment site immediately to arrange an unscheduled visit to assess disease activity as outlined in *Figure 1*.

Trial Withdrawal

Participants will have the right to withdraw from the study at any time for any reason, and without giving a reason. The investigator will also have the right to withdraw participants from the study if she/he judges this to be in their best interest. Those participants who have been withdrawn from treatment can request to restart treatment, even if they have not relapsed. Data and blood samples provided by the participant up until the point of withdrawal will be included in analysis, unless they specifically request to have this removed. Participants who withdraw from the trial will not be replaced.

Change in Health Status

Participants will be advised to report any significant change in health status to the responsible site or local health care provider. Participants will be provided with a participant identification card to present to attending medical staff with details of the study, tests required and study centre and National aHUS Service contact details (Supplement 2). Sites will notify the participants' General Practitioner of their involvement in the study and inform them of the required action to be taken in the case of suspected relapse. Criteria for a diagnosis of aHUS relapse are shown in Figure 3.

If there is a clinical suspicion of disease activity, formal assessment will occur as outlined in *Figure 1*, Unscheduled visit column.

Any other adverse events that could represent a relapse will be discussed with the Investigators and/or the aHUS National Service. A decision to restart will be made according to current Service procedures.

Relapse Management

When a relapse is diagnosed, participants will restart Eculizumab treatment within 24 hours of presentation provided there is no evidence of an active infection that would be a contra-indication to treatment at the recommended dose of 900mg weekly for the first 4 weeks then 1200mg every two weeks thereafter (or age adjusted dose and regime). TMA activity will be monitored (platelet count, LDH) as recommended by attending clinician until haematological remission is achieved as defined in Figure 4.

Participants who relapse and require re-introduction of Eculizumab treatment will remain on treatment in study under follow up for the full 2 years of the study. Home urinalysis will not be required after re-introduction of Eculizumab treatment.

Participants will consent to travel to only those countries where Eculizumab is available. If a participant relapses while they are travelling outside of the country, the National aHUS Service will make arrangements with the

For peer review only - http://bmjopen.bmj.com/site/about/guidelines.xhtml

destination country to access and fund Eculizumab if required, with arrangements from the commissioning authority.

Embedded qualitative study

In-depth one-to-one telephone interviews will be conducted following a topic guide developed with the input of the research team, including PPI. The intention is to keep interviews very broad to ensure we capture the full experience of interviewees.

Up to 30 patients who withdraw from Eculizumab and up to 20 patients who decline to withdraw will be approached to participate. Up to 20 patients who withdraw will be re-interviewed at the end of the withdrawal period (24 months later) to explore their views of the monitoring protocol. This group will be asked at the first interview if they agree to be contacted again towards the end of the study for a follow up interview. Where possible, any patients who relapse and go back onto treatment will also be interviewed. Consent will be recorded at the time of the interview. Interviews will be digitally recorded with the permission of the interviewee, transcribed verbatim and anonymised.

Safety reporting

All Adverse Events (AEs) occurring from the point of withdrawal (day 0) to end of study participation will be recorded. SAEs occurring from the point of withdrawal (day 0) must be reported to The Newcastle Clinical Trials Unit (NCTU) within 24 hours of the site becoming aware of the event. Serious Adverse Reactions (SARs) and Suspected Unexpected Serious Adverse Reactions (SUSARs) are reportable only for those participants who have Eculizumab treatment re-initiated during their participation in the trial. The assessment of expectedness will be performed by the PI at site against the approved Reference Safety Information (RSI) for the trial (Section 4.8 of the Soliris SmPC).

Statistical Analysis

Sample size

A maximum of 30 patients will be recruited to the withdrawal component; this is judged to be reasonable given the rare nature of the disease. The specifics of this sample size are intrinsically linked to the Bayes factor single arm binary design employed to analyse the primary outcome measure. There is no allowance for loss to follow-up as this patient group is already subject to a high degree of clinical follow-up and death is defined as one of the serious events under consideration. 20 patients will be recruited to the non-withdrawal arm as a comparator group for health economic analysis only.

Analysis

This is a single arm, open label trial with the primary endpoint being a binary response (the presence/absence of a primary outcome event within the follow-up period). We will compare the rate of TMA-related serious

For peer review only - http://bmjopen.bmj.com/site/about/guidelines.xhtml

adverse events (primary outcomes) following the withdrawal of medication to that of treatment-related serious adverse events, expected under standard care.

The Bayes factor single arm binary model [19] will be used to monitor the trial. Based on historical data, the event rate (treatment-related serious adverse events in 100 patients over a 2 year period) for the standard of care is 0.06, and we expect that withdrawal of the treatment would give a rate of 0.12. This choice of rate has been informed in discussion with patients. Using this Bayesian hypothesis test-based design, we assume the rate is 0.06 under the null, and 0.12 under the alternative hypothesis.

We assume that the sample distribution of number of responses follows a binomial distribution and use an inverse moment prior for response under the alternative hypothesis.

For peer review only

Stopping Rules

A minimum of 5 patients will be enrolled before applying the stopping rules, and the cohort size for monitoring is 5 patients. The Data Monitoring Committee (DMC) can request earlier review if adverse events are reported before this point.

We implement two stopping rules:

(1) We will stop the trial for superiority (there being fewer TMA related serious adverse events on the intervention than would be expected under standard of care) if the posterior probability of the alternative hypothesis is less than 0.05, i.e. Pr(H1|Data) < 0.05;

(2) We will stop the trial for inferiority if the posterior probability of the alternative hypothesis is greater than 0.80, i.e., Pr(H1|Data) > 0.80.

Operating Characteristics and Stopping Boundaries

The operating characteristics (Table 2) and stopping boundaries (Table 3) were produced using the M. D. Anderson Cancer Center Department of Biostatistics software BayesFactorBinary, version 1.0 [20]

Table 2. Operating Characteristics

Scenario	True rate of treatment related serious adverse events	Probability of Stopping for Inferiority	Probability of Stopping for Superiority	Average number of patients treated (Percentiles: 10%, 25%, 50%, 75%, 90%)
1	0.06	0.096	0	28.44 (30, 30, 30, 30, 30)
2	0.12	0.443	0	23.48 (5, 15, 30, 30, 30)
3	0.18	0.753	0	17.76 (5, 10, 15, 30, 30)
4	0.24	0.928	0	13.72 (5, 5, 15, 15, 30)
5	0.30	0.982	0	10.52 (5, 5, 10, 15, 20)

If the true rate is 0.06 (Scenario 1, null hypothesis), the trial will stop with probabilities of 0.096 and 0 in favour of the alternative and null hypotheses, respectively. The average number of patients (10%, 90% percentiles) is

For peer review only - http://bmjopen.bmj.com/site/about/guidelines.xhtml

28.44 (30, 30). If the true rate is 0.12 (Scenario 2, alternative hypothesis), the trial will stop with probabilities of 0.443 and 0 in favour of the alternative and null hypotheses, respectively. The average number of patients (10%, 90%) is 23.48 (5, 30).

Table 3 – SETS aHUS Trial Stopping Boundaries

Number of patients (in complete cohorts of 5)	**Stop** the trial for Superiority if there are this many TMA **Events** (inclusive)	**Continue** the trial if there are this many TMA **Events** (inclusive)	**Stop** the trial for Inferiority if there are this many TMA **Events** (inclusive)
5	Never stop for superiority with this many patients	0-1	2-5
10 or 15	Never stop for superiority with this many patients	0-2	3-15
20	Never stop for superiority with this many patients	0-3	4-20
25 or 30	Never stop for superiority with this many patients	0-4 (The trial always stops at 30 patients, which is the maximum)	5-30

The study will stop for inferiority with 2 TMA related serious adverse events in the first cohort of 5 participants. Subsequently, the study would stop if 3 or more TMA related serious adverse events are observed in the first 15 participants, 4 or more in the first 20 participants, and 5 or more in the whole study population. We are well placed to respond to any negative safety signal.

1000 repetitions were used in the software simulation. Calculations with different numbers of repetitions resulted in unchanged stopping boundaries with only marginal changes to the operating characteristics.

There may be differing risk of relapse according to disease aetiology. However, the available numbers do not allow for risk strata to be monitored separately. The DMC will consider this within their remit.

In addition to this ongoing analysis, at the end of the study, data will also be reported descriptively, together with the number of patients recruited. Descriptive statistics reported will be selected as appropriate to the specific outcome measure. For proportion outcomes, the number of patients recording the event will also be reported.

Due to the sample size, no comparative statistical methods will be applied. There will be no imputation of missing data and a complete case analysis will be undertaken.

Subgroup Analyses

Except for the analysis of the primary outcome on an ongoing basis, the analyses described above may be reported separately for different genetic groups or risk strata.

Health Economic analysis

Within-trial assessments of costs and outcomes

For peer review only - http://bmjopen.bmj.com/site/about/guidelines.xhtml

Costs and health outcomes (measured in terms of resource use of primary and secondary health care NHS services and QALYs) associated with Eculizumab withdrawal (30 participants), compared with standard care (20 participants), will be assessed over the 24-month follow-up period. Information on costs and health outcomes will be recorded for each individual involved in both treatment groups. Data derived from the within-trial analysis will be assessed to understand the key determinants of differences in costs and outcomes between the two patient groups. Data will then be used to parameterise the lifetime economic model (combined with data from the literature).

Assessment of cost-effectiveness

An economic decision model will be developed to assess the cost-effectiveness of the alternative treatment options under evaluation. Costs and health consequences, measured in terms of QALYs, associated with Eculizumab withdrawal, and a policy of monitoring following withdrawal, and standard care, beyond the two-year timeframe of the trial will be captured. We propose to conduct a cost-utility analysis, with results presented in terms of incremental cost per QALY gained.

Qualitative analysis

We will take an inductive approach to data collection and analysis. This means there is no a priori theory; themes, concepts and theories will be elicited from the interview data when it is analysed and drawing upon relevant literature, PPI, and experts in the study team. Data will be analysed thematically using a constant comparative method. This entails a process of familiarisation with the data and then the development of a thematic framework. A small number of transcripts will be coded, and the framework amended accordingly. A second level analysis will be conducted using a constant comparative method. This involves a process of comparing and contrasting themes elicited from the data, within and across interviews [21]. NVivo will be used as a data management tool.

Trial Management and monitoring

This trial is sponsored by the Newcastle Upon Tyne Hospitals NHS Foundation Trust. The trial will be co-ordinated by a TMG that will include those individuals responsible for the day-to-day management of the trial. A Trial Steering Committee (TSC) made up of independent clinical and lay members will provide overall supervision of the trial. A Data Monitoring Committee (DMC) composed of independent clinicians and statistician will undertake independent review and monitor efficacy and safety endpoints. The trial was prospectively registered on the International Standard Registered Clinical/soCial sTudy Number (ISRCTN) registry and the European Union Drug Regulating Authorities Clinical Trials Database (EudraCT) (supplement 3). The Newcastle Clinical Trials Unit (NCTU) will be responsible for communicating protocol amendments to participating sites and carrying out central, remote, and on-site monitoring.

Confidentiality and data handling

For peer review only - http://bmjopen.bmj.com/site/about/guidelines.xhtml

Personal data will be regarded as strictly confidential. To preserve anonymity, a unique participant ID will be assigned to each participant at consent. Only the clinical team at the participating sites will have access to key data which links study identifiers to individual datasets. All study records and Investigator Site Files will be kept at site in a locked filing cabinet with restricted access.

Written consent will be sought from participants or legal guardians, if patient is under the age of 16 years, to allow access to their hospital records.

Data is recorded by authorised staff and stored in a secure web-based electronic data capture system (MACRO) designed and maintained by NCTU hosted on secure servers at Rackspace within the UK. Analysis of the data will be undertaken by the Newcastle University trial statisticians. Anonymised data from this trial may be available to the scientific community subject to regulatory and ethics approval. Requests for data should be directed to the corresponding author. All study data will be archived for 5 years.

For peer review only

Patient and public involvement (PPI)

A PPI representative sits on the Trial Management Group, was involved in protocol and study document development, and is involved in ongoing trial management discussions. We also have an aHUS patient as an independent member of the Trial Steering Committee.

ETHICS AND DISSEMINATION

A favourable ethical opinion and approval was obtained from the North East - Tyne & Wear South Research Ethics Committee in April 2018. Written informed consent will be obtained from all participants prior to their involvement in the trial. The results of the study will be submitted to peer-reviewed journals, presented at conferences and on the trial website.

DISCUSSION

This study will determine whether it is safe to withdraw Eculizumab using a trial methodology designed to detect an excess of adverse outcomes following withdrawal (primary endpoint). The study will also estimate the proportion of patients with aHUS that can be maintained off Eculizumab and test a system for surveillance to identify relapse early (secondary endpoints). This will allow a cost-utility analysis to be conducted, exploring the impact of treatment withdrawal [22]. This carefully monitored patient group will allow us to determine how early sub-clinical relapse can be detected using standard biochemical and haematological measurements and novel biomarkers of complement activation or tissue injury. An embedded qualitative study of patients, both those who withdraw and decide not to withdraw, will explore attitudes towards treatment and its withdrawal.

REFERENCES

1. Fremeaux-Bacchi V, Fakhouri F, Garnier A, et al. Genetics and outcome of atypical hemolytic uremic syndrome: a nationwide French series comparing children and adults. *Clin J Am Soc Nephrol* 2013, 8(4):554-562.
2. Noris M, Remuzzi G. Thrombotic microangiopathy after kidney transplantation. *Am J Transplant* 2010;10(7):1517-1523.
3. Johnson S, Stojanovic J, Ariceta G, et al. An audit analysis of a guideline for the investigation and initial therapy of diarrhea negative (atypical) hemolytic uremic syndrome. *Pediatric nephrology (Berlin, Germany)* 2014;29(10):1967-1978.
4. Sheerin NS, Kavanagh D, Goodship TH, et al. A national specialized service in England for atypical haemolytic uraemic syndrome-the first year's experience. *QJM* 2016;109(1):27-33.
5. Le Quintrec M, Zuber J, Moulin B, et al. Complement genes strongly predict recurrence and graft outcome in adult renal transplant recipients with atypical hemolytic and uremic syndrome. *Am J Transplant* 2013;13(3):663-675.
6. Legendre CM, Licht C, Muus P, et al. Terminal complement inhibitor eculizumab in atypical hemolytic-uremic syndrome. *The New England journal of medicine* 2013;368(23):2169-2181.
7. Greenbaum LA, Fila M, Ardissino G, et al. Eculizumab is a safe and effective treatment in pediatric patients with atypical hemolytic uremic syndrome. *Kidney international* 2016;89(3):701-711.
8. Fakhouri F, Hourmant M, Campistol JM, et al. Terminal Complement Inhibitor Eculizumab in Adult Patients With Atypical Hemolytic Uremic Syndrome: A Single-Arm, Open-Label Trial. *Am J Kidney Dis* 2016; 68(1):84-93.
9. Licht C, Greenbaum LA, Muus P, et al. Efficacy and safety of eculizumab in atypical hemolytic uremic syndrome from 2-year extensions of phase 2 studies. *Kidney international* 2015; 87(5):1061-1073.
10. European Medicines Agency: http://www.ema.europa.eu/docs/en_GB/document_library/EPAR_-_Assessment_Report_-_Variation/human/000791/WC500119185.pdf. 2011.
11. FDA: http://www.fda.gov/AboutFDA/CentersOffices/OfficeofMedicalProductsandTobacco/CDER/ucm273089.htm.
12. https://www.nice.org.uk/guidance/hst1/resources/eculizumab-for-treating-atypical-haemolytic-uraemic-syndrome-1394895848389
13. Struijk GH, Bouts AH, Rijkers GT, et al. Meningococcal sepsis complicating eculizumab treatment despite prior vaccination. *Am J Transplant* 2013;13(3):819-820.
14. McNamara LA, Topaz N, Wang X, et al. High Risk for Invasive Meningococcal Disease Among Patients Receiving Eculizumab (Soliris) Despite Receipt of Meningococcal Vaccine. *MMWR Morb Mortal Wkly Rep* 2017;66(27):734-737.

For peer review only - http://bmjopen.bmj.com/site/about/guidelines.xhtml

15. Carr R, Cataland SR. Relapse of aHUS after discontinuation of therapy with eculizumab in a patient with aHUS and factor H mutation. *Annals of hematology* 2013;92(6):845-846.

16. Ardissino G, Testa S, Possenti I, et al. Discontinuation of eculizumab maintenance treatment for atypical hemolytic uremic syndrome: a report of 10 cases. *Am J Kidney Dis* 2014;64(4):633-637.

17. Wijnsma KL, Duineveld C, Volokhina EB,et al. Safety and effectiveness of restrictive eculizumab treatment in atypical haemolytic uremic syndrome. *Nephrol Dial Transplant* 2018;33(4):635-645.

18. Fakhouri F, Fila M, Hummel A, et al. Eculizumab discontinuation in children and adults with atypical haemolytic uremic syndrome: a prospective multicentric study. *Blood* 2020.

19. Johnson VE, Cook JD: Bayesian design of single-arm phase II clinical trials with continuous monitoring. *Clin Trials* 2009; 6(3):217-226.

20.https://biostatistics.mdanderson.org/SoftwareDownload/SoftwareFiles/BayesFactorBinary/UsersGuide_BayesFactorBinary.pdf

21. Glaser BG. The Constant Comparative Method of Qualitative Analysis. *Social Problems* 1965;12(4):436-45.

22. Drummond MF, O'Brien B, Stoddart GL, et al. Basic Types of Economic Evaluation. In: *Methods for economic evaluation of health care programmes.* edn. Oxford University Press; 2005.

For peer review only

Acknowledgements

The authors would like to thank Dr Colin Muirhead who was responsible for the statistical proposal in the initial grant application.

The authors would also like to thank all the Principal Investigators for their contribution and support, the research nurses, the members of the TSC and DMC and all the patients involved in the SETS aHUS Trial.

Contributors

Professor Neil Sheerin is the Chief Investigator and senior author of the SETS aHUS design and has led on grant acquisition and protocol development. Professor David Kavanagh, Dr Sally Johnson, Dr Ewin Kwan Soon Wong, Mr Len Woodward, Ms Jan Lecouturier, Dr Thomas Chadwick, and Dr Yemi Oluboyede are co-applicants of the grant and contributed to the design of the trial and protocol development. Dr Sonya Carnell and Ms Sarah Dunn are part of the trial management team and contributed to protocol development. Mr Eoin Moloney is a Health Economist and contributed to protocol development. Mr Andy Bryant is a research statistician and contributed to protocol development. Mr Christopher Weetman is a data manager and contributed to protocol development. Dr Michal Malina and Dr Victoria Brocklebank are part of the National Renal Complement Therapeutics Centre and have played key roles in the running of the SETS aHUS trial.

This paper was drafted from the current approved version of the protocol by the corresponding author SD; all contributing authors commented and amended drafts of the paper. All contributing authors read and approved the final manuscript.

Funding

The SETS aHUS Trial is funded by the National Institute for Health Research (NIHR) Health Technology Assessment (HTA) Programme (project number 15/130/94). The views expressed are those of the authors and not necessarily those of the NIHR or the Department of Health and Social Care.

Conflict of interests

SB has received honoraria for sitting on advisory boards for Alexion and Novartis. DK is a director of and scientific advisor to Gyroscope Therapeutics. DK received advisory board payments from Idorsia, Novartis, ChemoCentryx, Alexion, Apellis, Biomarin and Sarepta. DK’s spouse works for GSK. MM has received honoraria for educational talks and honorarium for national lead of aHUS registry, both from Alexion and travel expenses from Alexion. EKSW has received honoraria for lectures and/or advisory boards for Alexion Pharmaceutical, Biocryst and Novartis. LW has received expenses, honoraria and fees for advisory board participation and talks from Alexion and Roche. NS has given lectures or sat on advisory boards for Alexion Pharmaceutical, Roche, Astra Zeneca and Novartis, no personal honoraria, all payments made to the department.

Patient and public involvement

For peer review only

Patients and/or the public were involved in the design, or conduct, or reporting, or dissemination plans of this research. Refer to the Methods section for further details.

Patient consent for publication

Not required.

Provenance and peer review

Not commissioned; externally peer reviewed during funding application process.

Figure Legends

Figure 1. Data Collection Time Points for Withdrawal cohort

Figure 2. Data Collection Time Points for Health Economics Cohort

Figure 3. Criteria for diagnosis of aHUS relapse

Figure 4. Haematological remission definition and maintenance

For peer review only

	Central Pre-Screen	Site Screen and Consent	Final Infusion	Withdrawal Phase																																	
Month	0	0	0	1					2		3		4		5		6		7	8	9	10	11	12	13	14	15	16	17	18	19	20	21	22	23	24	Unscheduled Visit
Visit Number	N/A	N/A	1	2	3	4	5	6	7	8	9	10	11	12	13	14	15	16	17	18	19	20	21	22	23	24	25	26	27	28	29	30	31	32	33	34	
Genetic Eligibility	X																																				
Medical History Review		X																																			
Informed consent		X																																			
Eligibility Checklist Completion		X																																			
Physical Examination		X																						X												X	X
Height & Weight		X																																			
Pregnancy test		X																																			
Eculizumab Infusion			X																																		
meningococcal prophylaxis			X	X																																	
urine analysis training				X																																	
Vital Signs		X		X	X	X	X	X	X	X	X	X	X	X	X	X	X	X	X	X	X	X	X	X	X	X	X	X	X	X	X	X	X	X	X	X	X
Concomitant medication Review		X		X	X	X	X	X	X	X	X	X	X	X	X	X	X	X	X	X	X	X	X	X	X	X	X	X	X	X	X	X	X	X	X	X	X
Renal Function (Creatinine & GFR)		X		X	X	X	X	X	X	X	X	X	X	X	X	X	X	X	X	X	X	X	X	X	X	X	X	X	X	X	X	X	X	X	X	X	X
Liver Function Tests		X		X	X	X	X	X	X	X	X	X	X	X	X	X	X	X	X	X	X	X	X	X	X	X	X	X	X	X	X	X	X	X	X	X	X
Haemolysis markers (full blood count & LDH)		X		X	X	X	X	X	X	X	X	X	X	X	X	X	X	X	X	X	X	X	X	X	X	X	X	X	X	X	X	X	X	X	X	X	X
Electrolyte profile (U&Es)		X		X	X	X	X	X	X	X	X	X	X	X	X	X	X	X	X	X	X	X	X	X	X	X	X	X	X	X	X	X	X	X	X	X	X
Haptoglobin & Blood film		X		X				X				X						X						X						X						X	X
Urine PCR		X		X				X				X						X						X						X						X	X
Biomarkers and complement		X		X	X	X		X		X		X						X			X			X						X						X	X

activation sample																																					
Home Urinalysis		X		Daily					3 times per week																												
Home Urinalysis diary review					X	X	X	X	X	X	X	X	X	X	X	X	X	X	X	X	X	X	X	X	X	X	X	X	X	X	X	X	X	X	X	X	
Adverse Events					X	X	X	X	X	X	X	X	X	X	X	X	X	X	X	X	X	X	X	X	X	X	X	X	X	X	X	X	X	X	X	X	X
EQ5D & SF36				X		X					X						X				X			X						X						X	
Health care Utilisation questionnaire				X		X					X						X				X			X						X						X	
Time & Travel Questionnaire																														X							

For peer review only

Follow - up								
month	1		3	6	9	12	18	24
visit number	1	2	3	4	5	6	7	8
Medical History Review	X							
Informed consent	X							
Eligibility Checklist	X							
EQ5D & SF36	X	X	X	X	X	X	X	X
Health care Utilisation questionnaire	X	X	X	X	X	X	X	X
Time & Travel Questionnaire							X	

For peer review only

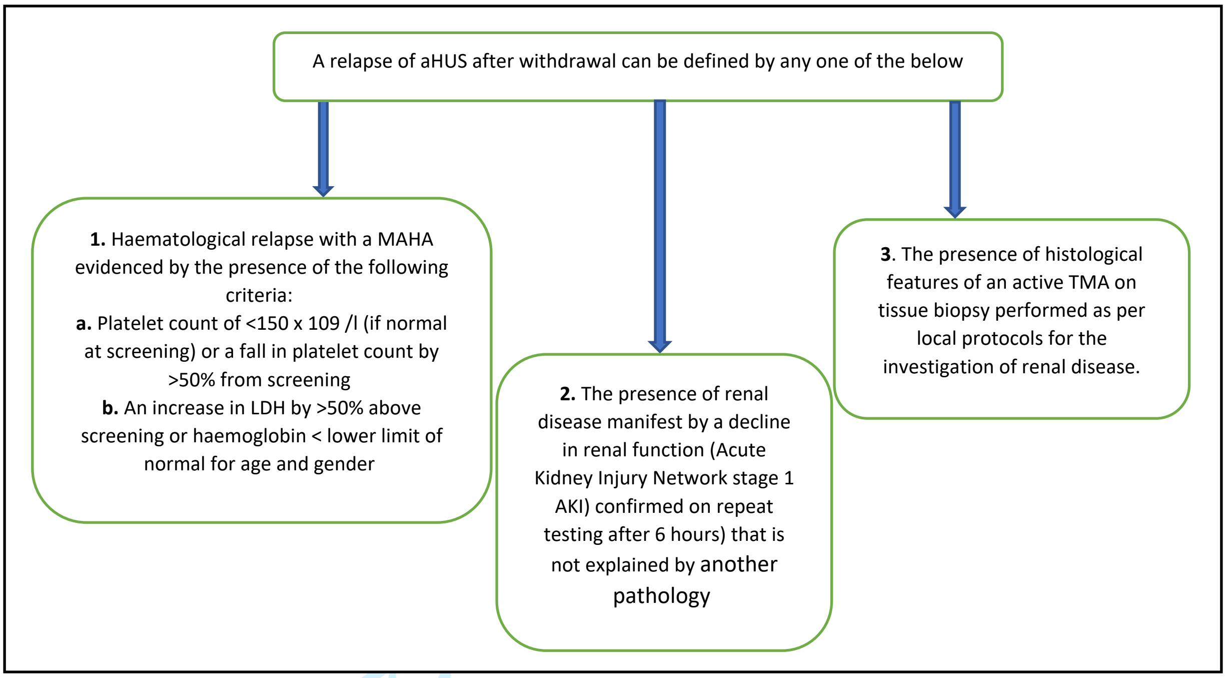

For peer review only

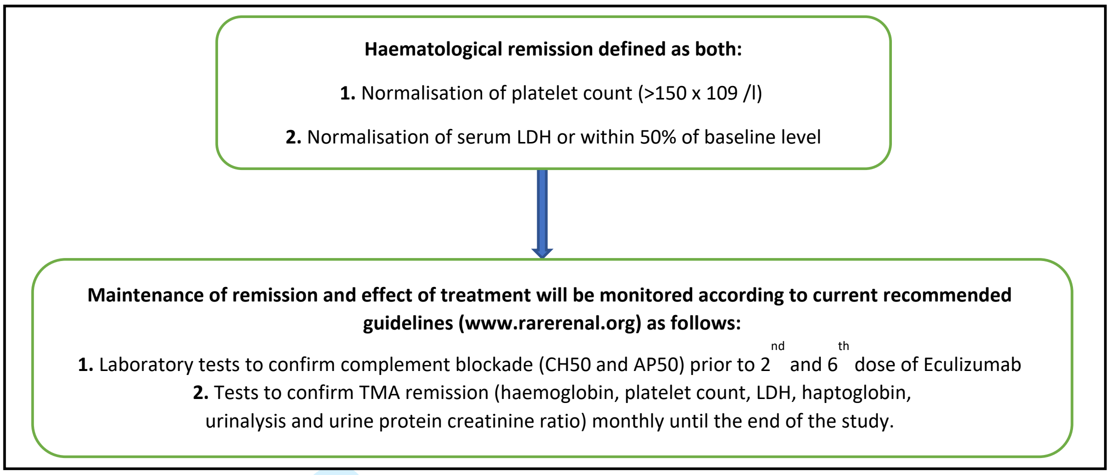

For peer review only

[Trust Headed Paper]

SETS aHUS: **S**topping **E**culizumab **T**reatment **S**afely in **aHUS**

Participant Information Sheet (aged 16+)
Version 3.0, 08.02.19

Invitation

You are being invited to take part in a research study. Please read the following information to help you decide if you want to take part. We would like you to understand why we are doing this research and what it means for you. You do not need to make a decision straight away, so please feel free to talk to others about the study if you wish. Please ask us if there is anything that is not clear or if you want to know more.

Please remember that you do not have to take part and your normal healthcare will not be affected in any way, whatever you decide.

Part 1

What is the purpose of this study?

Atypical Haemolytic Uraemic Syndrome (aHUS) is a rare disease. When aHUS occurs the cells that line the blood vessels are damaged and are no longer able to stop blood from clotting. Blood clots form in small vessels, particularly in the kidney, leading to problems with kidney function. Most cases are due to abnormalities in a part of the immune system called the complement system. These abnormalities lead to excessive activation of the complement system, which is responsible for the cell damage and blood clots.

Current standard treatment for aHUS involves a long-term intravenous injection of a drug called Eculizumab every 2 weeks. Eculizumab blocks the body's complement system and its ability to damage its own vulnerable cells.
Research has shown that Eculizumab is effective in the treatment of aHUS, but the recommendation that Eculizumab treatment should be lifelong is not based on strong evidence and may not be necessary for many patients.

This study hopes to provide evidence for an alternative strategy for treatment of aHUS based on monitoring and treatment re-introduction rather than continuous Eculizumab treatment.

Overall aim: To establish an alternative and safe treatment strategy for patients with aHUS that includes withdrawal of Eculizumab treatment.
Thank you for reading so far – if you are still interested, please read the rest of this leaflet which gives more detailed information about the trial and what will happen if you decide to take part.

For peer review only - http://bmjopen.bmj.com/site/about/guidelines.xhtml

[Trust Headed Paper]

Why have I been chosen to take part?

You have been asked to take part in the study because you have Atypical Haemolytic Uraemic Syndrome and are currently receiving Eculizumab treatment. We are hoping to recruit 30 patients to the withdrawal study and an additional 20 patients who will remain on Eculizumab and complete a series of questionnaires only.

What would taking part involve?

Taking part would involve stopping your Eculizumab treatment and attending 34 safety monitoring visits at hospital over the course of 24 months during which we will assess that you are safe to continue to remain off medication. If you currently receive your treatment at home you would need to be willing to attend the hospital to complete the safety visits. You will be required to test your urine at home and document the results daily (for the first month), and then three times per week for the remaining 23 months, in a patient diary provided to you by the study team. You will also complete questionnaires on 8 occasions relating to quality of life and measure use of NHS resources and your own out of pocket expenses related to health care.
If you do not want to stop your Eculizumab treatment but still want to participate you can also complete study questionnaires that relate to your quality of life, and measure use of NHS resources and your own out of pocket expenses related to health care. You would only be asked to complete these on 8 occasions over the 24-month period.

There is also a linked interview study that you can take part in whether you decide to withdraw from treatment or continue with Eculizumab treatment. If you would like to receive information about this study you can consent to have your contact details passed to the research team for them to contact you about this.

What are the possible benefits of taking part?

It might mean that you will no longer need to take the Eculizumab treatment and face any potential risks or side effects associated with treatment. Patients are about a thousand times more likely to develop a serious, potentially life threatening infection with meningococcus, a bug that causes meningitis or sepsis. Vaccination, and even antibiotics, do not give complete protection from this. Other serious problems have also been reported in patients taking Eculizumab but we cannot be certain that Eculizumab was the cause of these problems.

Being in the study will mean that you will no longer need bi-weekly infusions and will not have to continue taking additional antibiotics to prevent infection. However, it is possible that you could need to restart and continue to receive Eculizumab treatment if a relapse was to occur.

For peer review only - http://bmjopen.bmj.com/site/about/guidelines.xhtml

[Trust Headed Paper]

What are the possible risks of taking part?

The withdrawal of Eculizumab treatment could lead to a relapse of atypical HUS and relapse associated complications. When a relapse is diagnosed, your Eculizumab treatment will be restarted within 24 hours of presentation. It is essential that you present to a hospital with your patient card as soon as you begin to feel unwell or the home urine test shows an increase in the level of blood. This is to ensure that the Eculizumab is re-started as soon as possible to reduce the likelihood of kidney damage and associated complications. If your treatment is re-started, you will receive your first infusion in the hospital. You will also have the antibiotics restarted to protect you from infection. If you need to be put back on to Eculizumab treatment you can decide to receive your infusions in hospital or at home. This can be arranged with your clinical team if you relapse while in the study.

If a relapse occurs this could lead to:

- A drop in kidney function
- Other problems related to the disease which can affect organs such as the pancreas or nervous system

The evidence that is available at the moment suggests that if Eculizumab is reintroduced quickly these problems can be avoided. This is not proven, and this study will test whether this is true.

If, during the trial, you decide to travel outside of the country, we ask that you only travel to countries where Eculizumab is available, so treatment can be re-started immediately as required. You **MUST** first inform your hospital to check whether Eculizumab is available in the country that you plan to travel to and inform them of the dates that you will be outside of the country. While the cost of the Eculizumab will be covered by NHS England you must ensure that you have appropriate travel insurance and inform your insurance company that you are taking part in the trial. This is to ensure that all other treatment costs and hospital stays abroad are covered by your own insurance should you relapse. We have developed a travel guide to be followed should you decide to travel outside of the country while you are participating in the withdrawal study.

To minimise the risks of taking part in the study, we will only include you in the study if you:

- Have been on Eculizumab treatment for at least 6 months;
- Are in remission;
- Have a stable kidney function;
- Are willing to attend for safety monitoring assessments;
- Are willing to travel only to countries that can supply Eculizumab (to be confirmed with co-ordinating centre prior to travel);
- Are able to perform and record self-monitoring urinalysis.

For peer review only - http://bmjopen.bmj.com/site/about/guidelines.xhtml

[Trust Headed Paper]

Sexually active females of child bearing age must:

- Have a negative pregnancy test at screening and be using an effective contraception for the duration of the study (please ask your doctor/nurse).

  OR

- fulfil one of the following criteria:
  - Be post-menopausal
  - Have undergone surgical sterilisation

You will not be able to take part if you:

- have lost a previous transplant kidney to recurrent aHUS;
- Are currently or are planning pregnancy;
- Are unable to comply with safety monitoring assessments;
- Are currently participating in another clinical trial (not including participation in aHUS registries)

What will happen to me if I take part?

If you consent to withdraw from Eculizumab Treatment, you will complete the following visits:

Screening Visit

You will be asked to attend a screening visit at the hospital or clinic where you usually receive your Eculizumab treatment or if you receive your treatment at home, you will be asked to attend the hospital where your clinician is based. If you decide to take part you will complete a consent form. You will be given a unique patient ID number that will be written on your consent form and all questionnaires and samples that you provide in the study. This ID number will only be linked to you at your hospital and so nobody outside of your direct clinical care team will know that the information and samples belong to you.
If you are a female of child bearing age you will be required to have a pregnancy test at the screening visit after you have signed the consent form. You will receive your last Eculizumab infusion at the screening visit. Please be aware that you will continue to take your meningococcal prophylaxis for 4 weeks following this last Eculizumab infusion.
We will also collect information from your medical notes about your medical history after you have signed the consent form.

Baseline Visit

Your baseline visit will be 2 weeks (+/-2 days) after your screening visit on the day that you would usually receive your next dose of Eculizumab. You will not receive the infusion but will

For peer review only - http://bmjopen.bmj.com/site/about/guidelines.xhtml

attend your usual hospital or clinic to have your blood pressure and temperature checked. You will also have a blood and urine test and complete questionnaires about your health.

Study Visits

You will be reviewed at the hospital weekly for the first month, then alternate weeks until month 6, then monthly thereafter until the end of the study period (month 24).
At each study visit you will have a blood and urine test and answer questions about how your health has been since your last visit.

You will also be required to complete questionnaires at your hospital visits twice in month 1, and once in the following months, 3, 6, 9, 12, 18 & 24. These will ask about your health, quality of life, medications and about use of NHS services and your out of pocket expenses relating to health care. It should take between 20 and 30 minutes to complete these questionnaires each time.

Overall you will be attending 34 visits over the 24 month period which includes the screening and baseline visit. You will not be required to attend more visits than you would usually if you were to stay on your current treatment. If you currently receive your Eculizumab infusions at home you will need to come in to the hospital for the study visits as these cannot be carried out at your home.

You will be required to carry out urine tests at home and to document this in a patient diary. You will be given a 30-minute training session during your baseline visit that will explain everything that you will need to do to carry this out at home.

You will also be given a small card to carry which will include information on the trial. If you attend **ANY** hospital or clinic for treatment outside of your scheduled follow-up visits you **MUST** give the doctor this card so that they are able to contact the trial team.

If you continue with your Eculizumab treatment and consent to completing the questionnaires only;
You will receive the questionnaires by post on 8 occasions over the 24-month period. This should take you around 20-30 minutes to complete. You will be required to post the completed questionnaires back to the research team at Newcastle University using the pre-paid envelope supplied. The questionnaires will not contain any identifiable information relating to you as your unique patient ID will be written on the questionnaires when you receive them.

Will participating in research affect my treatment?

If you decide to take part in the withdrawal component of the trial then you will no longer receive your Eculizumab treatment. If you decide to take part in the questionnaire part of the trial only, your Eculizumab treatment will remain the same.

For peer review only - http://bmjopen.bmj.com/site/about/guidelines.xhtml

What will happen if I don't want to carry on with the study?

You may withdraw from the study at any time without giving a reason. If you decide to go back on to Eculizumab treatment we would like you to continue to be followed up by the study team. You may wish to withdraw completely from the study and further follow up and so we would not collect any further information about you to be used in the study. However, we would like to use the information and blood samples previously provided. If you decide that you don't want any of the information or blood samples already provided to be used in the study please contact a member of the study team so it can be removed from analysis.

Will I be paid for taking part?

If you receive re-imbursement for travel to clinics this will continue. If you currently receive your Eculizumab treatment at home, you may qualify to be reimbursed for travel to clinics for your follow up visits. Your doctor can give you more information about this. You will not be paid for taking part in this study.

Part 2

Will my GP be told about my involvement in this study?

If you decide to take part in this study and consent to have your GP informed, then we will inform your GP. Your participation in the study will also be noted in your medical records.

Will my taking part in research be kept confidential?

All of the data we collect will be kept strictly confidential and in accordance with the General Data Protection Regulation (GDPR).

The Newcastle Upon Tyne Hospitals NHS Foundation Trust (NUTH) is the sponsor for this study based in the United Kingdom and will act as the "data controller" for this study. **They are responsible for looking after your information and using it properly.** This study is managed on behalf of the sponsor by the Newcastle Clinical Trials Unit (NCTU) who will act as the "data processor". As data processor, NCTU are responsible for processing personal data on behalf of the data controller. The Newcastle Clinical Trials Unit based at Newcastle University would like to receive a copy of your consent form for safety purposes. This will be destroyed once it has been reviewed. No other identifiable information will be received by the Newcastle Clinical Trials Unit

Your rights to access, change or move your information are limited, as we need to manage your information in specific ways in order for the research to be reliable and accurate. If you withdraw from the study, we will keep the information about you that we have already

For peer review only - http://bmjopen.bmj.com/site/about/guidelines.xhtml

[Trust Headed Paper]

obtained. To safeguard your rights, we will use the minimum personally-identifiable information possible.

You can find out more about how we use your information at www.newcastle-hospitals.org.uk/about-us/freedom-of-information_how-we-use-information.aspx

To find out more information about research and general use of patient information please refer to the Health Research Authority Website at www.hra.nhs.uk/information-about-patients

The local study team at [NHS site] will use your name, [NHS number/CHI number] and contacts details to contact you about the research study, and make sure that relevant information about the study is recorded for your care, and to oversee the quality of the study. Individuals from the sponsor, Newcastle Clinical Trials Unit and regulatory organisations may look at your medical and research records to check the accuracy of the research study. The local study team will pass these details to the sponsor or the Newcastle Clinical Trials Unit along with information collected from you and/or your medical records. The only people at sponsor or the Newcastle Clinical Trials Unit who will have access to information that identifies you will be people who need to audit the data collection process. The people who analyse the information will not be able to identify you and will not be able to find out your name, [NHS number/CHI number] or contact details.

The local study team at [NHS site] will keep identifiable information about you from this study for 5 years after the study has finished.

When you agree to take part in a research study, the information about your health and care may be provided to researchers running other research studies in this organisation and in other organisations. These organisations may be universities, NHS organisations or companies involved in health and care research in this country or abroad. Your information will only be used by organisations and researchers to conduct research in accordance with the UK Policy Framework for Health and Social Care Research and will not contain any personal identifiable information such as your name, [NHS number/CHI number] or contact details.

This information will not identify you and will not be combined with other information in a way that could identify you. The information will only be used for the purpose of health and care research and cannot be used to contact you or to affect your care. It will not be used to make decisions about future services available to you, such as insurance.

The blood samples that you provide will be sent to Newcastle University to be analysed and will have your unique ID number on them but no personal identifiable information attached. Once the study is finished, these samples will be stored at the Newcastle University biobank and may be used in future aHUS research. Please let us know if you do not want your samples used in future research.

For peer review only - http://bmjopen.bmj.com/site/about/guidelines.xhtml

[Trust Headed Paper]

What will happen to the results of the research study?

Whenever possible we will publish the results of our studies in scientific journals. We also plan to present data at scientific conferences. You will not be named in any publication or presentation of the study results. We would also like to send you a newsletter with a summary of our results. Please let the research team know if you want to receive the newsletter. Results will also be available at the end of the study on the atypical HUS website which can be found at the following web address:
http://www.atypicalhus.co.uk/

Who is organising and funding the research?

This study is funded by the National Institute for Health Research (NIHR). The study is sponsored and indemnified by the Newcastle Upon Tyne NHS Foundation Trust and indemnified by Newcastle University. The Newcastle Clinical Trials Unit is managing the study.

Who has reviewed the study?

All research in the NHS is looked at by an independent group of people called a NHS Research Ethics Committee (REC). This is to protect your interests. This study has been reviewed and given a favourable opinion by the North East – Tyne & Wear South Research Ethics Committee and has been approved by the NHS Health Research Authority (HRA). The study has also been given approval by the Medicines and Healthcare products Regulatory Agency (MHRA).

What if relevant new information becomes available?

The study team will ensure that you are receiving the most appropriate and up to date medical care that you require.

What if something goes wrong?

If you have a concern about any aspect of the study please contact your local doctor (see contact details below). Alternatively, you can contact one of the researchers running this study and discuss your concerns.

Your local contact people for the study are:

Contact Details of local PI:	Contact details of local Research Nurse:
Name:	Name:
Address:	Address:
Phone:	Phone:
Email:	Email:

For peer review only - http://bmjopen.bmj.com/site/about/guidelines.xhtml

[Trust Headed Paper]

The Newcastle Trial Team contact is:

Contact Details of Trial Manager:
Name: Sarah Dunn
Address: 1-2 Claremont Terrace,
NCTU, Newcastle University, NE2 4AE
Phone: 0191 208 2521
Email: sarah.dunn2@ncl.ac.uk

If you are still unhappy and wish to complain formally and confidentially you can do this through the NHS complaints procedure by speaking to a member of the PALS (Patient Advise and Liaison Service) on 0800 0320 202 or by visiting www.PALS.nhs.uk.

In the event that something goes wrong and you are harmed during the research due to someone's negligence, then you may have grounds for a legal action for compensation against Newcastle upon-Tyne Hospitals NHS Foundation Trust but you may have to pay your legal costs.

How have patients and the public been involved in this study?

Our patient representative group has helped to design the study and study documents.

The group will meet when the trial is near completion to have input into the interpretation of the results. Their views of withdrawal and the safety results from the study will be considered. This will inform the publication of results. The groups views on the dissemination of results will also be considered.

Thank you for taking time to read this information sheet

For peer review only - http://bmjopen.bmj.com/site/about/guidelines.xhtml

SETS aHUS: Stopping Eculizumab Treatment Safely in aHUS

The holder of this card has a rare disease known as atypical Haemolytic Uraemic Syndrome and is taking part in a clinical trial to assess the safe withdrawal of Eculizumab treatment. Because of this, the holder of this card may suffer a relapse.

If the holder presents unwell, however minor the illness, please evaluate immediately and obtain the following Laboratory investigations as their Eculizumab treatment and prophylactic antibiotics may need to be re-started as soon as possible and within 24hrs:

• U&E • FBC • LDH

If the results are abnormal, immediately contact the local medical team and refer to the UK National aHUS Service website for advice. Contact details on reverse side.

For peer review only - http://bmjopen.bmj.com/site/about/guidelines.xhtml

SETS aHUS: Stopping Eculizumab Treatment Safely in aHUS

SETS aHUS

Patients Local Medical team: +44 (0)……………………………

UK National aHUS Service: +44 (0)191 2820385

(from 9am to 5pm GMT)

Outside of these hours, please contact the Newcastle Hospitals Switchboard and request to speak to the clinician on call for the aHUS Service on +44 (0)191 233 6161

www.atypicalhus.co.uk

For peer review only - http://bmjopen.bmj.com/site/about/guidelines.xhtml

Primary registry and trial identifying number	ISRCTN<br>ISRCTN17503205
Date of registration in primary registry	20 April 2018
Secondary identifying numbers	EudraCT: 2017-003916-37
Source(s) of monetary or material support	NIHR HTA
Primary sponsor	Newcastle Upon Tyne Hospitals NHS FT – Christopher Price christoper.price6@nhs.uk
Secondary sponsor(s)	N/A
Contact for public queries	Trial Manager – Sarah Dunn sarah.dunn2@newcastle.ac.uk
Contact for scientific queries	Chief Investigator – Professor Neil Sheerin neil.sheerin@newcastle.ac.uk
Public title	Stopping Eculizumab Treatment Safely in aHUS (SETS aHUS)
Scientific title	Multicentre, open label, prospective, single arm study of safety impact of Eculizumab withdrawal
Countries of recruitment	England and Scotland
Health condition(s) or problem(s) studied	atypical Haemolytic Uraemic Syndrome
Intervention(s)	Withdrawal of Eculizumab
Key inclusion and exclusion criteria	Key inclusion criteria:<br>• Age ≥2+ years of age,<br>• On Eculizumab treatment for at least 6 months,<br>• In remission with no evidence of ongoing microangiopathic haemolytic anaemia (MAHA) activity at screening defined by:<br>- Platelet count > lower limit of normal as determined by local reference range,<br>- Lactate Dehydrogenase (LDH) <x2 upper limit of normal as determined by local lab reference ranges,<br>• Normal renal function or Chronic Kidney Disease (CKD) stages 1-3,<br>• Absence of decline of renal function confirmed by review of available assessments of renal function for the preceding 6 months by the Chief

	Investigator (CI) and clinical members of the Trial Management Group (TMG).<br>Key Exclusion Criteria:<br>• Severe non-renal disease manifestations at initial presentation with aHUS, which in the opinion of the Chief Investigator and/or the clinical members of the TMG makes the risk of treatment withdrawal unacceptable,<br>• Current or planned pregnancy within the study duration,<br>• Unable to give informed consent or assent, or unable to obtain parent/guardian consent if under 16 years of age,<br>• Current participation in another clinical trial (not including participation in aHUS registries),<br>• Severe, uncontrolled hypertension (systolic blood pressure >160 mmHg) that is likely to induce at TMA.
Study type	Allocation: non-randomized<br>Masking: open label<br>Primary purpose: Safety<br>Phase IIb
Date of first enrolment	November 2018
Target sample size	50: 30 withdrawal and 20 non-withdrawal
Recruitment status	Recruiting
Primary outcome(s)	To determine the safety of Eculizumab withdrawal in patients with aHUS
Key secondary outcomes	1. The effectiveness of a monitoring protocol to detect disease relapse following withdrawal of Eculizumab assessed by:<br>1.1. The proportion of patients who relapse and restart Eculizumab without the development of a TMA-related SAE<br>1.2. The time from the first clinical feature (symptom, positive urinalysis or laboratory result) of a relapse of TMA and the re-introduction of Eculizumab<br>This outcome is ongoing and not measured at any particular timepoint<br>2. The relapse rate after withdrawal of Eculizumab as determined by the proportion of patients who relapse after Eculizumab is withdrawn. This outcome is ongoing and not measured at any particular time point. A patient could relapse at any point in the 2 years participation period.<br>3. The proportion of patients, currently on long-term treatment with Eculizumab, who can be maintained off treatment. This outcome is measured at the end of the trial when all relapse data is collected. A patient could relapse at any point in the 2 years participation period.<br>4. The period from withdrawal to relapse in those patients who restart treatment. This outcome is measured at the end of the trial when all relapse data is collected.<br>5. The change in estimated GFR as calculated by the CKD-EPI or modified Schwartz equations over the course of the study from baseline (day 0) to

	end of the study. This outcome is calculated at the end of the trial when all GFR data is collected. GFR data is collected at all 34 visits.<br>6. Important clinical and laboratory indicators of imminent relapse by review of reported symptoms, physical signs, urinalysis and laboratory results prior to the diagnosis of a relapse. This outcome will be assessed at the end of the trial when all relapse data is collected. Those who have relapsed will have all data preceding relapse reviewed to establish a relapse profile.<br>7. The costs and health outcomes (measured in terms of adverse events and quality-adjusted life years [QALYs]) for patients on standard care (not withdrawing from Eculizumab treatment) over the two-year trial duration:<br>7.1. Healthcare Utilisation Questionnaires for non-withdrawal participants at Day 0, 14, 70,154, 252, 336, 504 and 672.<br>7.2. Adverse Event Assessment at every visit from Day 7 (32 visits) for withdrawal participants.<br>8. QALYs estimated from responses to the EQ-5D-5L, and SF-36 and determinants of QALYs/utilities over the 24-month follow-up period. at Day 0, 14, 70,154, 252, 336, 504 and 672.<br>9. Model-based estimate of the costs and health consequences, with results presented in terms of cost per QALY gained, over the estimated lifetime of patients withdrawing from treatment compared with standard care.

review only

Reporting checklist for protocol of a clinical trial.

Based on the SPIRIT guidelines.

Instructions to authors

Complete this checklist by entering the page numbers from your manuscript where readers will find each of the items listed below.

Your article may not currently address all the items on the checklist. Please modify your text to include the missing information. If you are certain that an item does not apply, please write "n/a" and provide a short explanation.

Upload your completed checklist as an extra file when you submit to a journal.

In your methods section, say that you used the SPIRITreporting guidelines, and cite them as:

Chan A-W, Tetzlaff JM, Gøtzsche PC, Altman DG, Mann H, Berlin J, Dickersin K, Hróbjartsson A, Schulz KF, Parulekar WR, Krleža-Jerić K, Laupacis A, Moher D. SPIRIT 2013 Explanation and Elaboration: Guidance for protocols of clinical trials. BMJ. 2013;346:e7586

		Reporting Item	Page Number
**Administrative information**			
Title	#1	Descriptive title identifying the study design, population, interventions, and, if applicable, trial acronym	1
Trial registration	#2a	Trial identifier and registry name. If not yet registered, name of intended registry	3
Trial registration: data set	#2b	All items from the World Health Organization Trial Registration Data Set	3, 4, 6, 7, 8, 9, 13, 16, 20
Protocol version	#3	Date and version identifier	3
Funding	#4	Sources and types of financial, material, and other support	16

For peer review only

Roles and responsibilities: contributorship	#5a	Names, affiliations, and roles of protocol contributors	1
Roles and responsibilities: sponsor contact information	#5b	Name and contact information for the trial sponsor	13 and 17
Roles and responsibilities: sponsor and funder	#5c	Role of study sponsor and funders, if any, in study design; collection, management, analysis, and interpretation of data; writing of the report; and the decision to submit the report for publication, including whether they will have ultimate authority over any of these activities	NA – sponsor and funder do not have involvement in these activities.
Roles and responsibilities: committees	#5d	Composition, roles, and responsibilities of the coordinating centre, steering committee, endpoint adjudication committee, data management team, and other individuals or groups overseeing the trial, if applicable (see Item 21a for data monitoring committee)	11, 12 and 13
**Introduction**			
Background and rationale	#6a	Description of research question and justification for undertaking the trial, including summary of relevant studies (published and unpublished) examining benefits and harms for each intervention	4
Background and rationale: choice of comparators	#6b	Explanation for choice of comparators	n/a – the trial withdraws patients from their current medication, no comparator required

For peer review only

Objectives	#7	Specific objectives or hypotheses	4 and 5
Trial design	#8	Description of trial design including type of trial (eg, parallel group, crossover, factorial, single group), allocation ratio, and framework (eg, superiority, equivalence, non-inferiority, exploratory)	4
**Methods: Participants, interventions, and outcomes**			
Study setting	#9	Description of study settings (eg, community clinic, academic hospital) and list of countries where data will be collected. Reference to where list of study sites can be obtained	6
Eligibility criteria	#10	Inclusion and exclusion criteria for participants. If applicable, eligibility criteria for study centres and individuals who will perform the interventions (eg, surgeons, psychotherapists)	6
Interventions: description	#11a	Interventions for each group with sufficient detail to allow replication, including how and when they will be administered	7
Interventions: modifications	#11b	Criteria for discontinuing or modifying allocated interventions for a given trial participant (eg, drug dose change in response to harms, participant request, or improving / worsening disease)	9

For peer review only

Interventions: adherence	#11c	Strategies to improve adherence to intervention protocols, and any procedures for monitoring adherence (eg, drug tablet return; laboratory tests)	9 – monitoring of home urinalysis
Interventions: concomitant care	#11d	Relevant concomitant care and interventions that are permitted or prohibited during the trial	7 – relating to meningococcal prophylaxis. No other concomitant medication requirements imposed by the trial
Outcomes	#12	Primary, secondary, and other outcomes, including the specific measurement variable (eg, systolic blood pressure), analysis metric (eg, change from baseline, final value, time to event), method of aggregation (eg, median, proportion), and time point for each outcome. Explanation of the clinical relevance of chosen efficacy and harm outcomes is strongly recommended	5
Participant timeline	#13	Time schedule of enrolment, interventions (including any run-ins and washouts), assessments, and visits for participants. A schematic diagram is highly recommended (see Figure)	7
Sample size	#14	Estimated number of participants needed to achieve study objectives and how it was determined, including clinical and statistical assumptions supporting any sample size calculations	6 and 10

For peer review only

Recruitment	#15	Strategies for achieving adequate participant enrolment to reach target sample size	6
**Methods: Assignment of interventions (for controlled trials)**			
Allocation: sequence generation	#16a	Method of generating the allocation sequence (eg, computer-generated random numbers), and list of any factors for stratification. To reduce predictability of a random sequence, details of any planned restriction (eg, blocking) should be provided in a separate document that is unavailable to those who enrol participants or assign interventions	NA- non-randomised trial
Allocation concealment mechanism	#16b	Mechanism of implementing the allocation sequence (eg, central telephone; sequentially numbered, opaque, sealed envelopes), describing any steps to conceal the sequence until interventions are assigned	NA- non-blinded
Allocation: implementation	#16c	Who will generate the allocation sequence, who will enrol participants, and who will assign participants to interventions	NA- non-randomised trial
Blinding (masking)	#17a	Who will be blinded after assignment to interventions (eg, trial participants, care providers, outcome assessors, data analysts), and how	N/A – non-blinded

For peer review only

Blinding (masking): emergency unblinding	#17b	If blinded, circumstances under which unblinding is permissible, and procedure for revealing a participant's allocated intervention during the trial	NA – non-blinded
**Methods: Data collection, management, and analysis**			
Data collection plan	#18a	Plans for assessment and collection of outcome, baseline, and other trial data, including any related processes to promote data quality (eg, duplicate measurements, training of assessors) and a description of study instruments (eg, questionnaires, laboratory tests) along with their reliability and validity, if known. Reference to where data collection forms can be found, if not in the protocol	7
Data collection plan: retention	#18b	Plans to promote participant retention and complete follow-up, including list of any outcome data to be collected for participants who discontinue or deviate from intervention protocols	9, 12 and 13
Data management	#19	Plans for data entry, coding, security, and storage, including any related processes to promote data quality (eg, double data entry; range checks for data values). Reference to where details of data management procedures can be found, if not in the protocol	13

For peer review only

Statistics: outcomes	#20a	Statistical methods for analysing primary and secondary outcomes. Reference to where other details of the statistical analysis plan can be found, if not in the protocol	10, 12 and 13
Statistics: additional analyses	#20b	Methods for any additional analyses (eg, subgroup and adjusted analyses)	12 and 13
Statistics: analysis population and missing data	#20c	Definition of analysis population relating to protocol non-adherence (eg, as randomised analysis), and any statistical methods to handle missing data (eg, multiple imputation)	12
**Methods: Monitoring**			
Data monitoring: formal committee	#21a	Composition of data monitoring committee (DMC); summary of its role and reporting structure; statement of whether it is independent from the sponsor and competing interests; and reference to where further details about its charter can be found, if not in the protocol. Alternatively, an explanation of why a DMC is not needed	11,12 and 13
Data monitoring: interim analysis	#21b	Description of any interim analyses and stopping guidelines, including who will have access to these interim results and make the final decision to terminate the trial	11 and 12
Harms	#22	Plans for collecting, assessing, reporting, and managing solicited	9 and 10

For peer review only

		and spontaneously reported adverse events and other unintended effects of trial interventions or trial conduct	
Auditing	#23	Frequency and procedures for auditing trial conduct, if any, and whether the process will be independent from investigators and the sponsor	13
**Ethics and dissemination**			
Research ethics approval	#24	Plans for seeking research ethics committee / institutional review board (REC / IRB) approval	14
Protocol amendments	#25	Plans for communicating important protocol modifications (eg, changes to eligibility criteria, outcomes, analyses) to relevant parties (eg, investigators, REC / IRBs, trial participants, trial registries, journals, regulators)	13
Consent or assent	#26a	Who will obtain informed consent or assent from potential trial participants or authorised surrogates, and how (see Item 32)	7
Consent or assent: ancillary studies	#26b	Additional consent provisions for collection and use of participant data and biological specimens in ancillary studies, if applicable	7
Confidentiality	#27	How personal information about potential and enrolled participants will be collected, shared, and maintained in order to protect confidentiality before, during, and after the trial	13

For peer review only

Declaration of interests	#28	Financial and other competing interests for principal investigators for the overall trial and each study site	16
Data access	#29	Statement of who will have access to the final trial dataset, and disclosure of contractual agreements that limit such access for investigators	13
Ancillary and post trial care	#30	Provisions, if any, for ancillary and post-trial care, and for compensation to those who suffer harm from trial participation	7 – post trial safety monitoring
Dissemination policy: trial results	#31a	Plans for investigators and sponsor to communicate trial results to participants, healthcare professionals, the public, and other relevant groups (eg, via publication, reporting in results databases, or other data sharing arrangements), including any publication restrictions	14
Dissemination policy: authorship	#31b	Authorship eligibility guidelines and any intended use of professional writers	16
Dissemination policy: reproducible research	#31c	Plans, if any, for granting public access to the full protocol, participant-level dataset, and statistical code	13
**Appendices**			
Informed consent materials	#32	Model consent form and other related documentation given to participants and authorised surrogates	N/A – not included but can be requested from the Trial Manager Sarah Dunn sarah.dunn2@newcastle.ac.uk

For peer review only

Biological specimens	#33	Plans for collection, laboratory evaluation, and storage of biological specimens for genetic or molecular analysis in the current trial and for future use in ancillary studies, if applicable	N/A – not included, laboratory manual can be requested from the Trial Manager Sarah Dunn sarah.dunn2@newcastle.ac.uk

None The SPIRIT Explanation and Elaboration paper is distributed under the terms of the Creative Commons Attribution License CC-BY-NC. This checklist can be completed online using https://www.goodreports.org/, a tool made by the EQUATOR Network in collaboration with Penelope.ai

For peer review only

Protocol for a multicentre, open label, prospective, single arm study of the safety and impact of eculizumab withdrawal in patients with atypical haemolytic uraemic syndrome

Journal:	*BMJ Open*
Manuscript ID	bmjopen-2021-054536.R2
Article Type:	Protocol
Date Submitted by the Author:	16-Aug-2022
Complete List of Authors:	Dunn, Sarah; Newcastle University, Newcastle Clinical Trials Unit<br>Brocklebank, Victoria; Newcastle University, National Renal Complement Therapeutics Centre<br>Bryant, Andrew; Newcastle University, Population Health Sciences Institute<br>Carnell, Sonya; Newcastle University, Newcastle Clinical Trials Unit<br>Chadwick, Thomas; Newcastle University, Population Health Sciences Institute<br>Johnson, Sally; Newcastle Upon Tyne Hospitals NHS Foundation Trust, Great North Children's Hospital ; Newcastle University, National Renal Complement Therapeutics Centre<br>Kavanagh, David; Newcastle University, Translational and Clinical Research Institute ; Newcastle University, National Renal Complement Therapeutics Centre<br>Lecouturier, Jan; Newcastle University, Population Health Sciences Institute<br>Malina, Michal; Newcastle Upon Tyne Hospitals NHS Foundation Trust, Great North Children's Hospital; Newcastle University, National Renal Complement Therapeutics Centre<br>Moloney, Eoin; Optimax Access LTD, Health Economics<br>Oluboyede , Yemi; Newcastle University, Population Health Sciences Institute<br>Weetman, Christopher; Newcastle University, Newcastle Clinical Trials Unit<br>Wong, Edwin; Newcastle University, Translational and Clinical Research Institute ; Newcastle University, National Renal Complement Therapeutics Centre<br>Woodward, Len; aHUS alliance Global Action<br>Sheerin, Neil; Newcastle University, Translational and Clinical Research Institute ; Newcastle University, National Renal Complement Therapeutics Centre
**Primary Subject Heading**:	Renal medicine
Secondary Subject Heading:	Qualitative research, Health economics
Keywords:	NEPHROLOGY, HEALTH ECONOMICS, QUALITATIVE RESEARCH

SCHOLARONE™
Manuscripts

Protocol for a multicentre, open label, prospective, single arm study of the safety and impact of eculizumab withdrawal in patients with atypical haemolytic uraemic syndrome

Details of corresponding author

Full name	Postal address	Email	ORCHID ID
Ms Sarah Dunn, MSc	Newcastle Clinical Trials Unit, Newcastle University, 1-4 Claremont Terrace, Newcastle Upon Tyne, England, NE2 4AE	Sarah.dunn2@ncl.ac.uk	0000-0002-1901-3931

Details of Co-authors

Full name	Department	Institution	City	Country	Email
Dr Victoria Brocklebank, MBBS	National Renal Complement Therapeutics Centre	Newcastle University	Newcastle Upon Tyne	England	Victoria.Brocklebank@newcastle.ac.uk
Mr Andrew Bryant, MSc	Population Health Sciences	Newcastle University	Newcastle Upon Tyne	England	andy.bryant@newcastle.ac.uk
Dr Sonya Carnell PhD	Newcastle Clinical Trials Unit	Newcastle University	Newcastle Upon Tyne	England	sonya_carnell@yahoo.com
Dr Thomas J Chadwick PhD	Population Health Sciences	Newcastle University	Newcastle Upon Tyne	England	thomas.chadwick@newcastle.ac.uk
Dr Sally Johnson MB ChB MRCPCH, PhD	Great North Children's Hospital, Translational and Clinical Research Institute and National Renal Complement Therapeutics Centre	Newcastle Upon Tyne Hospitals NHS Foundation Trust and Newcastle University	Newcastle Upon Tyne	England	sally.johnson15@nhs.net
Professor David Kavanagh, MB, ChB, MRCP, PhD, FRCP	Translational and Clinical Research Institute and National Renal Complement Therapeutics Centre	Newcastle University	Newcastle Upon Tyne	England	David.Kavanagh@newcastle.ac.uk
Ms Jan Lecouturier, MSc	Population Health Sciences	Newcastle University	Newcastle Upon Tyne	England	jan.lecouturier@newcastle.ac.uk

For peer review only

Dr Michal Malina, MD, PhD, FRCPCH	Great North Children's Hospital and National Renal Complement Therapeutics Centre	Newcastle Upon Tyne Hospitals NHS Foundation Trust And Newcastle University	Newcastle Upon Tyne	England	michal.malina@nhs.net
Mr Eoin Moloney, MSc	Health Economics	Optimax Access LTD	Newcastle Upon Tyne	England	eoin.moloney@optimaxaccess.com
Dr Yemi Oluboyede PhD	Population Health Sciences	Newcastle University	Newcastle Upon Tyne	England	yemi.oluboyede@newcastle.ac.uk
Mr Christopher Weetman, BSc	Newcastle Clinical Trials Unit	Newcastle University	Newcastle Upon Tyne	England	christopher.weetman@newcastle.ac.uk
Dr Edwin Kwan Soon Wong, MBChB, PhD MRCP	Translational and Clinical Research Institute and National Renal Complement Therapeutics Centre	Newcastle University	Newcastle Upon Tyne	England	edwin.wong@newcastle.ac.uk
Mr Len Woodward	N/A	aHUS alliance Global Action	Newcastle Upon Tyne	England	len.woodward@ntlworld.com
Professor Neil Sheerin, MBBS, PhD, MRCP	Translational and Clinical Research Institute and National Renal Complement Therapeutics Centre	Newcastle University	Newcastle Upon Tyne	England	Neil.Sheerin@newcastle.ac.uk

Word Count: 4653

For peer review only

ABSTRACT

Introduction

Atypical haemolytic uraemic syndrome (aHUS) is a rare, life threatening disease caused by excessive activation of part of the immune system called complement. Eculizumab is an effective treatment, controlling aHUS in 90% of patients. Due to the risk of relapse, lifelong treatment is currently recommended. Eculizumab treatment is not without problems, foremost being the risk of severe meningococcal infection, the burden of bi-weekly intravenous injections and the high cost.

This paper describes the design of the Stopping Eculizumab Treatment Safely in aHUS (SETS aHUS) trial that aims to establish whether a safety monitoring protocol, including the reintroduction of Eculizumab for those who relapse, could be a safe, alternative treatment strategy for patients with aHUS.

Methods and analysis

This is a multi-centre, non-randomised, open label study of Eculizumab withdrawal with continuous monitoring of thrombotic microangiopathy (TMA) related serious adverse events using the Bayes factor single arm design. 30 patients will be recruited to withdraw from Eculizumab and have regular blood and urine tests for 24 months, to monitor for disease activity. If relapse occurs, treatment will be restarted within 24 hours of presentation. 20 patients will remain on treatment and complete health economic questionnaires only. An embedded qualitative study will explore the views of participants.

Ethics and dissemination

A favourable ethical opinion and approval was obtained from the North East - Tyne & Wear South Research Ethics Committee. Outcomes will be disseminated via peer-reviewed articles and conference presentations.

Trial registration

EudraCT Number: 2017-003916-37

ISRCTN number: ISRCTN17503205

Date of Registration: 20 April 2018

Keywords

atypical Haemolytic Uraemic Syndrome, qualitative research, Complement, Eculizumab

Article summary

Strengths and limitations of this study

- This is the first UK trial to evaluate the safety of eculizumab withdrawal in patient with aHUS.

For peer review only

- This trial fulfils the NICE recommendation that a research programme, with robust methods, should be carried out to evaluate when stopping Eculizumab treatment or dose adjustment might occur.
- Clinical experience suggests if relapse occurs this will likely happen in the first 12 months of withdrawal however, this trial follows patients up for 24 months to capture those patients who may relapse after the 12-month point.
- The small number of aHUS patients on treatment in the UK is insufficient to conduct a standard parallel group, randomised controlled trial.
- COVID-19 has had an impact on recruitment.

Trial Status

This manuscript is based upon trial protocol version 7.0 dated 14th January 2021. The first patient was recruited in November 2018, recruitment ended on 31st January 2022 and planned last patient visit is November 2023.

For peer review only

INTRODUCTION

Atypical Haemolytic Uraemic Syndrome (aHUS) is a severe, life-threatening disease characterised by thrombocytopenia, microangiopathic haemolytic anaemia and Acute Kidney Injury (AKI), and other organ involvement. Historically it is associated with a poor prognosis, with 50% of patients developing end stage kidney disease or dying in the first year after presentation [1] and a high risk of disease recurrence after kidney transplantation [2]. Prior to 2011, treatment options were limited and relied on plasma infusion or exchange, but in many cases this treatment failed to influence the course of disease [1] and was itself associated with significant morbidity and mortality [3]. In the UK, the incidence of aHUS is 0.4-0.5 cases per million per year [4].

The complement system is part of the innate immune system and in healthy individuals is tightly regulated to prevent excessive activation. In 60-70% of patients with aHUS, a genetic variant or autoantibody increasing complement activation can be identified [10]. In these patients, excessive activation of complement leads to endothelial injury and thrombus formation. The underlying genetic variant that predisposes to disease has an influence on the severity of disease and the likelihood of recurrent disease developing after transplantation [5].

Eculizumab is a humanized monoclonal antibody that inhibits the function of C5, an important protein involved in complement activation. Two uncontrolled, open label trials involving 36 adult and adolescent patients demonstrated the efficacy of Eculizumab treatment for aHUS over a 26 week period [6]. Additional prospective studies in children [7] and adults [8] confirmed efficacy. Follow-up of the original cohort suggests that treatment for 2 years is associated with good, longer-term clinical outcomes [9]. On the basis of the initial trial results, Eculizumab was approved for the treatment of aHUS by the European Medicines Agency (EMA) [10] and U.S. Food & Drug Administration (FDA) [11] in 2011. The National Institute for Health and Care Excellence (NICE) published its evaluation in 2015, recommending that Eculizumab should be used for the treatment of aHUS [12]. A recommendation in the NICE evaluation was that funding was on the condition that there was a 'research programme with robust methods to evaluate when stopping treatment or dose adjustment might occur'.

Complement is part of the immune system, therefore, treatment with Eculizumab is immunosuppressive, in particular, increasing the risk of *Neisseria meningitidis* infection (1-2000 fold)[13, 14]. All patients are vaccinated against meningococcal infection before starting Eculizumab and in the UK continuous prophylactic antibiotics are recommended. Despite these recommendations, there have been 6 cases of meningococcal infection in the UK in patients on Eculizumab treatment for aHUS. We are also aware of uncommon infections occurring in this group (enteroviral pneumonitis and *Herpes simplex* meningitis) but whether these are attributable to Eculizumab treatment is unclear.

Although there have been reports of patients relapsing after the withdrawal of treatment [15], there is increasing evidence that continuous treatment is not required for all patients. From experience prior to the introduction of Eculizumab, the risk of relapse is greatest in the period immediately after first presentation with 82% of relapses in adults, and 57% of relapses in children occurring within the first year after disease onset.

For peer review only - http://bmjopen.bmj.com/site/about/guidelines.xhtml

Beyond the first year, only a further 20% of patients will relapse in the subsequent 5-10 years [1]. Therefore, a proportion of patients will not relapse after their initial presentation and will be on Eculizumab unnecessarily. In addition, with monitoring for relapse and early reintroduction of treatment, complications from relapse can be avoided [16-18].

In this trial we will test the safety of eculizumab withdrawal using a Bayesian trial design. The efficacy of self-monitoring will also be tested, and we will explore patients' and parents/legal guardians' views on how treatment and monitoring of disease can be delivered most effectively.

METHODS AND ANALYSIS

Objectives and outcome measures

Primary

The primary clinical objective is to determine the safety of Eculizumab withdrawal in patients with aHUS, measured by the number of patients with a Thrombotic microangiopathy (TMA) related Serious Adverse Event (SAE) defined as any of the following:

- Irreversible (>3 months) reduction in estimated glomerular filtration rate (eGFR) -not attributable to another cause:

In adults

- by ≥20% if the screening eGFR is <90mls/min/1.73m2,
- by >20% to a level <90mls/min/1.73m2 if the screening eGFR is >90mls/min/1.73m2.

In children

- by ≥20% if the screening eGFR is <75mls/min/1.73m2,
- by >20% to a level <75mls/min/1.73m2 if the screening eGFR is >75mls/min/1.73m2.

- An episode of AKI attributed to a TMA that requires renal replacement therapy.
- A non-renal manifestation of a TMA that require hospitalisation, cause irreversible organ damage or death.

Secondary

Clinical

1. Measure the effectiveness of a monitoring protocol to detect disease relapse following withdrawal of Eculizumab as assessed by:

- The proportion of patients who relapse and restart Eculizumab without the development of a TMA-related SAE.
- The time from the first clinical feature (symptom, positive urinalysis or laboratory result) of a relapse of TMA and the re-introduction of Eculizumab.

For peer review only - http://bmjopen.bmj.com/site/about/guidelines.xhtml

2. The relapse rate after withdrawal of Eculizumab as determined by the proportion of patients who relapse after Eculizumab is withdrawn.

3. The proportion of patients, currently on long-term treatment with Eculizumab, who can be maintained off treatment.

4. The period from withdrawal to relapse in those patients who restart treatment.

5. The change in estimated GFR as calculated by the CKD-EPI or modified Schwartz equations over the course of the study from baseline (day 0) to end of the study.

6. Important clinical and laboratory indicators of imminent relapse by review of reported symptoms, physical signs, urinalysis and laboratory results prior to the diagnosis of a relapse.

Health Economic

7. The costs and health outcomes (measured in terms of adverse events and quality-adjusted life years [QALYs]) for patients on standard care (not withdrawing from Eculizumab treatment) over the two-year trial duration:

- Healthcare Utilisation Questionnaires for non-withdrawal participants at Day 0, 14, 70,154, 252, 336, 504 and 672.
- Adverse Event Assessment at every visit from Day 7 (32 visits) for withdrawal participants.

8. QALYs estimated from responses to the EQ-5D-5L, and SF-36 and determinants of QALYs/utilities over the 24-month follow-up period. at Day 0, 14, 70,154, 252, 336, 504 and 672.

9. Model-based estimate of the costs and health consequences, with results presented in terms of cost per QALY gained, over the estimated lifetime of patients withdrawing from treatment compared with standard care.

Trial Design

This is a multi-centre, non-randomised, open label study of Eculizumab withdrawal with continuous monitoring of TMA related serious adverse events using the Bayes factor single arm design of Johnson and Cook [19]. The patients will self-select whether they wish to withdraw from Eculizumab and carry out the monitoring protocol or remain on treatment and be part of the Health Economic analysis-only. An economic analysis, informed by the results of this trial, will determine whether Eculizumab withdrawal, substituting treatment with a protocolised surveillance and treatment reintroduction strategy, is cost-effective. The patient visit schedule for the withdrawal cohort is shown in Figure 1, and the Health Economic cohort in Figure 2.

Trial setting

This multi-centre trial will be carried out in up to 20 adult and paediatric Renal units (secondary and tertiary care) in the UK who are using Eculizumab to treat patients with aHUS.

Eligibility

All patients must fulfil the following inclusion criteria in order to be eligible for the trial:

For peer review only - http://bmjopen.bmj.com/site/about/guidelines.xhtml

- Age ≥2+ years of age,
- On Eculizumab treatment for at least 6 months,
- In remission with no evidence of ongoing microangiopathic haemolytic anaemia (MAHA) activity at screening defined by:
  - Platelet count > lower limit of normal as determined by local reference range,
  - Lactate Dehydrogenase (LDH) <x2 upper limit of normal as determined by local lab reference ranges,
- Normal renal function or Chronic Kidney Disease (CKD) stages 1-3 (eGFR >30mls/min/1.73m^2),
- Absence of decline of renal function confirmed by review of available assessments of renal function for the preceding 6 months by the Chief Investigator (CI) and clinical members of the Trial Management Group (TMG).

The following inclusion criteria must be met only by those wishing to participate in the withdrawal component of the trial:

- Willing to attend for safety monitoring assessments,
- Willing to travel only to countries that can supply Eculizumab (to be confirmed with co-ordinating centre prior to travel),
- Able to perform or parent/guardian to perform and record self-monitoring urinalysis,
- Sexually active female patients must have a negative pregnancy test at screening and be using an effective contraception for the duration of the study.

OR

- fulfil one of the following criteria:
  - Be post-menopausal,
  - Have undergone surgical sterilisation.

The following exclusion criteria is applicable to all patients wishing to participate in the trial:

- Severe non-renal disease manifestations at initial presentation with aHUS, which in the opinion of the Chief Investigator and/or the clinical members of the TMG makes the risk of treatment withdrawal unacceptable,
- Current or planned pregnancy within the study duration,
- Unable to give informed consent or assent, or unable to obtain parent/guardian consent if under 16 years of age,
- Current participation in another clinical trial (not including participation in aHUS registries),
- Severe, uncontrolled hypertension (systolic blood pressure >160 mmHg) that is likely to induce at TMA.

The following exclusion criteria is applicable only to those wishing to participate in the withdrawal component of the trial:

- Loss of a previous transplant kidney to recurrent aHUS,
- Transplant recipient with a pathogenic mutation in *C3*, *CFH* or *CFB*,

For peer review only

For peer review only - http://bmjopen.bmj.com/site/about/guidelines.xhtml

- Haematuria rating of 3+.

Screening and Recruitment

30 patients will be recruited to withdraw from Eculizumab treatment, and 20 patients will be recruited who will remain on treatment and complete the Health Economic questionnaires only.

Patients with a diagnosis of aHUS receiving Eculizumab [4] to treat disease in native or transplanted kidneys will be identified by the National aHUS Service, which maintains a list of patients who fulfil these criteria as part of the NHS England commissioned service. Those patients who meet the genetic eligibility criteria will be highlighted to site teams who will carry out formal screening assessments. A physical examination and vital signs will be performed, and routine safety laboratory tests will be reviewed to ensure that a patient fulfils all eligibility criteria for entry into the study. Female participants withdrawing from treatment, who are of childbearing age and sexually active, will be required to have a negative pregnancy test prior to treatment withdrawal. Participants will also consent to have samples taken for exploratory analysis and storage at Newcastle University biobank for use in future research.

Consent will be sought from the parents/legal guardian on behalf of patients under the age of 16. Assent will be taken from those patients under 16 years old, as appropriate (supplement 1). No trial related procedures will be carried out prior to consent.

Intervention

Patients who consent to withdraw from Eculizumab will receive their last dose of Eculizumab during visit 1 (classed as day -14 prior to withdrawal).

Visit Details and Assessments

Baseline Assessments & Data collection for withdrawal cohort (Visit 2, Day 0 +/- 2 days)

Study day 0 will be the day that the participants would usually receive their next dose of Eculizumab, based on standard dosing schedules (+/-2 days). The Eculizumab will not be administered however, meningococcal prophylaxis will be continued for a further 2 weeks after day 0.

At day 0 of the study (Visit 2), participants will undergo the following assessments:
Vital signs (temperature, pulse and blood pressure), Height & weight, renal function (creatinine and eGFR), Urinalysis and urine protein/creatinine ratio, haemolysis markers including platelet count, haemoglobin, LDH, Electrolyte Profile, Liver Function (Bilirubin, ALT/AST, ALP, LDH, serum calcium, phosphate, albumin & total protein), haptoglobin (if available) and blood film, concomitant medication review, health-related quality of life questionnaires (EQ-5D-5L and SF-36) and health care utilisation questionnaire. A biomarker and complement activation sample to identify predictors of relapse (for example soluble C5b-9) is also taken and stored at site before transfer to Newcastle University.

For peer review only - http://bmjopen.bmj.com/site/about/guidelines.xhtml

Study Visit Assessments & Data collection for withdrawal cohort (Visits 3-34)

Participants will be assessed regularly for evidence of disease relapse for the 2-year duration of the study. The participants will attend a total of 32 safety monitoring visits over the 2-year withdrawal follow-up period.

Trial participants will be reviewed at the trial site weekly (+/- 2 days) for the first month, then alternate weeks (+/-2 days) until month 6, then monthly (+/- 7 days) thereafter until the end of the trial period (month 24). At each study visit, the participants will undergo the monitoring assessments as detailed in Figure 1.
Paediatric participants must have their weight recorded at every visit for calculation of eGFR. At the end of the trial, the level of safety monitoring for those patients who remain off treatment and disease free will be decided by their local clinical care team in discussion with the National aHUS Service.

Due to the COVID-19 pandemic, participants may be unable to attend their scheduled follow up visits or may be attending a local hospital to have safety bloods taken. If the participants are unable to attend site due to self-isolation or underlying health issues; where possible, a remote, follow-up call will be carried out by a member of the local research team. Participants will be asked to report changes to their concomitant medications, any adverse events experienced since their previous follow-up and the results of their home urinalysis tests.

Health Economic Assessments

Participants, or their parent/legal guardian, in both withdrawal and Health Economics cohorts will complete the EQ-5D-5L (proxy version if patient < 12 years), SF36 (parent/legal guardian completes if patient <14 years) and a health care utilisation questionnaire at 8 timepoints. A time and travel questionnaire is completed at 1 timepoint, as detailed in Figures 1 and 2.

Self-monitored Urinalysis

Withdrawal participants, or their parent/legal guardian, will be trained to perform and understand the results of home urinalysis. Urinalysis, for the presence of haematuria or haemoglobinuria as an indicator of intravascular haemolysis and therefore disease activity, will then be performed daily by the participant or parent/legal guardian for the first month and then three times per week for the remainder of the study period. The results will be recorded in a participant diary and will be reviewed at each study visit. Participants or their parent/legal guardian will report any significant change in urinalysis, not related to menstruation, using their own baseline result to guide them in relation to the thresholds as detailed in *Table 1*.

Table 1. Home Urinalysis result thresholds

Baseline Haematuria result	Haematuria result threshold (not related to menstruation)
Neg/Trace	++ on any occasion OR + on any two occasions 24 hours apart
+	+++ on any occasion OR ++ on any two occasions 24 hours apart

For peer review only - http://bmjopen.bmj.com/site/about/guidelines.xhtml

++	+++ on any occasion

If the threshold criteria are met, participants or their parent/legal guardian will contact their treatment site immediately to arrange an unscheduled visit to assess disease activity as outlined in *Figure 1*.

Trial Withdrawal

Participants will have the right to withdraw from the study at any time for any reason, and without giving a reason. The investigator will also have the right to withdraw participants from the study if she/he judges this to be in their best interest. Those participants who have been withdrawn from treatment can request to restart treatment, even if they have not relapsed. Data and blood samples provided by the participant up until the point of withdrawal will be included in analysis, unless they specifically request to have this removed. Participants who withdraw from the trial will not be replaced.

Change in Health Status

Participants will be advised to report any significant change in health status to the responsible site or local health care provider. Participants will be provided with a participant identification card to present to attending medical staff with details of the study, tests required and study centre and National aHUS Service contact details (Supplement 2). Sites will notify the participants' General Practitioner of their involvement in the study and inform them of the required action to be taken in the case of suspected relapse. Criteria for a diagnosis of aHUS relapse are shown in Figure 3.

If there is a clinical suspicion of disease activity, formal assessment will occur as outlined in *Figure 1*, Unscheduled visit column.

Any other adverse events that could represent a relapse will be discussed with the Investigators and/or the aHUS National Service. A decision to restart will be made according to current Service procedures.

Relapse Management

When a relapse is diagnosed, participants will restart Eculizumab treatment within 24 hours of presentation provided there is no evidence of an active infection that would be a contra-indication to treatment at the recommended dose of 900mg weekly for the first 4 weeks then 1200mg every two weeks thereafter (or age adjusted dose and regime). TMA activity will be monitored (platelet count, LDH) as recommended by attending clinician until haematological remission is achieved as defined in Figure 4.

Participants who relapse and require re-introduction of Eculizumab treatment will remain on treatment in study under follow up for the full 2 years of the study. Home urinalysis will not be required after re-introduction of Eculizumab treatment.

For peer review only - http://bmjopen.bmj.com/site/about/guidelines.xhtml

Participants will consent to travel to only those countries where Eculizumab is available. If a participant relapses while they are travelling outside of the country, the National aHUS Service will make arrangements with the destination country to access and fund Eculizumab if required, with arrangements from the commissioning authority.

Embedded qualitative study

In-depth one-to-one telephone interviews will be conducted following a topic guide developed with the input of the research team, including PPI. The intention is to keep interviews very broad to ensure we capture the full experience of interviewees.

Up to 30 patients who withdraw from Eculizumab and up to 20 patients who decline to withdraw will be approached to participate. Up to 20 patients who withdraw will be re-interviewed at the end of the withdrawal period (24 months later) to explore their views of the monitoring protocol. This group will be asked at the first interview if they agree to be contacted again towards the end of the study for a follow up interview. Where possible, any patients who relapse and go back onto treatment will also be interviewed. Consent will be recorded at the time of the interview. Interviews will be digitally recorded with the permission of the interviewee, transcribed verbatim and anonymised.

Safety reporting

All Adverse Events (AEs) occurring from the point of withdrawal (day 0) to end of study participation will be recorded. SAEs occurring from the point of withdrawal (day 0) must be reported to The Newcastle Clinical Trials Unit (NCTU) within 24 hours of the site becoming aware of the event. Serious Adverse Reactions (SARs) and Suspected Unexpected Serious Adverse Reactions (SUSARs) are reportable only for those participants who have Eculizumab treatment re-initiated during their participation in the trial. The assessment of expectedness will be performed by the PI at site against the approved Reference Safety Information (RSI) for the trial (Section 4.8 of the Soliris SmPC).

Statistical Analysis

Sample size

A maximum of 30 patients will be recruited to the withdrawal component; this is judged to be reasonable given the rare nature of the disease. The specifics of this sample size are intrinsically linked to the Bayes factor single arm binary design employed to analyse the primary outcome measure. There is no allowance for loss to follow-up as this patient group is already subject to a high degree of clinical follow-up and death is defined as one of the serious events under consideration. 20 patients will be recruited to the non-withdrawal arm as a comparator group for health economic analysis only.

Analysis

For peer review only - http://bmjopen.bmj.com/site/about/guidelines.xhtml

This is a single arm, open label trial with the primary endpoint being a binary response (the presence/absence of a primary outcome event within the follow-up period). We will compare the rate of TMA-related serious adverse events (primary outcomes) following the withdrawal of medication to that of treatment-related serious adverse events, expected under standard care.

The Bayes factor single arm binary model [19] will be used to monitor the trial. Based on historical data, the event rate (treatment-related serious adverse events in 100 patients over a 2 year period) for the standard of care is 0.06, and we expect that withdrawal of the treatment would give a rate of 0.12. This choice of rate has been informed in discussion with patients. Using this Bayesian hypothesis test-based design, we assume the rate is 0.06 under the null, and 0.12 under the alternative hypothesis.

We assume that the sample distribution of number of responses follows a binomial distribution and use an inverse moment prior for response under the alternative hypothesis.

Stopping Rules

A minimum of 5 patients will be enrolled before applying the stopping rules, and the cohort size for monitoring is 5 patients. The Data Monitoring Committee (DMC) can request earlier review if adverse events are reported before this point.

We implement two stopping rules:

(1) We will stop the trial for superiority (there being fewer TMA related serious adverse events on the intervention than would be expected under standard of care) if the posterior probability of the alternative hypothesis is less than 0.05, i.e. Pr(H1|Data) < 0.05;

(2) We will stop the trial for inferiority if the posterior probability of the alternative hypothesis is greater than 0.80, i.e., Pr(H1|Data) > 0.80.

Operating Characteristics and Stopping Boundaries

The operating characteristics (Table 2) and stopping boundaries (Table 3) were produced using the M. D. Anderson Cancer Center Department of Biostatistics software BayesFactorBinary, version 1.0 [20]

Table 2. Operating Characteristics

Scenario	True rate of treatment related serious adverse events	Probability of Stopping for Inferiority	Probability of Stopping for Superiority	Average number of patients treated (Percentiles: 10%, 25%, 50%, 75%, 90%)
1	0.06	0.096	0	28.44 (30, 30, 30, 30, 30)
2	0.12	0.443	0	23.48 (5, 15, 30, 30, 30)
3	0.18	0.753	0	17.76 (5, 10, 15, 30, 30)
4	0.24	0.928	0	13.72 (5, 5, 15, 15, 30)
5	0.30	0.982	0	10.52 (5, 5, 10, 15, 20)

For peer review only

For peer review only - http://bmjopen.bmj.com/site/about/guidelines.xhtml

If the true rate is 0.06 (Scenario 1, null hypothesis), the trial will stop with probabilities of 0.096 and 0 in favour of the alternative and null hypotheses, respectively. The average number of patients (10%, 90% percentiles) is 28.44 (30, 30). If the true rate is 0.12 (Scenario 2, alternative hypothesis), the trial will stop with probabilities of 0.443 and 0 in favour of the alternative and null hypotheses, respectively. The average number of patients (10%, 90%) is 23.48 (5, 30).

Table 3 – SETS aHUS Trial Stopping Boundaries

Number of patients (in complete cohorts of 5)	**Stop** the trial for Superiority if there are this many TMA **Events** (inclusive)	**Continue** the trial if there are this many TMA **Events** (inclusive)	**Stop** the trial for Inferiority if there are this many TMA **Events** (inclusive)
5	Never stop for superiority with this many patients	0-1	2-5
10 or 15	Never stop for superiority with this many patients	0-2	3-15
20	Never stop for superiority with this many patients	0-3	4-20
25 or 30	Never stop for superiority with this many patients	0-4 (The trial always stops at 30 patients, which is the maximum)	5-30

The study will stop for inferiority with 2 TMA related serious adverse events in the first cohort of 5 participants. Subsequently, the study would stop if 3 or more TMA related serious adverse events are observed in the first 15 participants, 4 or more in the first 20 participants, and 5 or more in the whole study population. We are well placed to respond to any negative safety signal.

1000 repetitions were used in the software simulation. Calculations with different numbers of repetitions resulted in unchanged stopping boundaries with only marginal changes to the operating characteristics.

There may be differing risk of relapse according to disease aetiology. However, the available numbers do not allow for risk strata to be monitored separately. The DMC will consider this within their remit.

In addition to this ongoing analysis, at the end of the study, data will also be reported descriptively, together with the number of patients recruited. Descriptive statistics reported will be selected as appropriate to the specific outcome measure. For proportion outcomes, the number of patients recording the event will also be reported.

Due to the sample size, no comparative statistical methods will be applied. There will be no imputation of missing data and a complete case analysis will be undertaken.

Subgroup Analyses

Except for the analysis of the primary outcome on an ongoing basis, the analyses described above may be reported separately for different genetic groups or risk strata.

Health Economic analysis

For peer review only - http://bmjopen.bmj.com/site/about/guidelines.xhtml

Within-trial assessments of costs and outcomes

Costs and health outcomes (measured in terms of resource use of primary and secondary health care NHS services and QALYs) associated with Eculizumab withdrawal (30 participants), compared with standard care (20 participants), will be assessed over the 24-month follow-up period. Information on costs and health outcomes will be recorded for each individual involved in both treatment groups. Data derived from the within-trial analysis will be assessed to understand the key determinants of differences in costs and outcomes between the two patient groups. Data will then be used to parameterise the lifetime economic model (combined with data from the literature).

Assessment of cost-effectiveness

An economic decision model will be developed to assess the cost-effectiveness of the alternative treatment options under evaluation. Costs and health consequences, measured in terms of QALYs, associated with Eculizumab withdrawal, and a policy of monitoring following withdrawal, and standard care, beyond the two-year timeframe of the trial will be captured. We propose to conduct a cost-utility analysis, with results presented in terms of incremental cost per QALY gained.

Qualitative analysis

We will take an inductive approach to data collection and analysis. This means there is no a priori theory; themes, concepts and theories will be elicited from the interview data when it is analysed and drawing upon relevant literature, PPI, and experts in the study team. Data will be analysed thematically using a constant comparative method. This entails a process of familiarisation with the data and then the development of a thematic framework. A small number of transcripts will be coded, and the framework amended accordingly. A second level analysis will be conducted using a constant comparative method. This involves a process of comparing and contrasting themes elicited from the data, within and across interviews [21]. NVivo will be used as a data management tool.

Trial Management and monitoring

This trial is sponsored by the Newcastle Upon Tyne Hospitals NHS Foundation Trust. The trial will be co-ordinated by a TMG that will include those individuals responsible for the day-to-day management of the trial. A Trial Steering Committee (TSC) made up of independent clinical and lay members will provide overall supervision of the trial. A Data Monitoring Committee (DMC) composed of independent clinicians and statistician will undertake independent review and monitor efficacy and safety endpoints. The trial was prospectively registered on the International Standard Registered Clinical/soCial sTudy Number (ISRCTN) registry and the European Union Drug Regulating Authorities Clinical Trials Database (EudraCT) (supplement 3). The Newcastle Clinical Trials Unit (NCTU) will be responsible for communicating protocol amendments to participating sites and carrying out central, remote, and on-site monitoring.

For peer review only - http://bmjopen.bmj.com/site/about/guidelines.xhtml

Confidentiality and data handling

Personal data will be regarded as strictly confidential. To preserve anonymity, a unique participant ID will be assigned to each participant at consent. Only the clinical team at the participating sites will have access to key data which links study identifiers to individual datasets. All study records and Investigator Site Files will be kept at site in a locked filing cabinet with restricted access.

Written consent will be sought from participants or legal guardians, if patient is under the age of 16 years, to allow access to their hospital records.

Data is recorded by authorised staff and stored in a secure web-based electronic data capture system (MACRO) designed and maintained by NCTU hosted on secure servers at Rackspace within the UK. Analysis of the data will be undertaken by the Newcastle University trial statisticians. Anonymised data from this trial may be available to the scientific community subject to regulatory and ethics approval. Requests for data should be directed to the corresponding author. All study data will be archived for 5 years.

Patient and public involvement (PPI)

A PPI representative sits on the Trial Management Group, was involved in protocol and study document development, and is involved in ongoing trial management discussions. We also have an aHUS patient as an independent member of the Trial Steering Committee.

ETHICS AND DISSEMINATION

A favourable ethical opinion and approval was obtained from the North East - Tyne & Wear South Research Ethics Committee in April 2018. Written informed consent will be obtained from all participants prior to their involvement in the trial. The results of the study will be submitted to peer-reviewed journals, presented at conferences and on the trial website.

DISCUSSION

This study will determine whether it is safe to withdraw Eculizumab using a trial methodology designed to detect an excess of adverse outcomes following withdrawal (primary endpoint). The study will also estimate the proportion of patients with aHUS that can be maintained off Eculizumab and test a system for surveillance to identify relapse early (secondary endpoints). This will allow a cost-utility analysis to be conducted, exploring the impact of treatment withdrawal [22]. This carefully monitored patient group will allow us to determine how early sub-clinical relapse can be detected using standard biochemical and haematological measurements and novel biomarkers of complement activation or tissue injury. An embedded qualitative study of patients, both those who withdraw and decide not to withdraw, will explore attitudes towards treatment and its withdrawal.

For peer review only

For peer review only - http://bmjopen.bmj.com/site/about/guidelines.xhtml

REFERENCES

1. Fremeaux-Bacchi V, Fakhouri F, Garnier A, et al. Genetics and outcome of atypical hemolytic uremic syndrome: a nationwide French series comparing children and adults. *Clin J Am Soc Nephrol* 2013, 8(4):554-562.
2. Noris M, Remuzzi G. Thrombotic microangiopathy after kidney transplantation. *Am J Transplant* 2010;10(7):1517-1523.
3. Johnson S, Stojanovic J, Ariceta G, et al. An audit analysis of a guideline for the investigation and initial therapy of diarrhea negative (atypical) hemolytic uremic syndrome. *Pediatric nephrology (Berlin, Germany)* 2014;29(10):1967-1978.
4. Sheerin NS, Kavanagh D, Goodship TH, et al. A national specialized service in England for atypical haemolytic uraemic syndrome-the first year's experience. *QJM* 2016;109(1):27-33.
5. Le Quintrec M, Zuber J, Moulin B, et al. Complement genes strongly predict recurrence and graft outcome in adult renal transplant recipients with atypical hemolytic and uremic syndrome. *Am J Transplant* 2013;13(3):663-675.
6. Legendre CM, Licht C, Muus P, et al. Terminal complement inhibitor eculizumab in atypical hemolytic-uremic syndrome. *The New England journal of medicine* 2013;368(23):2169-2181.
7. Greenbaum LA, Fila M, Ardissino G, et al. Eculizumab is a safe and effective treatment in pediatric patients with atypical hemolytic uremic syndrome. *Kidney international* 2016;89(3):701-711.
8. Fakhouri F, Hourmant M, Campistol JM, et al. Terminal Complement Inhibitor Eculizumab in Adult Patients With Atypical Hemolytic Uremic Syndrome: A Single-Arm, Open-Label Trial. *Am J Kidney Dis* 2016; 68(1):84-93.
9. Licht C, Greenbaum LA, Muus P, et al. Efficacy and safety of eculizumab in atypical hemolytic uremic syndrome from 2-year extensions of phase 2 studies. *Kidney international* 2015; 87(5):1061-1073.
10. European Medicines Agency: http://www.ema.europa.eu/docs/en_GB/document_library/EPAR_-_Assessment_Report_-_Variation/human/000791/WC500119185.pdf. 2011.
11. FDA: http://www.fda.gov/AboutFDA/CentersOffices/OfficeofMedicalProductsandTobacco/CDER/ucm273089.htm.
12. https://www.nice.org.uk/guidance/hst1/resources/eculizumab-for-treating-atypical-haemolytic-uraemic-syndrome-1394895848389
13. Struijk GH, Bouts AH, Rijkers GT, et al. Meningococcal sepsis complicating eculizumab treatment despite prior vaccination. *Am J Transplant* 2013;13(3):819-820.
14. McNamara LA, Topaz N, Wang X, et al. High Risk for Invasive Meningococcal Disease Among Patients Receiving Eculizumab (Soliris) Despite Receipt of Meningococcal Vaccine. *MMWR Morb Mortal Wkly Rep* 2017;66(27):734-737.

For peer review only

For peer review only - http://bmjopen.bmj.com/site/about/guidelines.xhtml

15. Carr R, Cataland SR. Relapse of aHUS after discontinuation of therapy with eculizumab in a patient with aHUS and factor H mutation. *Annals of hematology* 2013;92(6):845-846.

16. Ardissino G, Testa S, Possenti I, et al. Discontinuation of eculizumab maintenance treatment for atypical hemolytic uremic syndrome: a report of 10 cases. *Am J Kidney Dis* 2014;64(4):633-637.

17. Wijnsma KL, Duineveld C, Volokhina EB,et al. Safety and effectiveness of restrictive eculizumab treatment in atypical haemolytic uremic syndrome. *Nephrol Dial Transplant* 2018;33(4):635-645.

18. Fakhouri F, Fila M, Hummel A, et al. Eculizumab discontinuation in children and adults with atypical haemolytic uremic syndrome: a prospective multicentric study. *Blood* 2020.

19. Johnson VE, Cook JD: Bayesian design of single-arm phase II clinical trials with continuous monitoring. *Clin Trials* 2009; 6(3):217-226.

20. https://biostatistics.mdanderson.org/SoftwareDownload/SoftwareFiles/BayesFactorBinary/UsersGuide_BayesFactorBinary.pdf

21. Glaser BG. The Constant Comparative Method of Qualitative Analysis. *Social Problems* 1965;12(4):436-45.

22. Drummond MF, O'Brien B, Stoddart GL, et al. Basic Types of Economic Evaluation. In: *Methods for economic evaluation of health care programmes.* edn. Oxford University Press; 2005.

For peer review only

Acknowledgements

The authors would like to thank Dr Colin Muirhead who was responsible for the statistical proposal in the initial grant application.

The authors would also like to thank all the Principal Investigators for their contribution and support, the research nurses, the members of the TSC and DMC and all the patients involved in the SETS aHUS Trial.

Contributors

Professor Neil Sheerin is the Chief Investigator and senior author of the SETS aHUS design and has led on grant acquisition and protocol development. Professor David Kavanagh, Dr Sally Johnson, Dr Ewin Kwan Soon Wong, Mr Len Woodward, Ms Jan Lecouturier, Dr Thomas Chadwick, and Dr Yemi Oluboyede are co-applicants of the grant and contributed to the design of the trial and protocol development. Dr Sonya Carnell and Ms Sarah Dunn are part of the trial management team and contributed to protocol development. Mr Eoin Moloney is a Health Economist and contributed to protocol development. Mr Andy Bryant is a research statistician and contributed to protocol development. Mr Christopher Weetman is a data manager and contributed to protocol development. Dr Michal Malina and Dr Victoria Brocklebank are part of the National Renal Complement Therapeutics Centre and have played key roles in the running of the SETS aHUS trial.

This paper was drafted from the current approved version of the protocol by the corresponding author SD; all contributing authors commented and amended drafts of the paper. All contributing authors read and approved the final manuscript.

Funding

The SETS aHUS Trial is funded by the National Institute for Health Research (NIHR) Health Technology Assessment (HTA) Programme (project number 15/130/94). The views expressed are those of the authors and not necessarily those of the NIHR or the Department of Health and Social Care.

Conflict of interests

SB has received honoraria for sitting on advisory boards for Alexion and Novartis. DK is a director of and scientific advisor to Gyroscope Therapeutics. DK received advisory board payments from Idorsia, Novartis, ChemoCentryx, Alexion, Apellis, Biomarin and Sarepta. DK's spouse works for GSK. MM has received honoraria for educational talks and honorarium for national lead of aHUS registry, both from Alexion and travel expenses from Alexion. EKSW has received honoraria for lectures and/or advisory boards for Alexion Pharmaceutical, Biocryst and Novartis. LW has received expenses, honoraria and fees for advisory board participation and talks from Alexion and Roche. NS has given lectures or sat on advisory boards for Alexion Pharmaceutical, Roche, Astra Zeneca and Novartis, no personal honoraria, all payments made to the department.

Patient and public involvement

For peer review only - http://bmjopen.bmj.com/site/about/guidelines.xhtml

Patients and/or the public were involved in the design, or conduct, or reporting, or dissemination plans of this research. Refer to the Methods section for further details.

Patient consent for publication

Not required.

Provenance and peer review

Not commissioned; externally peer reviewed during funding application process.

Figure Legends

Figure 1. Data Collection Time Points for Withdrawal cohort

Figure 2. Data Collection Time Points for Health Economics Cohort

Figure 3. Criteria for diagnosis of aHUS relapse

Figure 4. Haematological remission definition and maintenance

For peer review only

	Central Pre-Screen	Site Screen and Consent	Final Infusion	Withdrawal Phase																																
Month	0	0	0	1					2		3		4		5		6		7	8	9	10	11	12	13	14	15	16	17	18	19	20	21	22	23	24	Unscheduled Visit
Visit Number	N/A	N/A	1	2	3	4	5	6	7	8	9	10	11	12	13	14	15	16	17	18	19	20	21	22	23	24	25	26	27	28	29	30	31	32	33	34	
Genetic Eligibility	X																																				
Medical History Review		X																																			
Informed consent		X																																			
Eligibility Checklist Completion		X																																			
Physical Examination		X																						X												X	X
Height & Weight		X																																			
Pregnancy test		X																																			
Eculizumab Infusion			X																																		
meningococcal prophylaxis			X	X																																	
urine analysis training				X																																	
Vital Signs		X		X	X	X	X	X	X	X	X	X	X	X	X	X	X	X	X	X	X	X	X	X	X	X	X	X	X	X	X	X	X	X	X	X	X
Concomitant medication Review		X		X	X	X	X	X	X	X	X	X	X	X	X	X	X	X	X	X	X	X	X	X	X	X	X	X	X	X	X	X	X	X	X	X	X
Renal Function (Creatinine & GFR)		X		X	X	X	X	X	X	X	X	X	X	X	X	X	X	X	X	X	X	X	X	X	X	X	X	X	X	X	X	X	X	X	X	X	X
Liver Function Tests		X		X	X	X	X	X	X	X	X	X	X	X	X	X	X	X	X	X	X	X	X	X	X	X	X	X	X	X	X	X	X	X	X	X	X
Haemolysis markers (full blood count & LDH)		X		X	X	X	X	X	X	X	X	X	X	X	X	X	X	X	X	X	X	X	X	X	X	X	X	X	X	X	X	X	X	X	X	X	X
Electrolyte profile (U&Es)		X		X	X	X	X	X	X	X	X	X	X	X	X	X	X	X	X	X	X	X	X	X	X	X	X	X	X	X	X	X	X	X	X	X	X
Haptoglobin & Blood film		X		X				X				X						X						X						X						X	X
Urine PCR		X		X				X				X						X						X						X						X	X
Biomarkers and complement		X		X	X	X		X		X		X						X			X			X						X						X	X

activation sample																																					
Home Urinalysis		X		Daily					3 times per week																												
Home Urinalysis diary review					X	X	X	X	X	X	X	X	X	X	X	X	X	X	X	X	X	X	X	X	X	X	X	X	X	X	X	X	X	X	X	X	
Adverse Events					X	X	X	X	X	X	X	X	X	X	X	X	X	X	X	X	X	X	X	X	X	X	X	X	X	X	X	X	X	X	X	X	X
EQ5D & SF36				X		X					X						X				X			X						X						X	
Health care Utilisation questionnaire				X		X					X						X				X			X						X						X	
Time & Travel Questionnaire																														X							

For peer review only

For peer review only

For peer review only - http://bmjopen.bmj.com/site/about/guidelines.xhtml

	Follow - up							
month	1		3	6	9	12	18	24
visit number	1	2	3	4	5	6	7	8
Medical History Review	X							
Informed consent	X							
Eligibility Checklist	X							
EQ5D & SF36	X	X	X	X	X	X	X	X
Health care Utilisation questionnaire	X	X	X	X	X	X	X	X
Time & Travel Questionnaire							X	

For peer review only

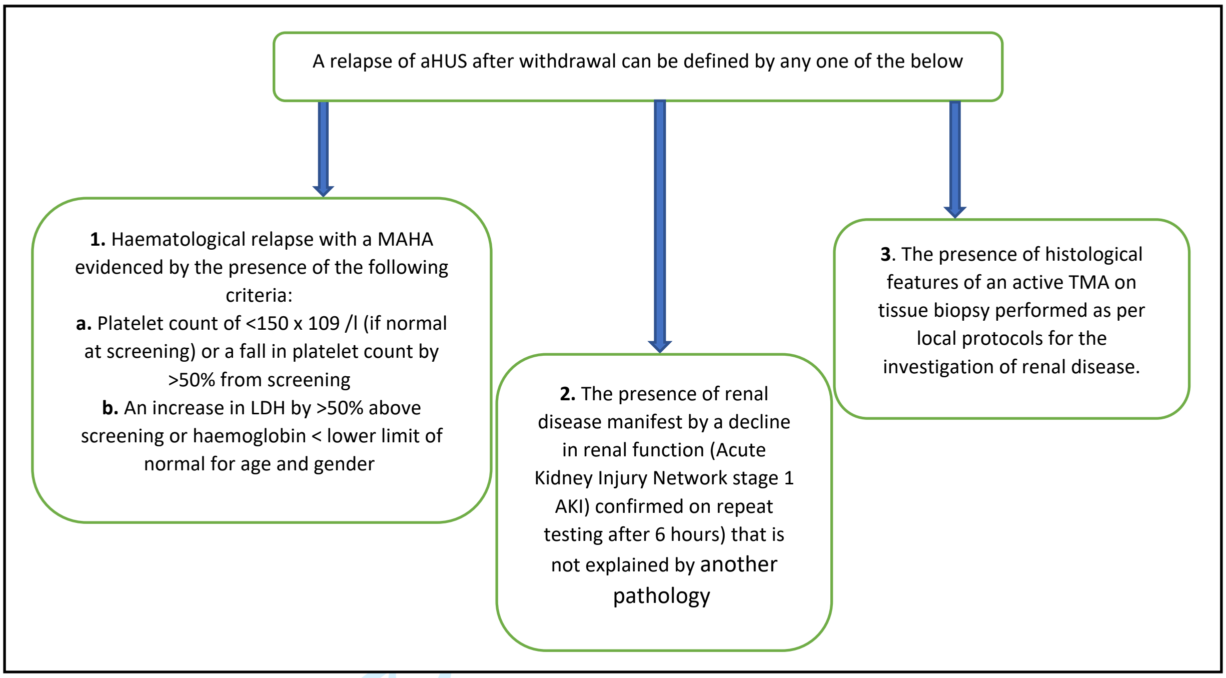

For peer review only

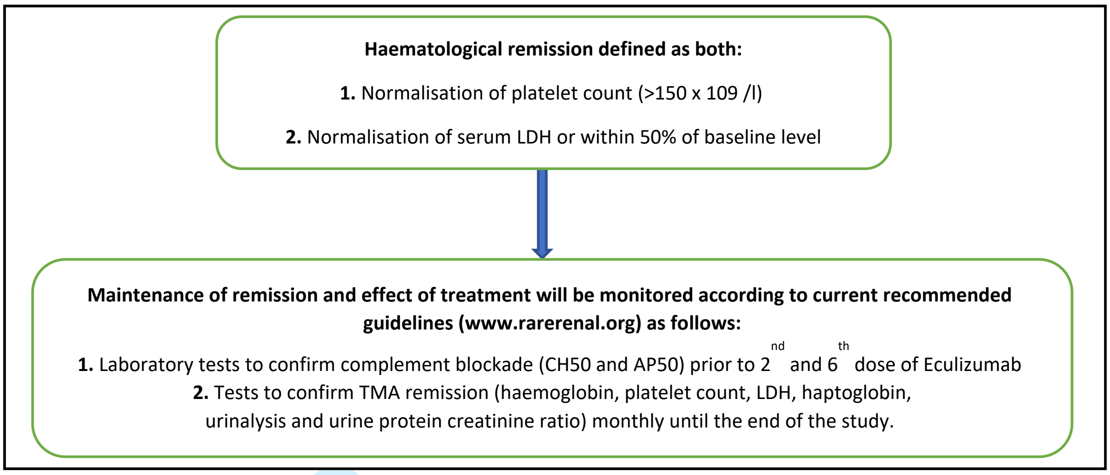

For peer review only

[Trust Headed Paper]

SETS aHUS: **S**topping **E**culizumab **T**reatment **S**afely in **aHUS**

Participant Information Sheet (aged 16+)

Version 3.0, 08.02.19

Invitation

You are being invited to take part in a research study. Please read the following information to help you decide if you want to take part. We would like you to understand why we are doing this research and what it means for you. You do not need to make a decision straight away, so please feel free to talk to others about the study if you wish. Please ask us if there is anything that is not clear or if you want to know more.

Please remember that you do not have to take part and your normal healthcare will not be affected in any way, whatever you decide.

Part 1

What is the purpose of this study?

Atypical Haemolytic Uraemic Syndrome (aHUS) is a rare disease. When aHUS occurs the cells that line the blood vessels are damaged and are no longer able to stop blood from clotting. Blood clots form in small vessels, particularly in the kidney, leading to problems with kidney function. Most cases are due to abnormalities in a part of the immune system called the complement system. These abnormalities lead to excessive activation of the complement system, which is responsible for the cell damage and blood clots.

Current standard treatment for aHUS involves a long-term intravenous injection of a drug called Eculizumab every 2 weeks. Eculizumab blocks the body's complement system and its ability to damage its own vulnerable cells.
Research has shown that Eculizumab is effective in the treatment of aHUS, but the recommendation that Eculizumab treatment should be lifelong is not based on strong evidence and may not be necessary for many patients.

This study hopes to provide evidence for an alternative strategy for treatment of aHUS based on monitoring and treatment re-introduction rather than continuous Eculizumab treatment.

Overall aim: To establish an alternative and safe treatment strategy for patients with aHUS that includes withdrawal of Eculizumab treatment.
Thank you for reading so far – if you are still interested, please read the rest of this leaflet which gives more detailed information about the trial and what will happen if you decide to take part.

For peer review only - http://bmjopen.bmj.com/site/about/guidelines.xhtml

[Trust Headed Paper]

Why have I been chosen to take part?

You have been asked to take part in the study because you have Atypical Haemolytic Uraemic Syndrome and are currently receiving Eculizumab treatment. We are hoping to recruit 30 patients to the withdrawal study and an additional 20 patients who will remain on Eculizumab and complete a series of questionnaires only.

What would taking part involve?

Taking part would involve stopping your Eculizumab treatment and attending 34 safety monitoring visits at hospital over the course of 24 months during which we will assess that you are safe to continue to remain off medication. If you currently receive your treatment at home you would need to be willing to attend the hospital to complete the safety visits.
You will be required to test your urine at home and document the results daily (for the first month), and then three times per week for the remaining 23 months, in a patient diary provided to you by the study team. You will also complete questionnaires on 8 occasions relating to quality of life and measure use of NHS resources and your own out of pocket expenses related to health care.
If you do not want to stop your Eculizumab treatment but still want to participate you can also complete study questionnaires that relate to your quality of life, and measure use of NHS resources and your own out of pocket expenses related to health care. You would only be asked to complete these on 8 occasions over the 24-month period.

There is also a linked interview study that you can take part in whether you decide to withdraw from treatment or continue with Eculizumab treatment. If you would like to receive information about this study you can consent to have your contact details passed to the research team for them to contact you about this.

What are the possible benefits of taking part?

It might mean that you will no longer need to take the Eculizumab treatment and face any potential risks or side effects associated with treatment. Patients are about a thousand times more likely to develop a serious, potentially life threatening infection with meningococcus, a bug that causes meningitis or sepsis. Vaccination, and even antibiotics, do not give complete protection from this. Other serious problems have also been reported in patients taking Eculizumab but we cannot be certain that Eculizumab was the cause of these problems.

Being in the study will mean that you will no longer need bi-weekly infusions and will not have to continue taking additional antibiotics to prevent infection. However, it is possible that you could need to restart and continue to receive Eculizumab treatment if a relapse was to occur.

For peer review only - http://bmjopen.bmj.com/site/about/guidelines.xhtml

[Trust Headed Paper]

What are the possible risks of taking part?

The withdrawal of Eculizumab treatment could lead to a relapse of atypical HUS and relapse associated complications. When a relapse is diagnosed, your Eculizumab treatment will be restarted within 24 hours of presentation. It is essential that you present to a hospital with your patient card as soon as you begin to feel unwell or the home urine test shows an increase in the level of blood. This is to ensure that the Eculizumab is re-started as soon as possible to reduce the likelihood of kidney damage and associated complications. If your treatment is re-started, you will receive your first infusion in the hospital. You will also have the antibiotics restarted to protect you from infection. If you need to be put back on to Eculizumab treatment you can decide to receive your infusions in hospital or at home. This can be arranged with your clinical team if you relapse while in the study.

If a relapse occurs this could lead to:

- A drop in kidney function
- Other problems related to the disease which can affect organs such as the pancreas or nervous system

The evidence that is available at the moment suggests that if Eculizumab is reintroduced quickly these problems can be avoided. This is not proven, and this study will test whether this is true.

If, during the trial, you decide to travel outside of the country, we ask that you only travel to countries where Eculizumab is available, so treatment can be re-started immediately as required. You **MUST** first inform your hospital to check whether Eculizumab is available in the country that you plan to travel to and inform them of the dates that you will be outside of the country. While the cost of the Eculizumab will be covered by NHS England you must ensure that you have appropriate travel insurance and inform your insurance company that you are taking part in the trial. This is to ensure that all other treatment costs and hospital stays abroad are covered by your own insurance should you relapse. We have developed a travel guide to be followed should you decide to travel outside of the country while you are participating in the withdrawal study.

To minimise the risks of taking part in the study, we will only include you in the study if you:

- Have been on Eculizumab treatment for at least 6 months;
- Are in remission;
- Have a stable kidney function;
- Are willing to attend for safety monitoring assessments;
- Are willing to travel only to countries that can supply Eculizumab (to be confirmed with co-ordinating centre prior to travel);
- Are able to perform and record self-monitoring urinalysis.

For peer review only - http://bmjopen.bmj.com/site/about/guidelines.xhtml

[Trust Headed Paper]

Sexually active females of child bearing age must:

- Have a negative pregnancy test at screening and be using an effective contraception for the duration of the study (please ask your doctor/nurse).

  OR

- fulfil one of the following criteria:
  - Be post-menopausal
  - Have undergone surgical sterilisation

You will not be able to take part if you:

- have lost a previous transplant kidney to recurrent aHUS;
- Are currently or are planning pregnancy;
- Are unable to comply with safety monitoring assessments;
- Are currently participating in another clinical trial (not including participation in aHUS registries)

What will happen to me if I take part?

If you consent to withdraw from Eculizumab Treatment, you will complete the following visits:

Screening Visit

You will be asked to attend a screening visit at the hospital or clinic where you usually receive your Eculizumab treatment or if you receive your treatment at home, you will be asked to attend the hospital where your clinician is based. If you decide to take part you will complete a consent form. You will be given a unique patient ID number that will be written on your consent form and all questionnaires and samples that you provide in the study. This ID number will only be linked to you at your hospital and so nobody outside of your direct clinical care team will know that the information and samples belong to you.
If you are a female of child bearing age you will be required to have a pregnancy test at the screening visit after you have signed the consent form. You will receive your last Eculizumab infusion at the screening visit. Please be aware that you will continue to take your meningococcal prophylaxis for 4 weeks following this last Eculizumab infusion.
We will also collect information from your medical notes about your medical history after you have signed the consent form.

Baseline Visit

Your baseline visit will be 2 weeks (+/-2 days) after your screening visit on the day that you would usually receive your next dose of Eculizumab. You will not receive the infusion but will

For peer review only - http://bmjopen.bmj.com/site/about/guidelines.xhtml

attend your usual hospital or clinic to have your blood pressure and temperature checked. You will also have a blood and urine test and complete questionnaires about your health.

Study Visits

You will be reviewed at the hospital weekly for the first month, then alternate weeks until month 6, then monthly thereafter until the end of the study period (month 24).
At each study visit you will have a blood and urine test and answer questions about how your health has been since your last visit.

You will also be required to complete questionnaires at your hospital visits twice in month 1, and once in the following months, 3, 6, 9, 12, 18 & 24. These will ask about your health, quality of life, medications and about use of NHS services and your out of pocket expenses relating to health care. It should take between 20 and 30 minutes to complete these questionnaires each time.

Overall you will be attending 34 visits over the 24 month period which includes the screening and baseline visit. You will not be required to attend more visits than you would usually if you were to stay on your current treatment. If you currently receive your Eculizumab infusions at home you will need to come in to the hospital for the study visits as these cannot be carried out at your home.

You will be required to carry out urine tests at home and to document this in a patient diary. You will be given a 30-minute training session during your baseline visit that will explain everything that you will need to do to carry this out at home.

You will also be given a small card to carry which will include information on the trial. If you attend **ANY** hospital or clinic for treatment outside of your scheduled follow-up visits you **MUST** give the doctor this card so that they are able to contact the trial team.

If you continue with your Eculizumab treatment and consent to completing the questionnaires only;
You will receive the questionnaires by post on 8 occasions over the 24-month period. This should take you around 20-30 minutes to complete. You will be required to post the completed questionnaires back to the research team at Newcastle University using the pre-paid envelope supplied. The questionnaires will not contain any identifiable information relating to you as your unique patient ID will be written on the questionnaires when you receive them.

Will participating in research affect my treatment?

If you decide to take part in the withdrawal component of the trial then you will no longer receive your Eculizumab treatment. If you decide to take part in the questionnaire part of the trial only, your Eculizumab treatment will remain the same.

For peer review only - http://bmjopen.bmj.com/site/about/guidelines.xhtml

[Trust Headed Paper]

What will happen if I don't want to carry on with the study?

You may withdraw from the study at any time without giving a reason. If you decide to go back on to Eculizumab treatment we would like you to continue to be followed up by the study team. You may wish to withdraw completely from the study and further follow up and so we would not collect any further information about you to be used in the study. However, we would like to use the information and blood samples previously provided. If you decide that you don't want any of the information or blood samples already provided to be used in the study please contact a member of the study team so it can be removed from analysis.

Will I be paid for taking part?

If you receive re-imbursement for travel to clinics this will continue. If you currently receive your Eculizumab treatment at home, you may qualify to be reimbursed for travel to clinics for your follow up visits. Your doctor can give you more information about this. You will not be paid for taking part in this study.

Part 2

Will my GP be told about my involvement in this study?

If you decide to take part in this study and consent to have your GP informed, then we will inform your GP. Your participation in the study will also be noted in your medical records.

Will my taking part in research be kept confidential?

All of the data we collect will be kept strictly confidential and in accordance with the General Data Protection Regulation (GDPR).

The Newcastle Upon Tyne Hospitals NHS Foundation Trust (NUTH) is the sponsor for this study based in the United Kingdom and will act as the "data controller" for this study. **They are responsible for looking after your information and using it properly.** This study is managed on behalf of the sponsor by the Newcastle Clinical Trials Unit (NCTU) who will act as the "data processor". As data processor, NCTU are responsible for processing personal data on behalf of the data controller. The Newcastle Clinical Trials Unit based at Newcastle University would like to receive a copy of your consent form for safety purposes. This will be destroyed once it has been reviewed. No other identifiable information will be received by the Newcastle Clinical Trials Unit

Your rights to access, change or move your information are limited, as we need to manage your information in specific ways in order for the research to be reliable and accurate. If you withdraw from the study, we will keep the information about you that we have already

For peer review only - http://bmjopen.bmj.com/site/about/guidelines.xhtml

Newcastle University

SETS aHUS

[Trust Headed Paper]

obtained. To safeguard your rights, we will use the minimum personally-identifiable information possible.

You can find out more about how we use your information at www.newcastle-hospitals.org.uk/about-us/freedom-of-information_how-we-use-information.aspx

To find out more information about research and general use of patient information please refer to the Health Research Authority Website at www.hra.nhs.uk/information-about-patients

The local study team at [NHS site] will use your name, [NHS number/CHI number] and contacts details to contact you about the research study, and make sure that relevant information about the study is recorded for your care, and to oversee the quality of the study. Individuals from the sponsor, Newcastle Clinical Trials Unit and regulatory organisations may look at your medical and research records to check the accuracy of the research study. The local study team will pass these details to the sponsor or the Newcastle Clinical Trials Unit along with information collected from you and/or your medical records. The only people at sponsor or the Newcastle Clinical Trials Unit who will have access to information that identifies you will be people who need to audit the data collection process. The people who analyse the information will not be able to identify you and will not be able to find out your name, [NHS number/CHI number] or contact details.

The local study team at [NHS site] will keep identifiable information about you from this study for 5 years after the study has finished.

When you agree to take part in a research study, the information about your health and care may be provided to researchers running other research studies in this organisation and in other organisations. These organisations may be universities, NHS organisations or companies involved in health and care research in this country or abroad. Your information will only be used by organisations and researchers to conduct research in accordance with the UK Policy Framework for Health and Social Care Research and will not contain any personal identifiable information such as your name, [NHS number/CHI number] or contact details.

This information will not identify you and will not be combined with other information in a way that could identify you. The information will only be used for the purpose of health and care research and cannot be used to contact you or to affect your care. It will not be used to make decisions about future services available to you, such as insurance.

The blood samples that you provide will be sent to Newcastle University to be analysed and will have your unique ID number on them but no personal identifiable information attached. Once the study is finished, these samples will be stored at the Newcastle University biobank and may be used in future aHUS research. Please let us know if you do not want your samples used in future research.

For peer review only - http://bmjopen.bmj.com/site/about/guidelines.xhtml

[Trust Headed Paper]

What will happen to the results of the research study?

Whenever possible we will publish the results of our studies in scientific journals. We also plan to present data at scientific conferences. You will not be named in any publication or presentation of the study results. We would also like to send you a newsletter with a summary of our results. Please let the research team know if you want to receive the newsletter. Results will also be available at the end of the study on the atypical HUS website which can be found at the following web address:
http://www.atypicalhus.co.uk/

Who is organising and funding the research?

This study is funded by the National Institute for Health Research (NIHR). The study is sponsored and indemnified by the Newcastle Upon Tyne NHS Foundation Trust and indemnified by Newcastle University. The Newcastle Clinical Trials Unit is managing the study.

Who has reviewed the study?

All research in the NHS is looked at by an independent group of people called a NHS Research Ethics Committee (REC). This is to protect your interests. This study has been reviewed and given a favourable opinion by the North East – Tyne & Wear South Research Ethics Committee and has been approved by the NHS Health Research Authority (HRA). The study has also been given approval by the Medicines and Healthcare products Regulatory Agency (MHRA).

What if relevant new information becomes available?

The study team will ensure that you are receiving the most appropriate and up to date medical care that you require.

What if something goes wrong?

If you have a concern about any aspect of the study please contact your local doctor (see contact details below). Alternatively, you can contact one of the researchers running this study and discuss your concerns.

Your local contact people for the study are:

Contact Details of local PI:	Contact details of local Research Nurse:
Name:	Name:
Address:	Address:
Phone:	Phone:
Email:	Email:

For peer review only - http://bmjopen.bmj.com/site/about/guidelines.xhtml

[Trust Headed Paper]

The Newcastle Trial Team contact is:

Contact Details of Trial Manager:
Name: Sarah Dunn
Address: 1-2 Claremont Terrace,
NCTU, Newcastle University, NE2 4AE
Phone: 0191 208 2521
Email: sarah.dunn2@ncl.ac.uk

If you are still unhappy and wish to complain formally and confidentially you can do this through the NHS complaints procedure by speaking to a member of the PALS (Patient Advise and Liaison Service) on 0800 0320 202 or by visiting www.PALS.nhs.uk.

In the event that something goes wrong and you are harmed during the research due to someone's negligence, then you may have grounds for a legal action for compensation against Newcastle upon-Tyne Hospitals NHS Foundation Trust but you may have to pay your legal costs.

How have patients and the public been involved in this study?

Our patient representative group has helped to design the study and study documents.

The group will meet when the trial is near completion to have input into the interpretation of the results. Their views of withdrawal and the safety results from the study will be considered. This will inform the publication of results. The groups views on the dissemination of results will also be considered.

Thank you for taking time to read this information sheet

For peer review only - http://bmjopen.bmj.com/site/about/guidelines.xhtml

SETS aHUS: Stopping Eculizumab Treatment Safely in aHUS

The holder of this card has a rare disease known as atypical Haemolytic Uraemic Syndrome and is taking part in a clinical trial to assess the safe withdrawal of Eculizumab treatment. Because of this, the holder of this card may suffer a relapse.

If the holder presents unwell, however minor the illness, please evaluate immediately and obtain the following Laboratory investigations as their Eculizumab treatment and prophylactic antibiotics may need to be re-started as soon as possible and within 24hrs:

• U&E • FBC • LDH

If the results are abnormal, immediately contact the local medical team and refer to the UK National aHUS Service website for advice. Contact details on reverse side.

For peer review only - http://bmjopen.bmj.com/site/about/guidelines.xhtml

SETS aHUS: Stopping Eculizumab Treatment Safely in aHUS

Patients Local Medical team: +44 (0)…………………………..

UK National aHUS Service: +44 (0)191 2820385

(from 9am to 5pm GMT)

Outside of these hours, please contact the Newcastle Hospitals Switchboard and request to speak to the clinician on call for the aHUS Service on +44 (0)191 233 6161

www.atypicalhus.co.uk

For peer review only - http://bmjopen.bmj.com/site/about/guidelines.xhtml

Primary registry and trial identifying number	ISRCTN<br>ISRCTN17503205
Date of registration in primary registry	20 April 2018
Secondary identifying numbers	EudraCT: 2017-003916-37
Source(s) of monetary or material support	NIHR HTA
Primary sponsor	Newcastle Upon Tyne Hospitals NHS FT – Christopher Price<br>christoper.price6@nhs.uk
Secondary sponsor(s)	N/A
Contact for public queries	Trial Manager – Sarah Dunn sarah.dunn2@newcastle.ac.uk
Contact for scientific queries	Chief Investigator – Professor Neil Sheerin neil.sheerin@newcastle.ac.uk
Public title	Stopping Eculizumab Treatment Safely in aHUS (SETS aHUS)
Scientific title	Multicentre, open label, prospective, single arm study of safety impact of Eculizumab withdrawal
Countries of recruitment	England and Scotland
Health condition(s) or problem(s) studied	atypical Haemolytic Uraemic Syndrome
Intervention(s)	Withdrawal of Eculizumab
Key inclusion and exclusion criteria	Key inclusion criteria:<br>• Age ≥2+ years of age,<br>• On Eculizumab treatment for at least 6 months,<br>• In remission with no evidence of ongoing microangiopathic haemolytic anaemia (MAHA) activity at screening defined by:<br>- Platelet count > lower limit of normal as determined by local reference range,<br>- Lactate Dehydrogenase (LDH) <x2 upper limit of normal as determined by local lab reference ranges,<br>• Normal renal function or Chronic Kidney Disease (CKD) stages 1-3,<br>• Absence of decline of renal function confirmed by review of available assessments of renal function for the preceding 6 months by the Chief

	Investigator (CI) and clinical members of the Trial Management Group (TMG).<br>Key Exclusion Criteria:<br>• Severe non-renal disease manifestations at initial presentation with aHUS, which in the opinion of the Chief Investigator and/or the clinical members of the TMG makes the risk of treatment withdrawal unacceptable,<br>• Current or planned pregnancy within the study duration,<br>• Unable to give informed consent or assent, or unable to obtain parent/guardian consent if under 16 years of age,<br>• Current participation in another clinical trial (not including participation in aHUS registries),<br>• Severe, uncontrolled hypertension (systolic blood pressure >160 mmHg) that is likely to induce at TMA.
Study type	Allocation: non-randomized<br>Masking: open label<br>Primary purpose: Safety<br>Phase IIb
Date of first enrolment	November 2018
Target sample size	50: 30 withdrawal and 20 non-withdrawal
Recruitment status	Recruiting
Primary outcome(s)	To determine the safety of Eculizumab withdrawal in patients with aHUS
Key secondary outcomes	1. The effectiveness of a monitoring protocol to detect disease relapse following withdrawal of Eculizumab assessed by:<br>1.1. The proportion of patients who relapse and restart Eculizumab without the development of a TMA-related SAE<br>1.2. The time from the first clinical feature (symptom, positive urinalysis or laboratory result) of a relapse of TMA and the re-introduction of Eculizumab<br>This outcome is ongoing and not measured at any particular timepoint<br>2. The relapse rate after withdrawal of Eculizumab as determined by the proportion of patients who relapse after Eculizumab is withdrawn. This outcome is ongoing and not measured at any particular time point. A patient could relapse at any point in the 2 years participation period.<br>3. The proportion of patients, currently on long-term treatment with Eculizumab, who can be maintained off treatment. This outcome is measured at the end of the trial when all relapse data is collected. A patient could relapse at any point in the 2 years participation period.<br>4. The period from withdrawal to relapse in those patients who restart treatment. This outcome is measured at the end of the trial when all relapse data is collected.<br>5. The change in estimated GFR as calculated by the CKD-EPI or modified Schwartz equations over the course of the study from baseline (day 0) to

	end of the study. This outcome is calculated at the end of the trial when all GFR data is collected. GFR data is collected at all 34 visits.<br>6. Important clinical and laboratory indicators of imminent relapse by review of reported symptoms, physical signs, urinalysis and laboratory results prior to the diagnosis of a relapse. This outcome will be assessed at the end of the trial when all relapse data is collected. Those who have relapsed will have all data preceding relapse reviewed to establish a relapse profile.<br>7. The costs and health outcomes (measured in terms of adverse events and quality-adjusted life years [QALYs]) for patients on standard care (not withdrawing from Eculizumab treatment) over the two-year trial duration:<br>7.1. Healthcare Utilisation Questionnaires for non-withdrawal participants at Day 0, 14, 70,154, 252, 336, 504 and 672.<br>7.2. Adverse Event Assessment at every visit from Day 7 (32 visits) for withdrawal participants.<br>8. QALYs estimated from responses to the EQ-5D-5L, and SF-36 and determinants of QALYs/utilities over the 24-month follow-up period. at Day 0, 14, 70,154, 252, 336, 504 and 672.<br>9. Model-based estimate of the costs and health consequences, with results presented in terms of cost per QALY gained, over the estimated lifetime of patients withdrawing from treatment compared with standard care.

review only

Reporting checklist for protocol of a clinical trial.

Based on the SPIRIT guidelines.

Instructions to authors

Complete this checklist by entering the page numbers from your manuscript where readers will find each of the items listed below.

Your article may not currently address all the items on the checklist. Please modify your text to include the missing information. If you are certain that an item does not apply, please write "n/a" and provide a short explanation.

Upload your completed checklist as an extra file when you submit to a journal.

In your methods section, say that you used the SPIRITreporting guidelines, and cite them as:

Chan A-W, Tetzlaff JM, Gøtzsche PC, Altman DG, Mann H, Berlin J, Dickersin K, Hróbjartsson A, Schulz KF, Parulekar WR, Krleža-Jerić K, Laupacis A, Moher D. SPIRIT 2013 Explanation and Elaboration: Guidance for protocols of clinical trials. BMJ. 2013;346:e7586

		Reporting Item	Page Number
**Administrative information**			
Title	#1	Descriptive title identifying the study design, population, interventions, and, if applicable, trial acronym	1
Trial registration	#2a	Trial identifier and registry name. If not yet registered, name of intended registry	3
Trial registration: data set	#2b	All items from the World Health Organization Trial Registration Data Set	3, 4, 6, 7, 8, 9, 13, 16, 20
Protocol version	#3	Date and version identifier	3
Funding	#4	Sources and types of financial, material, and other support	16

For peer review only

Roles and responsibilities: contributorship	#5a	Names, affiliations, and roles of protocol contributors	1
Roles and responsibilities: sponsor contact information	#5b	Name and contact information for the trial sponsor	13 and 17
Roles and responsibilities: sponsor and funder	#5c	Role of study sponsor and funders, if any, in study design; collection, management, analysis, and interpretation of data; writing of the report; and the decision to submit the report for publication, including whether they will have ultimate authority over any of these activities	NA – sponsor and funder do not have involvement in these activities.
Roles and responsibilities: committees	#5d	Composition, roles, and responsibilities of the coordinating centre, steering committee, endpoint adjudication committee, data management team, and other individuals or groups overseeing the trial, if applicable (see Item 21a for data monitoring committee)	11, 12 and 13
**Introduction**			
Background and rationale	#6a	Description of research question and justification for undertaking the trial, including summary of relevant studies (published and unpublished) examining benefits and harms for each intervention	4
Background and rationale: choice of comparators	#6b	Explanation for choice of comparators	n/a – the trial withdraws patients from their current medication, no comparator required

For peer review only

Objectives	#7	Specific objectives or hypotheses	4 and 5
Trial design	#8	Description of trial design including type of trial (eg, parallel group, crossover, factorial, single group), allocation ratio, and framework (eg, superiority, equivalence, non-inferiority, exploratory)	4
**Methods: Participants, interventions, and outcomes**			
Study setting	#9	Description of study settings (eg, community clinic, academic hospital) and list of countries where data will be collected. Reference to where list of study sites can be obtained	6
Eligibility criteria	#10	Inclusion and exclusion criteria for participants. If applicable, eligibility criteria for study centres and individuals who will perform the interventions (eg, surgeons, psychotherapists)	6
Interventions: description	#11a	Interventions for each group with sufficient detail to allow replication, including how and when they will be administered	7
Interventions: modifications	#11b	Criteria for discontinuing or modifying allocated interventions for a given trial participant (eg, drug dose change in response to harms, participant request, or improving / worsening disease)	9

For peer review only

Interventions: adherence	#11c	Strategies to improve adherence to intervention protocols, and any procedures for monitoring adherence (eg, drug tablet return; laboratory tests)	9 – monitoring of home urinalysis
Interventions: concomitant care	#11d	Relevant concomitant care and interventions that are permitted or prohibited during the trial	7 – relating to meningococcal prophylaxis. No other concomitant medication requirements imposed by the trial
Outcomes	#12	Primary, secondary, and other outcomes, including the specific measurement variable (eg, systolic blood pressure), analysis metric (eg, change from baseline, final value, time to event), method of aggregation (eg, median, proportion), and time point for each outcome. Explanation of the clinical relevance of chosen efficacy and harm outcomes is strongly recommended	5
Participant timeline	#13	Time schedule of enrolment, interventions (including any run-ins and washouts), assessments, and visits for participants. A schematic diagram is highly recommended (see Figure)	7
Sample size	#14	Estimated number of participants needed to achieve study objectives and how it was determined, including clinical and statistical assumptions supporting any sample size calculations	6 and 10

For peer review only

Recruitment	#15	Strategies for achieving adequate participant enrolment to reach target sample size	6
**Methods: Assignment of interventions (for controlled trials)**			
Allocation: sequence generation	#16a	Method of generating the allocation sequence (eg, computer-generated random numbers), and list of any factors for stratification. To reduce predictability of a random sequence, details of any planned restriction (eg, blocking) should be provided in a separate document that is unavailable to those who enrol participants or assign interventions	NA- non-randomised trial
Allocation concealment mechanism	#16b	Mechanism of implementing the allocation sequence (eg, central telephone; sequentially numbered, opaque, sealed envelopes), describing any steps to conceal the sequence until interventions are assigned	NA- non-blinded
Allocation: implementation	#16c	Who will generate the allocation sequence, who will enrol participants, and who will assign participants to interventions	NA- non-randomised trial
Blinding (masking)	#17a	Who will be blinded after assignment to interventions (eg, trial participants, care providers, outcome assessors, data analysts), and how	N/A – non-blinded

For peer review only

Blinding (masking): emergency unblinding	#17b	If blinded, circumstances under which unblinding is permissible, and procedure for revealing a participant's allocated intervention during the trial	NA – non-blinded
**Methods: Data collection, management, and analysis**			
Data collection plan	#18a	Plans for assessment and collection of outcome, baseline, and other trial data, including any related processes to promote data quality (eg, duplicate measurements, training of assessors) and a description of study instruments (eg, questionnaires, laboratory tests) along with their reliability and validity, if known. Reference to where data collection forms can be found, if not in the protocol	7
Data collection plan: retention	#18b	Plans to promote participant retention and complete follow-up, including list of any outcome data to be collected for participants who discontinue or deviate from intervention protocols	9, 12 and 13
Data management	#19	Plans for data entry, coding, security, and storage, including any related processes to promote data quality (eg, double data entry; range checks for data values). Reference to where details of data management procedures can be found, if not in the protocol	13

For peer review only

Statistics: outcomes	#20a	Statistical methods for analysing primary and secondary outcomes. Reference to where other details of the statistical analysis plan can be found, if not in the protocol	10, 12 and 13
Statistics: additional analyses	#20b	Methods for any additional analyses (eg, subgroup and adjusted analyses)	12 and 13
Statistics: analysis population and missing data	#20c	Definition of analysis population relating to protocol non-adherence (eg, as randomised analysis), and any statistical methods to handle missing data (eg, multiple imputation)	12
**Methods: Monitoring**			
Data monitoring: formal committee	#21a	Composition of data monitoring committee (DMC); summary of its role and reporting structure; statement of whether it is independent from the sponsor and competing interests; and reference to where further details about its charter can be found, if not in the protocol. Alternatively, an explanation of why a DMC is not needed	11,12 and 13
Data monitoring: interim analysis	#21b	Description of any interim analyses and stopping guidelines, including who will have access to these interim results and make the final decision to terminate the trial	11 and 12
Harms	#22	Plans for collecting, assessing, reporting, and managing solicited	9 and 10

For peer review only

		and spontaneously reported adverse events and other unintended effects of trial interventions or trial conduct	
Auditing	#23	Frequency and procedures for auditing trial conduct, if any, and whether the process will be independent from investigators and the sponsor	13
**Ethics and dissemination**			
Research ethics approval	#24	Plans for seeking research ethics committee / institutional review board (REC / IRB) approval	14
Protocol amendments	#25	Plans for communicating important protocol modifications (eg, changes to eligibility criteria, outcomes, analyses) to relevant parties (eg, investigators, REC / IRBs, trial participants, trial registries, journals, regulators)	13
Consent or assent	#26a	Who will obtain informed consent or assent from potential trial participants or authorised surrogates, and how (see Item 32)	7
Consent or assent: ancillary studies	#26b	Additional consent provisions for collection and use of participant data and biological specimens in ancillary studies, if applicable	7
Confidentiality	#27	How personal information about potential and enrolled participants will be collected, shared, and maintained in order to protect confidentiality before, during, and after the trial	13

For peer review only

Declaration of interests	#28	Financial and other competing interests for principal investigators for the overall trial and each study site	16
Data access	#29	Statement of who will have access to the final trial dataset, and disclosure of contractual agreements that limit such access for investigators	13
Ancillary and post trial care	#30	Provisions, if any, for ancillary and post-trial care, and for compensation to those who suffer harm from trial participation	7 – post trial safety monitoring
Dissemination policy: trial results	#31a	Plans for investigators and sponsor to communicate trial results to participants, healthcare professionals, the public, and other relevant groups (eg, via publication, reporting in results databases, or other data sharing arrangements), including any publication restrictions	14
Dissemination policy: authorship	#31b	Authorship eligibility guidelines and any intended use of professional writers	16
Dissemination policy: reproducible research	#31c	Plans, if any, for granting public access to the full protocol, participant-level dataset, and statistical code	13
**Appendices**			
Informed consent materials	#32	Model consent form and other related documentation given to participants and authorised surrogates	N/A – not included but can be requested from the Trial Manager Sarah Dunn sarah.dunn2@newcastle.ac.uk

For peer review only

Biological specimens	#33	Plans for collection, laboratory evaluation, and storage of biological specimens for genetic or molecular analysis in the current trial and for future use in ancillary studies, if applicable	N/A – not included, laboratory manual can be requested from the Trial Manager Sarah Dunn sarah.dunn2@newcastle.ac.uk

None The SPIRIT Explanation and Elaboration paper is distributed under the terms of the Creative Commons Attribution License CC-BY-NC. This checklist can be completed online using https://www.goodreports.org/, a tool made by the EQUATOR Network in collaboration with Penelope.ai

For peer review only
